# Natural Deep Eutectic Solvents for the Extraction of Triterpene Saponins from *Aralia elata* var. *mandshurica* (Rupr. & Maxim.) J. Wen

**DOI:** 10.3390/molecules28083614

**Published:** 2023-04-21

**Authors:** Alyona A. Petrochenko, Anastasia Orlova, Nadezhda Frolova, Evgeny B. Serebryakov, Alena Soboleva, Elena V. Flisyuk, Andrej Frolov, Alexander N. Shikov

**Affiliations:** 1 Department of Technology of Pharmaceutical Formulations, St. Petersburg State Chemical Pharmaceutical University, 197376 Saint-Petersburg, Russia; 2 Laboratory of Analytical Biochemistry and Biotechnology, K.A. Timiryazev Institute of Plant Physiology RAS, 127276 Moscow, Russia; 3 Department of Plant Physiology and Biochemistry, St. Petersburg State University, 199034 Saint-Petersburg, Russia; 4 Chemical Analysis and Materials Research Centre, St. Petersburg State University, 198504 Saint-Petersburg, Russia

**Keywords:** *Aralia elata*, acid-based natural deep eutectic solvents (NADES), RP-UHPLC-MS, tandem mass spectrometry (MS/MS), targeted quantification, triterpene saponins

## Abstract

The roots of the medicinal plant *Aralia elata* are rich in biologically active natural products, with triterpene saponins constituting one of their major groups. These metabolites can be efficiently extracted by methanol and ethanol. Due to their low toxicity, natural deep eutectic solvents (NADES) were recently proposed as promising alternative extractants for the isolation of natural products from medicinal plants. However, although NADES-based extraction protocols are becoming common in routine phytochemical work, their application in the isolation of triterpene saponins has not yet been addressed. Therefore, here, we address the potential of NADES in the extraction of triterpene saponins from the roots of *A. elata*. For this purpose, the previously reported recoveries of *Araliacea* triterpene saponins in extraction experiments with seven different acid-based NADES were addressed by a targeted LC-MS-based quantitative approach for, to the best of our knowledge, the first time. Thereby, 20 triterpene saponins were annotated by their exact mass and characteristic fragmentation patterns in the total root material, root bark and root core of *A. elata* by RP-UHPLC-ESI-QqTOF-MS, with 9 of them being identified in the roots of this plant for the first time. Triterpene saponins were successfully extracted from all tested NADES, with the highest efficiency (both in terms of the numbers and recoveries of individual analytes) achieved using a 1:1 mixture of choline chloride and malic acid, as well as a 1:3 mixture of choline chloride and lactic acid. Thereby, for 13 metabolites, NADES were more efficient extractants in comparison with water and ethanol. Our results indicate that new, efficient NADES-based extraction protocols, giving access to high recoveries of triterpene saponins, might be efficiently employed in laboratory practice. Thus, our data open the prospect of replacing alcohols with NADES in the extraction of *A. elata* roots.

## 1. Introduction

*Aralia elata* var. *mandshurica* (Rupr. & Maxim.) J. Wen (1994) (syn. *A. elata*) [1] is a small, fast-growing tree or shrub that has shoots covered with thorns. It is also known as the “thorn tree” or “devil’s tree”. It is widely spread in Japan, eastern Siberia, South Korea and northeastern China [1,2]. The root and stem of *A. elata*, and especially their bark, are often used in traditional Chinese, Japanese and Korean medicine to treat rheumatic arthritis, soreness of the waist and knees, traumatic injury, lumps, abscesses, diabetes mellitus, epigastric pain, dysentery, insomnia and other inflammation-related disorders [2,3,4]. In the folk medicine of the Russian Far East region, *Aralia* roots were efficiently employed for the treatment of tonsillitis, flu, stomatitis and liver disorders. *Aralia* is the source of clinically proven adaptogens, i.e., extracts or individual natural products positively affecting the resilience and stress adaptability of organisms [5]. Indeed, multiple in vivo pharmacological studies have shown that tinctures and crude extracts prepared using the root of this plant demonstrated clear adaptogenic properties, manifested as enhanced stress tolerance [6] and pronounced gastroprotective [7,8], hepatoprotective [9,10], neuroprotective [11], hypolipidemic [12], antidiabetic [13], cardioprotective and antiarrhythmic [14] effects. In Russian clinical practice, the alcoholic tincture from the *Aralia* root is recommended for the treatment of arterial hypotension, asthenia and physical and mental fatigue [15].

To date, a total of approximately 200 compounds have been identified as biologically active constituents of *Aralia* extracts: terpenes, triterpene saponins, flavonoids, long-chain fatty acids and their esters, phenolic acids, coumarins, lignans, polyacetylenes, β-sitosterol, stigmasterol, adenosine, volatile oils and amino acids [2,16]. Thereby, triterpene saponins and terpenoids represent the key components isolated from *A. elata* [2]. These natural products underlie adaptogenic, hypoglycemic and hypertensive properties of *A. elata* isolates [9,12,15]. In the most efficient way, triterpene saponins can be extracted from the roots of this plant with water, alcohols (ethanol and methanol) and water–alcoholic mixtures [17,18,19,20]. Thus, the isolation of these biologically active metabolites on the industrial scale requires large volumes of organic solvents, which is associated with high costs, risk of environmental pollution and health concerns.

To reduce these negative effects, the use of natural deep eutectic solvents (NADES) was recently proposed as an alternative approach for the “green” extraction of natural products [21,22]. NADES represent the mixtures of hydrogen bond acceptors and hydrogen bond donors, which are typically known as commonly occurring natural constituents of the plant cell. The NADES most often considered are choline chloride, sugars, organic acids and amino acids [23]. The very important features of NADES are their low toxicity, high biodegradability and stability in mixtures, fairly low costs of components and well-established, straightforward synthetic procedures.

Among the commercially available NADES, acid-based solvents are the most representative group [23,24]. Such solvents have been successfully applied to the extraction of polar, semi-polar and non-polar compounds from crop and medicinal plants, fish-based products and seaweeds, e.g., iridoids [25], anthranoids and procyanidins [26,27,28], tanshinones [29], flavonoids [30,31], coumarins [32], tannins [33,34], curcumin [35], carotenoids, free fatty acids, polyunsaturated fatty acids [36], fucoxanthin [34], protein [37], alkaloids [38], terpenes [39], steroidal saponins [40] and triterpene saponins [41]. Remarkably, the latter group has still not yet been sufficiently addressed in the context of the application of NADES. Despite that NADES were successfully employed in the extraction of triterpene saponins, the systematic analysis of their efficiency in *Aralia* species is still missing. Moreover, the efficiency of this approach in application to their roots or individual parts is still mostly unknown. Therefore, to obtain a deeper insight into these aspects in the context of NADES extraction efficiency, in the present work, we systematically address the patterns of triterpene saponins in different parts of the *A. elata* root.

## 2. Results

### 2.1. Identification of Triterpene Saponins in A. elata Roots

At the first step, preliminary annotation relied on the data, acquired by reversed phase–ultra-high-performance liquid chromatography–electrospray ionization–quadrupole-time-of-flight–mass spectrometry (RP-UHPLC-ESI-QqTOF-MS) using a simulated sequential window acquisition of all theoretical mass spectra (SWATH) approach. For this, a comprehensive literature mining was accomplished and a representative list of triterpene saponins earlier annotated in *A. elata* roots was composed (Supplementary Information S1, Table S1). The core of this library relied on the work of Yu Xi et al., 2022 [2], which collected all known triterpene saponins, organized by different groups. Based on this list, a total of 104 metabolites could be annotated at the MS1 level in the samples of *A. elata* whole roots, bark and core. The exploration of the SWATH data revealed 25 hits, all of which could be assigned as prospective derivatives of caulophyllogenin, hederagenin and oleanolic and echinocystic acids (Table S2). However, the analysis of the MS1 data showed that annotated structures could potentially be not only the target saponins, but also the products of in-source fragmentation. This phenomenon is known in ESI-MS [42], and we addressed the probability of this scenario in detail.

One of the reasons for enhanced in-source fragmentation could be the design of the interface of the Shimadzu QqTOF mass spectrometer. Indeed, this phenomenon might be associated with not only heat-assisted desolvation (which is commonly employed to facilitate the ESI process), but also further droplet size degradation in a heated transfer capillary. Moreover, the instrument relied on Ar as a collision gas. Therefore, to reduce these effects and to verify our annotations, we employed another instrument—namely, Sciex TripleTOF 6600—for the acquisition of the MS/MS information. The interface of this instrument relies on curtain gas, which flows between the atmosphere pressure ionization (API) ion source and skimmer and further ion optics behind it [43]. Both of the curtain and collision gases were nitrogen, which resulted in lower energies of collision. With this mass spectrometer, we first performed two alternative experiments with a SWATH and DDA setup, in combination with a longer separation gradient, with the intention of obtaining fragmentation for all *m*/*z*, which could be considered as both prospective precursors and prospective in-source fragments in the data obtained using the Shimadzu instrument (listed in Table S3). This setup could present us an opportunity to address all unresolved issues by further targeted product ion scanning MS/MS experiments, if necessary.

All annotated triterpene saponins showed well-interpretable MS/MS spectra in the SWATH and DDA experiments, i.e., no targeted RP-UHPLC-QqTOF-MS/MS experiments were required. As was originally proposed, this analysis allowed the exclusion of most of the tentatively annotated species from the final list of the triterpene saponins discovered in the *A. elata* roots and the appearance of several new hits. The interpretation of the data was straightforward, although another QqTOF-MS instrument was used for the fragmentation experiments. Thus, only 20 metabolites of this class could be confirmed in the roots of *A. elata* based on their characteristic fragmentation patterns and the exclusion of false positive hits related to in-source fragmentation (Table S4). All these hits were in agreement with the spectral data acquired using the Shimadzu instrument, i.e., these analytes could be successfully re-annotated in the first dataset with both MS1 and MS2 information (Table 1). All finally annotated compounds were the derivatives of oleanolic acid (Figures S1–S14) and their patterns were in good agreement with the classical work of Yu Xi et al., 2022 [2]. Among the annotated compounds, a group of oleanolic acid *O*-glycosides of hexose and pentose was noted (compounds **1**, **2**, **8**, **9, 11**, **15**, **19** and **20**), along with several uronic acid derivatives (compounds **3**, **4**, **6**, **7**, **10**, **12**, **13**, **14**–**16** and **18**). In addition, the presence of isomeric structures characterized by identical *m*/*z* but different t_R_s (e.g., compounds **1** vs. **15** vs. **20**, **3** vs. **6**, **7** vs. **13**, **14** vs. **16** and **12** vs. **18**) should be noted among the annotated compounds. Although most of the annotated compounds have previously been reported in *A. elata* roots [2], the metabolites **1**, **3**, **4**–**7**, **13**, **15** and **20** were identified in this organ for the first time. Thus, the earlier characterized metabolites **1**, **4**, **5**, **6**, **15** and **20** were found in leaves, compound **3** was found in steams and **7** and **13** were found in leaves and buds [2,20].

### 2.2. Extraction of Triterpene Saponins with NADES

The freshly prepared NADES, i.e., the mixtures of choline chloride, organic acids (malic acid, lactic acid and oxalic acid) and sugar alcohol (sorbitol) were employed for the extraction of *A. elata* roots. Thereby, choline chloride acted as hydrogen bond acceptors, whereas malic acid, lactic acid, oxalic acid and sorbitol were used as hydrogen bond donors [23,24]. While studying the mixture of sorbitol and malic acid, Dai and coworkers noted that sugars and organic acids can behave similarly to donors and acceptors of hydrogen bonds [23].

**Figure 1:**
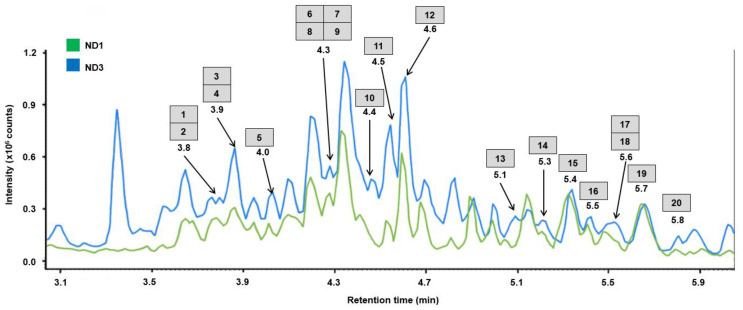
Total ion chromatograms (TICs) of the whole root extracts of *A. elata* prepared with the NADES, with the mixture of choline chloride and malic acid being 1:1 (ND1: green) and the mixture of choline chloride and lactic acid being 1:3 (ND3: blue). The analytes are listed as in Table 1.

All the tested NADES proved to be applicable for the extraction of triterpene saponins from the roots of *A. elata*, although their efficiency as extractants differed. The maximal number of extracted metabolites is annotated by the ND1 (choline chloride–malic acid: 1:1) and ND3 (choline chloride–lactic acid: 1:3) extracts. However, although all twenty saponins could be identified both in the ND1 and ND3, the relative contents of the individual metabolites in the extract ND1 were higher than in the ND3. The typical chromatograms of the ND1 and ND3 extracts are presented in Figure 1 (for the whole t_R_ range, please see Figure S15).

**Figure 2:**
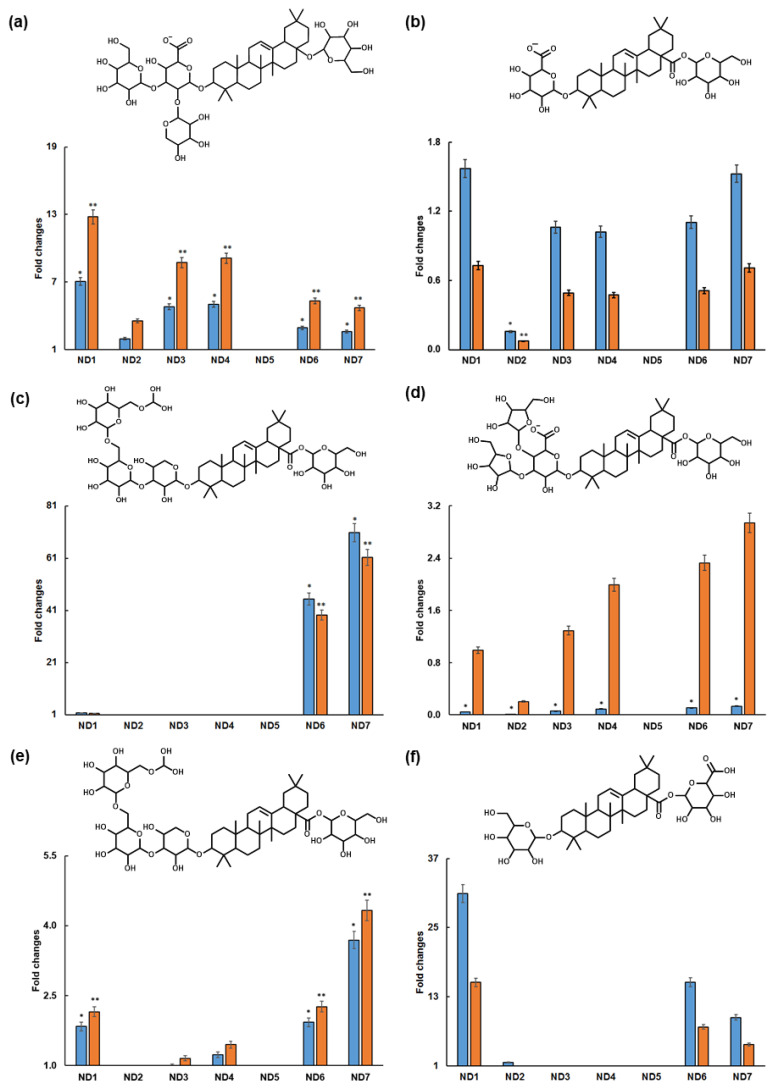
Structures and relative recoveries of **6** (kalopanax–saponin F isomer 2, (**a**)), **7** (calendulaglycoside C isomer 1, (**b**)), **9** (oleanolic acid-3-*O*-(methyldioxy-trihexopyranosyl-1-3-pentopyranosyl)-28-1-hexopyranosyl ester, (**c**)), **10** (araloside (**b**,**d**)), **12** (oleanolic acid-3-*O*-(hexosyl)-28-1-hexouronide ester isomer 1, (**e**)) and **13** (calendulaglycoside C isomer 2, (**f**)) expressed as the ratio (fold) in comparison to those observed in aqueous (blue) and ethanolic (orange) extracts. The compounds are numbered as in Table 1. ND1—NADES based on the choline chloride–malic acid mixture (molar ratio 1:1). ND2—NADES with the molar ratio of choline chloride and malic acid of 1:2. ND3—NADES with the molar ratio of choline chloride and lactic acid of 1:3. ND4—NADES with the molar ratio of choline chloride and lactic acid of 1:3 + 30% (*v*/*v*) water. ND6—NADES with the molar ratio of sorbitol and malic acid of 1:1 + 10% (*v*/*v*) water. ND7—NADES with the molar ratio of sorbitol and malic acid of 1:2 + 20% (*v*/*v*) water. *—the difference is that the recovery of the compound is statistically significant compared to the recovery of the compound in water extract (*p* ≤ 0.05); **—the difference is that the recovery of the compound is statistically significant compared to the recovery of the compound in ethanol extract (*p* ≤ 0.05).

To address the relative recovery efficiencies of the individual triterpene saponins achieved with different NADES, we normalized them to the specific recoveries observed in the aqueous and ethanolic extracts. Thus, the triterpene recoveries obtained with all NADES were expressed as fold changes relative to those observed in the aqueous and ethanolic extracts of the *A. elata* roots and root parts (Figure 2 and Table S4). Thus, the fold change exceeding one indicated better recoveries obtained with NADES, i.e., better extraction efficiency was demonstrated by these extractants. As can be seen in Figure 2, some NADES were inefficient for some (ND2, ND3 and ND4) or all (ND5) metabolites addressed, as can be judged from the comparison of their recoveries with aqueous or ethanolic extracts.

## 3. Discussion

Interestingly, NADES proved to be more efficient solvents for the extraction of thirteen triterpene saponin metabolites than water or ethanol. The recoveries of **5**–**10**, **12**–**16**, **18** and **19** (i.e., the relative contents of these compounds in corresponding extracts) were higher in NADES than in aqueous or ethanolic isolates. For example, NADES appeared to be more efficient than ethanol for the extraction of compound **10**. The saponin **5** showed better recoveries using NADES than using water, while ethanol was a weaker extractant. The highest recoveries of compounds **6**, **7**, **13**–**15** and **18**–**19** were found when the 1:1 mixture of choline chloride and malic acid (ND1) was applied. On the other hand, compound **5** was better recovered using the 30% *v*/*v* aqueous 1:3 mixture of choline chloride and lactic acid (ND4), whereas compounds **8**–**10**, **12** and **16** were better recovered using the 20% *v*/*v* aqueous 1:2 mixture of sorbitol and malic acid (ND7). Specifically, the relative recoveries of saponin **6** using the NADES extracts were higher than when using the ethanolic and aqueous extracts. Moreover, the recovery of compound **6** using ND1 was 13-fold higher than in the ethanolic extract and 9-fold higher than in the aqueous extract (Figure 2a). In contrast, the relative recoveries of compound **7** in NADES extracts appeared to be higher than in aqueous extracts, but not higher than ethanolic extracts, with the best effect observed using ND1 and ND7 (Figure 2b). The recoveries of saponin **9** using ND7 were 70-fold higher than the use of aqueous and 60-fold higher than the use of ethanolic extracts of *A. elata* (Figure 2c). The relative recoveries of compound **10** using NADES exceeded those when using ethanolic extracts, but not those when using the aqueous extracts (Figure 2d). This tendency was the clearest for ND7. The relative recoveries of compound **12** using NADES exceeded the contents of this compound recovered using ethanolic and aqueous extracts. This could be most clearly seen for ND7 (Figure 2e). On the other hand, the recoveries of triterpene saponin **13** were the highest using ND1 (Figure 2f). The pattern for compound **14** (araloside A isomer 1—Figure S16a) was similar to compound **9** (oleanolic acid-3-*O*-(methyldioxy-trihexopyranosyl-1-3-pentopyranosyl)-28-1-hexopyranosyl ester, Figure 2c); furthermore, the pattern for **18** (oleanolic acid-3-*O*-(hexosyl)-28-1-hexouronide ester isomer 2—Figure S16b) was similar to the pattern for the closely related structure of compound **12** (oleanolic acid-3-*O*-(hexosyl)-28-1-hexouronide ester isomer 1—Figure 2e). These similarities are explained by the similar structures of these compounds.

It is important to note that, despite the sufficient length of the LC gradient, some triterpene saponins co-eluted. Specifically, this was observed for compounds **1** and **2**, **3** and **4**, **17** and **18** and **6**, **7**, **8** and **9**. In all cases, this chromatographic behavior can be explained by the similarities in the structures of their molecules (Figure 2).

Thus, the patterns of the NADES extraction activities, in respect to individual triterpene saponins, differed essentially (Figure 2), which can mostly be explained by the differences in analyte structures. Moreover, isomerism also impacts this phenomenon. For example, the recovery patterns of two isomers of calendulaglycosides C (Figure 2b,f) clearly differed from each other. It is also apparent that different types of NADES have different affinities for triterpene saponins of *A. elata.* Obviously, the structures of the individual constituents of NADES extracts, their ratio and their solvation degree directly affect the interactions between the molecules of solvents and target analytes [50,51].

In general, the efficiencies of NADES in the extraction of triterpene saponins from *A. elata* roots increased in the following order: water-free quaternary ammonium salt–dicarboxylic acid mixture (ND1) > 20% (*v*/*v)* aq. sugar–dicarboxylic acid (ND7) > 10% (*v*/*v)* aq. sugar–dicarboxylic acid (ND6) > 30% (*v*/*v)* aq. quaternary ammonium salt–monocarboxylic acid (ND4). Most likely, the observed high recoveries of triterpene saponins by NADES can be explained by close pH values of the NADES mixtures (2.26 for ND1 and 3.84 for ND4) to the pKa of oleanolic acid (4.74), the aglycon of all detected triterpene saponins.

Due to the fact that all of these mentioned above—ND3, 4, 6 and 7—showed much less viscosity in comparison to ND1 and ND5; the mixtures ND1, ND3 and ND7 were characterized with better mass transfer for triterpene natural products, which was most clearly seen for the mixtures based on malic acid or lactic acid. On the other hand, the solvent ND5 (based on the mixture of choline chloride and oxalic acid) showed weak extraction efficiency (Figure 2). Our results were in agreement with the studies of Lanjekar and coworkers, who demonstrated that choline chloride–lactic acid mixture (1:1 *v*/*v*) was efficient for the extraction of triterpene saponin glycyrrhizic acid from Glycyrrhiza glabra [41,52]. Furthermore, Suresh et al., 2022 showed that this NADES was also efficient in the extraction of steroidal saponins from *Trillium govanianum* [53]. Choline chloride–lactic acid mixture (1:2 *v*/*v*) proved to be efficient in the extraction of saponins from *Acanthopanax senticosus* [54]. Similar data for lactic acid-based NADES were obtained by Liu et al., 2023 for steroidal saponins from *Polygonatum cyrtonema* [55]. Increasing the relative contents of the aqueous component in NADES leads to better solvation of the polar analytes and, therefore, improved extractive capacity. The addition of water reduced viscosity. It promoted diffusion, while hydrogen bonds between the components weakened it [56]. It is noteworthy that for the extraction of kalopanax–Saponin F isomer 2 (**6**), calendulaglycoside C isomer 1 (**7**), calendulaglycoside C isomer 2 (**13**), araloside A isomer 1 (**14**), guaiacin B isomer 2 (**15**), oleanolic acid-3-*O*-(hexosyl)-28-1-hexouronide ester isomer 2 (**18**) and oleanolic acid-3-*O*-hexuronide-(1-3-pentafuranoside) (**19**), the most appropriate solvent was ND1 (choline chloride and malic acid in the ratio of 1:1). This solvent was also reported to be efficient for the extraction of ginsenoside Rb1 from *Panax ginseng* stems [57] and steroidal saponins from *Dioscoreae nipponicae* [40]. Other researchers remarked upon the efficiency of the acetylcholine chloride–malic acid–water mixture (ratio 1:2:2 *v*/*v*/*v*) for the extraction of triterpene saponins–madecassoside and asiaticoside from *Centella asiatica* [58].

It is important to note that the application of NADES not only gives access to a better efficiency of extraction, but also provides improved stabilities of the resulting extracts. On the other hand, the aqueous extracts are prone to microbial contamination and therefore have limited shelf life [24,34]. This might be underlined by the presence of additional hydroxyl and carboxyl groups in their structure of NADES, which are readily involved in the formation of hydrogen bonds with triterpene saponins [23,59].

## 4. Materials and Methods

### 4.1. Chemicals

Conventional solvents (purified water, ethanol 96%) were used for the comparison of efficiency of extraction. Unless stated otherwise, the materials were obtained from the following manufacturers: InnoGreenChem B.V. (Nijmegen, The Netherlands): lactic acid (80%); Merck KGaA (Darmstadt, Germany): acetonitrile (LC-MS grade), ammonium formate (99%, LC-MS grade), formic acid (98%, LC-MS grade), methanol (LC-MS grade) and water (LS-MS grade); NevaReactive (Saint Petersburg, Russia): choline chloride (≥99%) and oxalic acid (≥99%); sorbitol (≥99%) and malic acid (≥99%). Water was purified in-house with a water conditioning and purification system: GenPure Pro UV-TOC system (resistance 18 mΩ/cm, Thermo Fisher Scientific, Langenselbold, Germany).

### 4.2. Plant Material and Extraction Procedures

The roots of *A. elata* were collected in the Far East (Khabarovsk region) of Russia and the species identity was confirmed in the Department of Pharmacognosy of the Saint Petersburg State Chemical Pharmaceutical University (voucher of specimens MW0107420). All collected samples were dried without direct irradiation at room temperature. Whole roots (Y), isolated root bark (X) and root core (Z) were extracted using water, ethanol and different NADES. About 1.0 g of dried raw material (X, Y and Z) was extracted with 100 mL of hot boiling water with reflux for 2 h. Ethanol extracts were obtained by the extraction of 1.0 g of each part of raw material (X, Y and Z) with 100 mL of 96% (*v*/*v*) EtOH in Soxhlet. The resulting extracts were filtered, concentrated in a vacuum and freeze dried. Further, 10.0 mg of each lyophlisate was dissolved in 1 mL of purified water and centrifuged for 10 min at 10,000 rpm (4 °C), and the supernatant (180 µL) was analyzed by RP-UHPLC-QqTOF-MS.

All NADES were prepared by the heating and stirring method [23]. In detail, the mixtures of hydrogen bond donor and hydrogen bond acceptor with a certain amount of water (Table 2) were slightly heated (at the temperatures not exceeding 80 °C) with continuous gentle agitation before forming a clear transparent liquid (about 120 min) [23]. The composition of NADES (i.e., the composing solvents and their ratios) relied on the literature data [23,24] and our previous experiment.

Extraction using NADES was performed by maceration with continuous stirring. A total of 1.0 g of dried raw material and 40.0 g of NADES were transferred in flask. The mixture was stirred at 300 rpm for 60 min at 35 °C. The residue was filtered. Prior to metabolic profiling, NADES extracts were dissolved in water (in a ratio of 2:3) and placed in an ultrasonic bath for 15 min (35 kHz): 200 µL was centrifuged for 10 min at 10,000 rpm (4 °C). The supernatant (180 µL) was further analyzed using RP-UHPLC-QqTOF-MS.

### 4.3. Metabolite Analysis

The samples were analyzed using the randomization/standardization strategy by reversed phase–ultra-high-performance liquid chromatography–electrospray ionization–quadrupole-time-of-flight mass spectrometry (RP-UHPLC-ESI-QqTOF-MS) using a liquid chromatograph–mass spectrometer SHIMADZU LCMS-9030 System (Shimadzu Corporation, Kyoto, Japan) operated in negative ion mode. The data acquisition relied on the sequential window acquisition of all theoretical mass spectra (SWATH) mode. The chromatographic and mass spectrometric settings are specified in Supplementary Information S1 (Table S5). For the interpretation of the LC-MS data, Shimadzu LabSolution (Shimadzu Corporation, Kyoto, Japan), MSDial (version 3.12, free available via http://prime.psc.riken.jp/Metabolomics_Software/MS-DIAL/index2.html (accessed on 3 March 2023)), and manual mass spectra interpretation were used. The quantitation relied on the integration of the corresponding extracted ion chromatograms (XICs, *m*/*z* ± 0.025) at specific retention times (t_R_). The intensity matrix was generated after data processing in MSDial. Based on the intensities, the fold changes were calculated by the comparison of the amounts in the aqueous and ethanolic extracts. Data visualization was performed using Microsoft Excel 2016 (Supplementary Information S2 (Figure S17)).

For all features, which were annotated based on their [M-H]^−^ ions observed in the MS spectra (MS1 scans) of triterpene glycoside structures with a mass accuracy of better than 10 ppm but did not yield unambiguously interpretable fragmentation patterns in the first SWATH mode experiments (typically due to the uncertainty of the fragmentation patterns and simultaneous fragmentation of two or more intense *m*/*z*), additional SWATH and data-dependent acquisition (DDA) RP-UHPLC-MS/MS experiments were conducted using a TripleTOF 6600 mass spectrometer (AB Sciex, Darmstadt, Germany), using the LC conditions summarized in Supplementary Information S1 (Table S6). The nebulizer (GS1), drying (GS2) and curtain (CUR) gases were set to 60, 70 and 55 psig, while the ion spray voltage was set to −4500 V. The MS experiments were accomplished in the TOF-scan mode (the accumulation time was 50 or 75 ms in SWATH and DDA experiments, respectively) in the *m*/*z* range of 65–1250. The MS/MS conditions in the SWATH method were set as follows: each analysis was performed with 60 ms of accumulation time at a collision energy of −45 V with a collision energy spread (CES) of 35 V and a declustering potential (DP) of −35 V. The MS/MS conditions in the IDA method were set as follows: each analysis was performed with an accumulation of time 175 ms and a range of collision energy of −45 V with a collision energy spread (CES) of 35 V and a declustering potential (DP) of −35 V. Nitrogen was used as the CAD gas.

## 5. Conclusions

In this study, a total of twenty triterpene saponins (derivatives of oleanolic acid) were identified in the roots, root bark and root core of *A. elata* by RP-UHPLC-ESI-QqTOF-MS. Thereby, compounds **1**, **3**, **4**–**7**, **13**, **15** and **20** were identified in the roots of *A. elata* for, to the best of our knowledge, the first time. Furthermore, seven acid-based NADES were successfully applied for the extraction of triterpene saponins from the roots of *A. elated* for the first time. All of the tested NADES were able to extract triterpene saponins. The maximal number of triterpene saponins was identified using the ND1 (choline chloride with malic acid: 1:1) and ND3 (choline chloride with lactic acid: 1:3) extracts. Remarkably, NADES proved to be more efficient solvents than water or ethanol for the extraction of thirteen triterpene saponins. The relative recoveries of the compounds **5**–**10**, **12**–**16**, **18** and **19** were higher using NADES than when using water or ethanol. For the extraction of compounds **6**–**10**, **12**, **15**, **16**, **18** and **19**, the addressed NADES appeared to be more efficient than conventional solvents. Our data open the prospect of replacing aqueous and alcohols with non-toxic, “green” NADES for the extraction of *A. elata*.

## Figures and Tables

**Table 1:** Targeted metabolites annotated in different parts of the root of *A. elata* var. *mandshurica* (Rupr. & Maxim.) J. Wen extracted using conventional and natural deep eutectic solvents by reversed phase–ultra-high-performance liquid chromatography–mass spectrometry in (RP-UHPLC-QqTOF-MS) in the negative ion mode.

No.	t_R_ (min)	*m*/*z* [M−H]^−^ Observed	*m*/*z* [M−H]^−^ Calculated	Elemental Composition [M−H]^−^	MS2 Fragmentation Patterns-Product Ions, *m*/*z* (Rel. Intensity)	Δm (ppm)	Assignment	Plant Part	Solvent	Ref.	Suppl. Spectra
**1**	3.8	911.4993	911.5010	C_47_H_75_O_17_^−^	455.3507 (20), 617.4041 (15), 749.4472 (15), 911.4993 (100)	1.9	Guaiacin B isomer 1	X,Y,Z ^1^	W,E,ND1,2,3,4,6,7 ^2^	[44]	Figure S1
**2**	3.8	1235.6107	1235.6066	C_59_H_95_O_27_^−^	455.3522 (50), 617.4059 (15), 749.4492 (100), 911.5035 (10), 1235.6107 (10)	−3.3	Oleanolic acid-3-*O*- (triglucopyranosyl-1-3- arabinopyranosyl)-28-1- glucopyranosyl	X	W,E,ND1,2,3,4		Figure S2
								Y	W,E,ND1,2,3,4,6,7		
								Z	W,E,ND1,3,4,6,7		
**3**	3.9	1087.5317	1087.5331	C_53_H_83_O_23_^−^	455.3509 (10), 701.4265 (5), 925.4814 (15), 1087.5317 (100)	1.3	Kalopanax-Saponin F isomer 1	X	W,E, ND1,2,3,4,6,7	[45]	Figure S3
								Y	All		
								Z	W,E,ND1,3,4,6,7		
**4**	3.9	1117.5390	1117.5436	C_54_H_85_O_24_^−^	455.3499 (3), 731.4347 (5), 955.4898 (10), 1117.5390 (100)	4.1	Calendulaglycoside A	X,Z	W,E,ND1,2,3,4,6,7	[46]	Figure S4
								Y	All		
**5**	4.0	1249.5869	1249.5859	C_59_H_93_O_28_^−^	455.3487 (5), 701.4254 (15), 925.4743 (7), 1057.5219 (80), 1087.5337 (50), 1153.5538 (100), 1249.5869 (70)	−0.8	Araliaarmoside	X,Y,Z	W,E,ND1,2,3,4,6,7	[47]	Figure S5
**6**	4.3	1087.5336	1087.5331	C_53_H_83_O_23_^−^	455.3509 (10), 701.4265 (5), 925.4814 (15), 1087.55336 (100)	−0.5	Kalopanax-Saponin F isomer 2	X,Z	W,E,ND1,2,3,4,6,7	[45]	Figure S3
								Y	All		
**7**	4.3	955.4859	955.4908	C_48_H_75_O_19_^−^	455.3511 (5), 569.379 (5), 793.4317 (15), 955.4859 (100)	5.1	Calendulaglycoside C isomer 1	X,Y,Z	W,E,ND1,2,3,4,6,7	[46]	Figure S6
**8**	4.3	1073.5569	1073.5538	C_53_H_85_O_22_^−^	455.3518 (50), 617.4052 (20), 749.4485 (30), 911.5022 (100), 1073.5569 (15)	−2.9	Oleanolic acid-3-*O*- (diglucopyranosyl-1-3- arabinopyranosyl)-28-1- glucopyranosyl ester	X	E,ND1,2,3,4,6,7		Figure S7
								Y	E,ND1,2,3,6,7		
								Z	ND1,3,6,7		
**9**	4.3	1119.5606	1119.5616	C_54_H_87_O_24_^−^	455.3521 (5), 617.4057 (5), 749.4490 (7), 911.5019 (100), 1073.5551 (15), 1119.5606 (13)	0.9	Oleanolic acid-3-*O*- (methyldioxy-trihexopyranosyl-1-3- pentopyranosyl)-28-1- hexopyranosyl ester	X,Y	W,E,ND1,2,3,4,6,7		Figure S8
								Z	W,E,ND1,3,4,6,7		
**10**	4.4	1057.5249	1057.5225	C_52_H_81_O_22_^−^	455.3517 (5), 701.4286 (7), 763.4309 (5), 895.4698 (7), 1057.5249 (100)	−2.3	Araloside B	X,Y,Z	W,E,ND1,2,3,4,6,7	[48]	Figure S9
**11**	4.5	1089.5493	1089.5487	C_53_H_85_O_23_^−^	455.3501 (5), 719.4360 (10), 881.4901 (100), 1043.5437 (12), 1089.5493 (12)	−0.6	Araliasaponin III	X,Z	W,E,ND1,2,3,4,6,7	[49]	Figure S10
								Y	W,E,ND1,3,4,6,7		
**12**	4.6	793.4312	793.4380	C_42_H_65_O_14_^−^	455.3471 (10), 631.3785 (15), 793.4312 (100)	8.6	Oleanolic acid-3-*O*- (hexosyl)-28-1-hexouronide ester isomer 1	X,Z	W,E,ND1,2,3,4,6,7		Figure S11
								Y	All		
**13**	5.1	955.4899	955.4908	C_48_H_75_O_19_^−^	455.3511 (5), 569.379 (5), 793.4317 (15), 955.4899 (100)	0.9	Calendulaglycoside C isomer 2	X,Y,Z	W,E,ND1,2,3,4,6,7	[46]	Figure S6
**14**	5.3	925.4805	925.4802	C_47_H_73_O_18_^−^	455.3508 (2), 569.3831 (5), 731.4366 (20), 925.4803 (100)	0.3	Araloside A isomer 1	X,Z	W,E,ND1,2,3,4,6,7	[48]	Figure S12
								Y	All		
**15**	5.4	911.5016	911.5010	C_47_H_75_O_17_^−^	455.3507 (20), 617.4041 (15), 749.4472 (15), 911.5016 (100)	−0.7	Guaiacin B isomer 2	X,Z	W,E,ND1,2,3,4,6,7	[44]	Figure S1
								Y	All		
**16**	5.5	925.4807	925.4802	C_47_H_73_O_18_^−^	455.3508 (2), 569.3831 (5), 731.4366 (20), 925.4803 (100)	0.5	Araloside A isomer 2	X,Z	W,E,ND1,2,3,4,6,7	[48]	Figure S12
								Y	All		
**17**	5.6	895.4696	-	-	455.3508 (5), 551.3731 (5), 895.4696 (100)		Oleanolic acid unknown derivatives	X,Y,Z	W,E,ND1,2,3,4,6,7		Figure S13
**18**	5.6	793.4360	793.4380	C_42_H_65_O_14_^−^	455.3471 (10), 631.3785 (15), 793.4312 (100)	2.5	Oleanolic acid-3-*O*- (hexosyl)-28-1-hexouronide ester isomer 2	X,Z	W,E,ND1,2,3,4,6,7		Figure S11
								Y	All		
**19**	5.7	763.4260	763.4274	C_41_H_63_O_13_^−^	455.3502 (3), 631.3822 (5), 763.4260 (100)	1.8	Oleanolic acid 3-*O*- hexuronide-(1-3- pentafuranoside)	X,Z	W,E,ND1,2,3,4,6,7		Figure S14
								Y	All		
**20**	5.8	911.4949	911.5010	C_47_H_75_O_17_^−^	455.3507 (20), 617.4041 (15), 749.4472 (15), 911.4949 (100)	6.7	Guaiacin B isomer 3	X,Y,Z	W,E,ND1,2,3,4,6,7	[44]	Figure S1

^1^ X—root bark. Y—whole root. Z—core of root. ^2^ W—water. E—ethanol. ND1—NADES with choline chloride and malic acid (molar ratio 1:1). ND2—NADES with the molar ratio of choline chloride and malic acid of 1:2. ND3—NADES with the molar ratio of choline chloride and lactic acid of 1:3. ND4—NADES with the molar ratio of choline chloride and lactic acid of 1:3 + 30% (*v*/*v*) water. ND6—NADES with the molar ratio of sorbitol and malic acid of 1:1 + 10% (*v*/*v*) water. ND7—NADES with the molar ratio of sorbitol and malic acid of 1:2 + 20% (*v*/*v*) water. All—metabolites were found in all extracts. The annotated metabolites (listed in Table 1) were quantified on the relative basis in the aqueous and ethanolic extracts of *A. elata* whole roots, bark and core. For this purpose, extracted ion chromatograms (XICs, *m*/*z* ± 0.02) were built for their [M-H]^−^ signals and integrated at characteristic t_R_s. The primary results of the bioinformatic analysis and peak integration are summarized in Supplementary Information S2 (Tables S7–S9). The analysis revealed the compounds **1**, **2**, **6** and **15** as being the most abundant in root bark, while metabolites **2**–**5**, **7**–**12**, **14**, **16**–**19** and **20** dominated in the core of roots.

**Table 2:** Composition of NADES [23,24].

Code	Component 1	Component 2	Molar Ratio	Amount of Water (% (*v*/*v*))
ND1	Choline chloride	Malic acid	1:1	-
ND2	Choline chloride	Malic acid	1:2	-
ND3	Choline chloride	Lactic acid	1:3	-
ND4	Choline chloride	Lactic acid	1:3	30
ND5	Choline chloride	Oxalic acid	1:1	15
ND6	Sorbitol	Malic acid	1:1	10
ND7	Sorbitol	Malic acid	1:2	20

## Data Availability

The data presented in this study are available on request from the corresponding authors.

## Supplementary Materials

The following supporting information can be downloaded at: https://www.mdpi.com/article/10.3390/molecules28083614/s1. Supplementary Information S1—Table S1: the list of triterpene saponins predicted in different parts of *A. elata* based on literature mining. Table S2: triterpene saponins annotated in the extracts of *A. elata* roots by RP-HPLC-QqTOF-MS in untargeted SWATH experiments. Table S3: list of compounds confirmed by MS2 analysis in the roots of *Aralia elata*. Table S4: triterpene saponins annotated in the extracts of *A. elata* roots by RP-UHPLC-QqTOF-MS and MS/MS in SWATH and DDA experiments. Table S5: the conditions of RP-HPLC separation and the settings ESI-QqTOF-MS applied for the profiling of *A. elata* root semi-polar secondary metabolites. Table S6: the conditions of RP-UHPLC separation and the settings for ESI-QqTOF-MS applied for the SWATH and DDA MS/MS analysis of *A. elata* root semi-polar secondary metabolites. Figures—Figures S1–S14: spectral data of triterpene saponins annotated in the extracts of *A. elata* roots. Figure S15: full t_R_ range in the chromatograms of ND1 and ND3 extracts of the whole roots of *A. elata*. Figure S16: structures and relative recoveries of 14 (araloside isomer 1, A) and 18 (oleanolic acid-3-*O*-(hexosyl)-28-1-hexouronide ester isomer 1, B), expressed as the difference (fold) in comparison to those observed in aqueous and ethanolic extracts. Supplementary Information S2—Table S7: results of untargeted metabolomic analysis of *A. elata* with MSDial software. Table S8: relative recoveries of triterpene saponins annotated in *A. elata* roots, extracted by conventional solvents and different types of NADES. Table S9: relative recoveries of individual triterpene saponins in aqueous, ethanolic and NADES extracts of *A. elata* whole roots, root core and bark. Figure S17: relative recovery diagrams of the triterpene saponins annotated in *A. elata* roots extracted with conventional solvents and different types of NADES. References [2,3,19,20,44,45,46,47,48,49,60,61,62,63,64,65,66,67,68,69,70,71,72,73,74,75,76,77,78,79,80,81,82,83,84,85,86,87] are cited in the supplementary materials.
