# Supplementary material for: Natural Deep Eutectic Solvents for the Extraction of Triterpene Saponins from Aralia elata var. mandshurica (Rupr. & Maxim.) J. Wen
Source: Molecules. 2023 Apr 21;28(8):3614. doi: 10.3390/molecules28083614 (PMC10140851; doi:10.3390/molecules28083614)
Supplement: Supplementary file 1 [file molecules-28-03614-s001.zip › molecules-2292933-Supplementary Information S1.pdf]

Natural deep eutectic solvents for the extraction of triterpenoid saponins from *Aralia elata* var. *mandshurica* (Rupr. & Maxim.) J. Wen

Alyona A. Petrochenko[1]‡, Anastasia Orlova‡[2], Nadezhda Frolova[3], Evgeny B. Serebryakov[4], Alena Soboleva[2], Elena V. Flisyuk[1], Andrej Frolov[2]* and Alexander N. Shikov[1]*

Supplementary Information S1

[1]Department of Technology of Pharmaceutical Formulations, St. Petersburg State Chemical Pharmaceutical University, 197376 Saint-Petersburg, Russia, [2]K.A. Timiryazev Institute of Plant Physiology RAS, 127276 Moscow, Russia, [3]Department of Plant Physiology and Biochemistry, St. Petersburg State University, 199034 Saint-Petersburg, Russia, [4]Chemical Analysis and Materials Research Centre, St. Petersburg State University, 198504 Saint-Petersburg, Russia

‡These authors contribute equally to the manuscript

*Corresponding authors:

Dr. Andrej Frolov
K.A. Timiryazev Institute of Plant Physiology RAS
Laboratory of analytical biochemistry and biotechnology
Moscow, Russia
Tel. +7 499 678-54-00
Email: frolov@ifr.moscow

Prof. Alexander N. Shikov
St. Petersburg State Chemical Pharmaceutical University
Department of Technology of Pharmaceutical Formulations
Saint-Petersburg, Russia
Tel. +7 911 701-01-06
Email: alexs79@mail.ru

Directory

Table S1 The list of triterpene saponins predicted in different parts of *Aralia elata var. mandshurica* (*Rupr. & Maxim.*) J. Wen based on literature mining 5

Table S2 Triterpene saponins annotated in the extracts of *Aralia elata var. mandshurica* (*Rupr. & Maxim.*) J. Wen roots by reversed ultra phase high performance liquid chromatography-mass spectrometry (RP-UHPLC-QqTOF-MS) in untargeted SWATH experiments (with the inclusion list containing the *m/z* of all predicted $[M-H]^-$ ions using published data)...9

Table S3 List of compounds confirmed by MS^2 analysis in the roots of *Aralia elata var. mandshurica* (*Rupr. & Maxim.*) J. Wen 11

Table S4 Triterpene saponins annotated in the extracts of *Aralia elata var. mandshurica* (*Rupr. & Maxim.*) J. Wen roots by reversed phase ultra-high performance liquid chromatography-mass spectrometry and tandem mass spectrometry (RP-UHPLC-QqTOF-MS and MS/MS) in SWATH and data-dependent acquisition (DDA) experiments 12

Table S5 The conditions of reverse phase-ultra high performance liquid chromatographic (RP-UHPLC) separation and the settings for electrospray ionization-quadrupole-time of flight mass spectrometry (ESI-QqTOF-MS) applied for the profiling (untargeted analysis) of *Aralia elata var. mandshurica* (Rupr. & Maxim.) J. Wen root semi-polar secondary metabolites with QqTOF hybride mass spectrometer SHIMADZU LCMS-9030 System (SHIMADZU Corporation, Kyoto, Japan). ...14

Table S6 The conditions of reversed phase-ultrahigh performance liquid chromatographic (RP-UHPLC) separation and the settings for electrospray ionization-quadrupole-time of flight mass spectrometry (ESI-QqTOF-MS) applied for the SWATH and DDA MS/MS analysis of *Aralia elata var. mandshurica* (Rupr. & Maxim.) J. Wen root semi-polar secondary metabolites with Waters ACQUITY UPLC I-Class UPLC System (Waters GmbH, Eschborn, Germany) coupled online to a hybrid quadrupole-time of flight mass spectrometer (QqTOF-MS) AB Sciex TripleTOF 6600 (AB Sciex, Darmstadt, Germany). 17

Figures S1–S14 Chromatographic and mass spectral data of triterpene saponins annotated in the extracts of Aralia elata var. mandshurica (Rupr. & Maxim.) J. Wen roots by reversed phase ultra-high performance liquid chromatography-mass spectrometry and tandem mass spectrometry (RP-UHPLC-QqTOF-MS and MS/MS) in SWATH and data-dependent acquisition (DDA) experiments 21

Figure S1. Extracted ion chromatogram (XIC) of *m/z* 911.5000 ± 0.02 (A), the MS spectra (B) and MS/MS fragmentation patterns (C) of the compounds 1, 15, 20 annotated in the total ethanolic extract of *Aralia elata var. mandshurica* (*Rupr. & Maxim.*) J. Wen roots as guaiacin B isomers at t_R 7.77, 10.98 and 11.01, respectively. 23

Figure S2. Extracted ion chromatogram *m/z* 1235.6100 ± 0.02 (A), the MS spectra (B) and MS/MS fragmentation patterns (C) of the compounds 2 annotated in the total ethanolic extract of *Aralia elata var. mandshurica* (*Rupr. & Maxim.*) J. Wen roots as oleanolic acid-3-*O*-(triglucopyranosyl-1-3-arabinopyranosyl)-28-1-glucopyranosyl. 25

Figure S3. Extracted ion chromatogram *m/z* 1087.5400 ± 0.02 (A), the MS spectra (B) and MS/MS fragmentation patterns (C) of the compounds 3, 6 annotated in the total ethanolic extract of *Aralia elata var. mandshurica* (*Rupr. & Maxim.*) J. Wen roots as kalopanax-saponin F isomers at t_R 7.89 and 8.41, respectively. 28

Figure S4. Extracted ion chromatogram m/z 1117.5400 ± 0.02 (A), the MS spectra (B) and MS/MS fragmentation patterns (C) of the compounds 4 annotated in the total ethanolic extract of Aralia elata var. mandshurica (Rupr. & Maxim.) J. Wen roots as calendulaglycoside A. 30

Figure S5. Extracted ion chromatogram *m/z* 1249.5900 ± 0.02 (A), the MS spectra (B) and MS/MS fragmentation patterns (C) of the compounds 5 annotated in the total ethanolic extract of *Aralia elata var. mandshurica* (*Rupr. & Maxim.*) J. Wen roots as araliaarmoside. 32

Figure S6. Extracted ion chromatogram *m/z* 955.4900 ± 0.02 (A), the MS spectra (B) and MS/MS fragmentation patterns (C) of the compounds 7, 13 annotated in the total ethanolic extract of *Aralia elata var. mandshurica* (*Rupr. & Maxim.*) J. Wen roots as calendulaglycoside C isomers at t_R 8.59 and 10.07, respectively.. 35

Figure S7. Extracted ion chromatogram m/z 1073.5600 ± 0.02 (A), the MS spectra (B) and MS/MS fragmentation patterns (C) of the compounds 8 annotated in the total ethanolic extract of *Aralia elata var. mandshurica* (*Rupr. & Maxim.*) J. Wen roots as oleanolic acid-3-*O*-(diglucopyranosyl-1-3-arabinopyranosyl)-28-1-glucopyranosyl ester. 37

Figure S8. Extracted ion chromatogram m/z 1119.5600 ± 0.02 (A), the MS spectra (B) and MS/MS fragmentation patterns (C) of the compounds 9 annotated in the total ethanolic extract of *Aralia elata var. mandshurica* (*Rupr. & Maxim.*) J. Wen roots as oleanolic acid-3-*O*-(methyldioxy-trihexopyranosyl-1-3-pentopyranosyl)-28-1-hexopyranosyl ester.. 39

Figure S9. Extracted ion chromatogram m/z 1057.5300 ± 0.02 (A), the MS spectra (B) and MS/MS fragmentation patterns (C) of the compounds 10 annotated in the total ethanolic extract of *Aralia elata var. mandshurica* (*Rupr. & Maxim.*) J. Wen roots as araloside B. 41

Figures S10. Extracted ion chromatogram m/z 1089.5500 ± 0.02 (A), the MS spectra (B) and MS/MS fragmentation patterns (C) of the compounds 11 annotated in the total ethanolic extract of *Aralia elata var. mandshurica* (*Rupr. & Maxim.*) J. Wen roots as araliasaponin III 43

Figure S11. Extracted ion chromatogram m/z 793.4400 ± 0.02 (A), the MS spectra (B) and MS/MS fragmentation patterns (C) of the compounds 12, 18 annotated in the total ethanolic extract of *Aralia elata var. mandshurica* (*Rupr. & Maxim.*) J. Wen roots as oleanolic acid-3-*O*-(hexosyl)-28-1-hexouronide ester isomers t_R 9.02 and 10.84, respectively.46

Figure S12. Extracted ion chromatogram m/z 925.4800 ± 0.02 (A), the MS spectra (B) and MS/MS fragmentation patterns (C) of the compounds 14, 16 annotated in the total ethanolic extract of *Aralia elata var. mandshurica* (*Rupr. & Maxim.*) J. Wen roots as araloside A isomers t_R 10.266 and 10.401, respectively 49

Figure S13. Extracted ion chromatogram m/z 895.4500 ± 0.02 (A), the MS spectra (B) and MS/MS fragmentation patterns (C) of the compounds 17 annotated in the total ethanolic extract of *Aralia elata var. mandshurica* (*Rupr. & Maxim.*) J. Wen roots as oleanolic acid unknown derivatives. 51

Figure S14. Extracted ion chromatogram m/z 763.4300 ± 0.02 (A), the MS spectra (B) and MS/MS fragmentation patterns (C) of the compounds 19 annotated in the total ethanolic extract of *Aralia elata var. mandshurica* (*Rupr. & Maxim.*) J. Wen roots as oleanolic acid 3-*O*-hexuronide-(1-3-pentafuranoside). 53

Figure S15. The full t_R range obtained in the chromatograms of ND1 and ND3 extracts of whole roots of *A. elata* (peak numbers correspond to compounds listed in Table 1). 54

Figure S16. Structures and relative recoveries of 14 (araloside isomer 1 (a)), 18 (oleanolic acid-3-*O*-(hexosyl)-28-1-hexouronide ester isomer 1 (b)), *13*, expressed as the difference (fold) in comparison to those observed in aqueous and ethanolic extracts. Fold changes comparison with water (blue), fold changes comparison with ethanol (orange), * (for recoveries in relation to water) or ** (for recoveries in relation to ethanol) - $p \leq 0.05$ vs. control. The compounds are numbered as in Table 1. ND1 – NADES with choline chloride and malic acid (molar ratio 1:1), ND2 – NADES with the molar ratio of choline chloride and malic acid of 1:2, ND3 – NADES with the molar ratio of choline chloride and lactic acid of 1:3, ND4 - NADES with the molar ratio of choline chloride and lactic acid of 1:3+ 30%

(*v/v*) water, ND6 – NADES with the molar ratio of sorbitol and malic acid of 1:1 + 10% (*v/v*) water, ND7 - NADES with the molar ratio of sorbitol and malic acid of 1:2+20% (*v/v*) water. 55

References..56

Tables

Table S1. The list of triterpene saponins predicted in different parts of *Aralia elata var. mandshurica* (*Rupr. & Maxim.*) J. Wen based on literature mining

Compound Name	Elemental composition	Molar mass	Theoretical *m/z*	Ref
7-*O*-α-rhamnopyranosyl-(S)-quercetin-3-*O*-β-6-caffeoyl-glucopyranosyl-(1→2)-α-rhamnopyranoside	$C_{42}H_{46}O_{23}$	918.8134	917.8061	[1]
7-*O*-α-rhamnopyranosyl-(S)-quercetin-3-*O*-β-glucopyranosyl-(1→2)-α-rhamnopyranoside	$C_{33}H_{40}O_{20}$	756.6686	755.6613	[1]
Acanthopanaxoside E	$C_{42}H_{66}O_{15}$	810.4402	809.4329	[2]
Acanthoside D	$C_{48}H_{78}O_{18}$	942.5188	941.5115	[3]
Acutoside A	$C_{42}H_{68}O_{13}$	780.4660	779.4587	[4]
Anchusosid 2	$C_{48}H_{78}O_{18}$	942.5188	941.5115	[4]
Aradecoside D	$C_{59}H_{96}O_{27}$	1237.395	1236.3877	[5]
Araliasaponin I	$C_{47}H_{76}O_{18}$	928.5032	927.4959	[6]
Araliasaponin II	$C_{47}H_{76}O_{19}$	944.4981	943.4908	[6]
Araliasaponin III	$C_{53}H_{86}O_{23}$	1090.5559	1089.5486	[6]
Araliasaponin IV	$C_{54}H_{88}O_{23}$	1104.5716	1103.5643	[6]
Araliasaponin XVII	$C_{47}H_{74}O_{18}$	926.4875	925.4802	[7]
Araliaarmoside	$C_{59}H_{94}O_{28}$	1250.5932	1249.5859	[8]
Araliachinosides I	$C_{36}H_{56}O_{8}$	616.3975	615.3902	[9]
Araliasaponin IV	$C_{54}H_{88}O_{24}$	1120.5666	1119.5593	[10]
Araliasaponin IX	$C_{53}H_{86}O_{32}$	1090.5560	1089.5487	[6]
Araliasaponin V	$C_{54}H_{88}O_{23}$	1104.5716	1103.5643	[6]
Araliasaponin VI	$C_{54}H_{88}O_{24}$	1120.5666	1119.5593	[6]
Araliasaponin VII	$C_{54}H_{88}O_{24}$	1120.5666	1119.5593	[6]
Araliasaponin VIII	$C_{60}H_{98}O_{30}$	1298.6143	1297.6070	[6]
Araloside A	$C_{47}H_{74}O_{18}$	926.4875	925.4802	[11]
Araloside A methyl ester	$C_{48}H_{76}O_{18}$	940.5032	939.4959	[3]
Araloside B	$C_{52}H_{82}O_{22}$	1058.5298	1057.5225	[11]

Araloside C	$C_{53}H_{84}O_{23}$	1088.5403	1087.5330	[3]
Araloside D	$C_{46}H_{74}O_{16}$	882.4977	881.4904	[12]
Araloside G	$C_{54}H_{88}O_{23}$	1104.5716	1103.5643	[3]
Araloside H	$C_{47}H_{74}O_{18}$	926.4875	925.4802	[13]
Araloside J	$C_{47}H_{74}O_{18}$	926.4875	925.4802	[13]
Armatoside A	$C_{47}H_{74}O_{18}$	926.4875	925.4802	[14]
Armatoside B	$C_{47}H_{74}O_{19}$	942.4824	941.4751	[14]
Calendulaglycoside A	$C_{54}H_{86}O_{24}$	1118.5509	1117.5436	[15]
Calendulaglycoside C	$C_{48}H_{76}O_{19}$	956.4980	955.4907	[15]
Calenduloside B	$C_{42}H_{68}O_{13}$	780.4660	779.4587	[16]
Calenduloside E	$C_{36}H_{56}O9$	632.3924	631.3851	[16]
Calenduloside G	$C_{41}H_{64}O_{13}$	764.4347	763.4274	[16]
Chikusetsusaponin (1)	$C_{36}H_{58}O8$	618.4131	617.4058	[3]
Chikusetsusaponin IV	$C_{47}H_{74}O_{18}$	926.4875	925.4802	[3]
Chikusetsusaponin Ib	$C_{41}H_{64}O_{13}$	764.4347	763.4274	[17]
Chikusetsusaponin IVa	$C_{42}H_{66}O_{14}$	808.4609	807.4536	[18]
Chikusetsusaponin V	$C_{48}H_{76}O_{19}$	956.4981	955.4908	[18]
Collinsonidin	$C_{41}H_{66}O_{13}$	766.4503	765.4430	[4]
Congmunoside IX	$C_{53}H_{86}O_{23}$	1088.5403	1087.5330	[19]
Congmunoside VIII	$C_{60}H_{98}O_{30}$	1298.6143	1297.6070	[2]
Congmunoside X	$C_{60}H_{98}O_{28}$	1266.6245	1265.6172	[20]
Congmunoside XV	$C_{54}H_{88}O_{24}$	1120.5666	1119.5593	[21]
Congmuyanoside A	$C_{41}H_{66}O_{14}$	782.4453	781.4380	[4]
Congmuyanoside B	$C_{48}H_{78}O_{19}$	958.5137	957.5064	[22]
Congmuyanoside D	$C_{42}H_{68}O_{14}$	764.4609	763.4536	[23]
Congmuyanoside E	$C_{48}H_{76}O_{19}$	956.4981	955.4908	[4]
Congmuyanoside G	$C_{60}H_{98}O_{29}$	1282.6193	1281.6120	[4]
Congmuyenoside A	$C_{41}H_{66}O_{13}$	766.4503	765.4430	[24]
Congmuyenoside III	$C_{60}H_{98}O_{29}$	1282.6193	1281.6120	[4]
Congmuyenoside IV	$C_{42}H_{68}O_{15}$	812.4558	811.4485	[23]
Durupcoside C	$C_{47}H_{76}O_{18}$	928.5032	927.4959	[25]
Echinocystic acid-3-*O*-β-*D*-glucopyranosyl-(1-3)- β-	$C_{46}H_{47}O_{15}$	839.2915	838.2842	[1]

D-glucuronopyranoside-6'-*O*-butyl ester				
Eclalbasaponin I	$C_{42}H_{68}O_{14}$	764.4609	763.4536	[23]
Eclalbasaponin III	$C_{48}H_{78}O_{19}$	958.5137	957.5064	[26]
Ecliptasaponin B	$C_{41}H_{66}O_{14}$	958.5137	957.5064	[27]
Elatoside A	$C_{47}H_{74}O_{18}$	926.4875	925.4802	[18]
Elatoside B	$C_{48}H_{76}O_{19}$	956.4981	955.4908	[18]
Elatoside D	$C_{54}H_{86}O_{24}$	1118.5509	1117.5436	[28]
Elatoside G	$C_{36}H_{56}O_{11}$	664.3824	663.3751	[29]
Elatoside H	$C_{42}H_{66}O_{15}$	810.4402	809.4329	[29]
Ginsenin R0	$C_{48}H_{76}O_{19}$	956.4981	955.4908	[18]
Guaiacin B	$C_{47}H_{76}O_{17}$	912.5083	911.5010	[30]
Hemsloside G2	$C_{55}H_{88}O_{24}$	1132.5666	1131.5593	[7]
Kalopanax-saponin F	$C_{53}H_{84}O_{23}$	1088.5403	1087.5330	[31]
Kalopanax-saponin F methyl ester	$C_{53}H_{86}O_{23}$	1088.5403	1087.5330	[31]
Lucynoside E	$C_{42}H_{68}O_{14}$	764.4609	763.4536	[23]
Lucynoside H	$C_{42}H_{68}O_{13}$	780.4660	779.4587	[4]
Momordin Ic	$C_{41}H_{64}O_{13}$	764.4347	763.4274	[32]
Olean-12-en-28-oic acid, 3-*O*-β-d-glucopyranosyl(1 → 4)-β-d-glucopyranosyl-olean acid	$C_{42}H_{68}O_{13}$	780.4660	779.4587	[23]
Pseudoginsenoside RT1	$C_{47}H_{74}O_{18}$	926.4875	925.4802	[33]
Pseudoginsenoside RT1 butyl ester	$C_{51}H_{82}O_{18}$	982.5501	981.5428	[33]
Quinoasaponin 2	$C_{48}H_{78}O_{19}$	958.5137	957.5064	[34]
Quinoasaponin B	$C_{47}H_{76}O_{18}$	928.5032	927.4959	[10]
Randianin	$C_{42}H_{68}O_{13}$	780.4660	779.4587	[4]
Salsoloside C	$C_{47}H_{74}O_{18}$	926.4875	925.4802	[35]
Silphioside A	$C_{43}H_{68}O_{14}$	808.4609	807.4536	[3]
Silphioside B	$C_{42}H_{68}O_{13}$	780.4660	779.4587	[23]
Silphioside E	$C_{48}H_{78}O_{18}$	942.5188	941.5115	[4]
Spinasaponin A	$C_{42}H_{66}O_{14}$	808.4609	807.4536	[36]
Spinasaponin A_{28}-*O*-glycoside	$C_{48}H_{76}O_{19}$	956.4981	955.4908	[36]
Stipuleanoside R1	$C_{47}H_{74}O_{18}$	926.4875	925.4802	[18]
Stipuleanoside R2	$C_{53}H_{84}O_{23}$	1088.5403	1087.5330	[28]
Taibaienoside I	$C_{51}H_{82}O_{18}$	982.5501	981.5428	[37]

Taibaienoside II	$C_{49}H_{78}O_{18}$	954.5188	953.5115	[37]
Taibaienoside III	$C_{55}H_{88}O_{23}$	1116.5716	1115.5643	[37]
Taibaienoside IV	$C_{46}H_{74}O_{14}$	850.5078	849.5005	[37]
Taibaienoside V	$C_{52}H_{86}O_{14}$	934.6018	933.5945	[37]
Taibaienoside VI	$C_{47}H_{74}O_{18}$	926.4875	925.4802	[37]
Taibaienoside VII	$C_{55}H_{88}O_{23}$	1116.5716	1115.5643	[37]
Taibaienoside VIII	$C_{57}H_{92}O_{23}$	1144.6029	1143.5956	[37]
Taibaienoside IX	$C_{47}H_{76}O_{17}$	912.5083	911.5010	[4]
Tarasaponin III	$C_{48}H_{76}O_{19}$	882.4977	881.4904	[2]
Tarasaponin VI	$C_{41}H_{64}O_{13}$	764.4347	763.4274	[2]
Tragopogonoside B	$C_{42}H_{66}O_{15}$	810.4402	809.4329	[38]
Yuzhizioside IV	$C_{52}H_{84}O_{21}$	1044.5505	1043.5432	[17]
Zingibroside R1 dimethyl ester	$C_{44}H_{70}O_{14}$	822.4766	821.4693	[36]
α-Hederin	$C_{41}H_{66}O_{12}$	750.4554	749.4481	[25]
Congmuyenoside I	$C_{42}H_{68}O_{14}$	764.4609	763.4536	[23]
Congmuyenoside II	$C_{48}H_{78}O_{19}$	958.5137	957.5064	[23]
Congmuyenoside III	$C_{60}H_{98}O_{29}$	1282.6193	1281.6120	[23]
Congmuyenoside IV	$C_{42}H_{68}O_{15}$	812.4558	811.4485	[23]

Table S2. Triterpene saponins annotated in the extracts of *Aralia elata var. mandshurica* (*Rupr. & Maxim.*) J. Wen roots by reversed ultra phase high performance liquid chromatography-mass spectrometry (RP-UHPLC-QqTOF-MS) in untargeted SWATH experiments (with the inclusion list containing the *m/z* of all predicted [M-H]$^-$ ions using published data)

No	t_R (min)	*m/z* [M-H]- observed	*m/z* [M-H]- calculated	Elemental composition [M-H]-	MS2 fragmentation patterns - product ions, *m/z* (rel. intensity)	Δm (ppm)	Assignment
**3**	3.7	749.4481	749.4482	$C_{41}H_{65}O_{12}^-$	455.3532 (60%),485.1508 (75%), 617.4060 (35%), 749.4481 (40%)	0.13	α-Heredin
**4**	3.4	765.4443	765.4431	$C_{41}H_{65}O_{13}^-$	471.3479 (30%), 959.3343 (20%), 633.4011 (15%), 757.3878 (100%), 765.4443 (17%)	- 1.57	Collinsonidin
**5**	3.5	778.3300	778.4509	$C_{42}H_{66}O_{13}^{-\bullet}$	425.1316 (10%), 455.3541(20%), 485.1516 (65%), 778.3300 (60%)	155	Acutoside A
**6**	4.9	781.4171	781.4380	$C_{41}H_{65}O_{14}^-$	387.2855 (90%), 401.2492 (20%), 619.3331 (10%), 665.3183 (15%), 779.4215 (10%), 777.4070 (9%), 780.4209 (8%), 781.4171 (5%)	27	Congmuyenoside A
**7**	4.6	809.4333	809.4329	$C_{42}H_{65}O_{15}^-$	556.3175 (40%), 751.4649 (30%), 767.4594 (35%), 779.4564 (15%), 781.4732 (100%), 793.4362 (90%), 809.4333 (60%)	-0.49	Elatoside
**8**	3.3	811.4519	811.4485	$C_{42}H_{67}O_{15}^-$	345.2268 (100%), 471.5677 (15%), 485.1509 (30%), 721.3301 (20%), 777.3617 (28%), 809.4022 (45%), 811.4519 (48%)	-4.2	Congmuyenoside IV
**9**	3.8	911.5015	911.5010	$C_{47}H_{75}O_{17}^-$	455.3515 (20%), 617.4057 (15%), 749.4479 (20%), 77.4590 (18%), 911.5015 (100%)	- 0.5	Guaiacin B
**10**	4.5	925.4814	925.4802	$C_{47}H_{73}O_{18}^-$	455.3541 (3%), 569.3852 (30%), 613.3746 (10%), 775.4288 (4%), 794.4439 (2%), 925.4814 (100%)	-1.3	Araloside A
**11**	3.5	927.4955	927.4959	$C_{47}H_{75}O_{18}^-$	471.3419 (20%), 633.4008 (20%), 747.4391 (18%), 765.4456 (20%), 795.4537 (40%), 927.4955 (100%)	0.5	Araliasaponin I
**12**	5.1	939.4618	939.4959	$C_{48}H_{75}O_{18}^-$	569.3852 (10%), 631.3857 (26%), 793.4388 (40%), 847.4473 (20%), 865.4611 (25%), 939.4618 (100%)	36	Araloside A methyl ester
**13**	4.6	941.4753	941.4752	$C_{47}H_{73}O_{19}^-$	455.3509 (5%), 471.3465 (5%), 631.3852 (7%), 747.4331 (35%), 793.4388 (42%), 809.4331 (3%), 927.4957 (36%), 941.4753 (100%)	-0.1	Armatoside B
**14**	3.8	941.5046	941.5115	$C_{48}H_{77}O_{18}^-$	456.3556 (3%), 585.3772 (5%), 617.4064 (7%), 779.4526 (20%), 925.4833 (16%), 941.5046 (100%)	7.3	

**15**	3.5	943.4905	943.4908	$C_{47}H_{75}O_{19}^{-}$	765.4419 (18%), 779.4282 (17%), 927.4974 (60%), 943.4905 (100%)	0.3	Araliasaponin II
**16**	3.9	953.5104	953.5115	$C_{49}H_{77}O_{18}^{-}$	455.3523 (15%), 617.4082 (12%), 749.4462 (18%), 795.4536 (9%), 822.4728 (9%), 893.4927 (10%), 911.5016 (30%), 925.4814 (26%), 953.5104 (100%)	1.2	Taibaienoside II
**17**	4.9	955.4890	955.4908	$C_{48}H_{74}O_{19}^{-}$	454.3310 (3%), 594.3418 (5%), 761.4049 (6%), 793.4257 (10%), 823.3948 (5%), 913.4877 (9%), 925.4790 (25%), 955.4890(100%)	1.9	Clendulaglucoside C
**18**	4.4	957.4942	957.5067	$C_{48}H_{77}O_{19}^{-}$	791.4209 (10%), 925.4787 (13%), 955.4912 (100%), 957.4942 (15%)	13	Congmuyanoside B
**19**	4.5	1043.5432	1043.5432	$C_{52}H_{83}O_{21}^{-}$	569.3822 (15%), 613.3765 (8%), 731.4388 (9%), 775.4326 (12%), 925.4788 (100%), 1043.5432 (9%)	0	Yuzhizioside IV
**20**	4.4	1057.5363	1057.5225	$C_{52}H_{81}O_{22}^{-}$	455.3532 (2%), 551.3732 (3%), 585.3641 (3%), 701.4259 (7%), 745.4197 (3%), 895.4713 (15%), 911.5030 (5%), 955.4884 (3%), 1057.5363 (100%)	13	Araloside B
**21**	4.2	1087.5337	1087.5335	$C_{53}H_{83}O_{23}^{-}$	455.3501 (2%), 551.3726 (2%), 702.42875 (2%), 775.4270 (2%), 793.4383 (1.5%), 925.4798 (8%), 956.4949 (5%), 1087.5337 (100%)	-0.6	Araloside C
**22**	4.5	1089.5476	1089.5487	$C_{53}H_{85}O_{23}^{-}$	455.3521 (2%), 587.3969 (5%), 719.4372 (20%), 749.4493 (13%), 881.4925 (100%), 925.4795 (12%), 1043.5444 (35%), 1089.5476 (30%)	1	Araliasaponin III
**23**	5.1	1117.5419	1117.5436	$C_{54}H_{85}O_{24}^{-}$	959.3599 (15%), 749.4479 (10%), 793.4357 (35%), 866.4649 (15%), 919.4631 (7%), 937.4834 (50%), 955.4900 (51%), 1013.5322 (47%), 1057.5603 (36%), 1099.5340 (55%), 1117.5419 (100%)	1.5	Calendulaglycoside A
**24**	4.0	1119.5585	1119.5593	$C_{54}H_{87}O_{24}$	609.3126 (10%), 775.4263 (5%), 955.4899 (7%), 1087.5365 (3%), 1119.5585 (100%)	0.7	Araliasaponin IV
**25**	3.4	1249.5862	1249.5859	$C_{59}H_{93}O_{28}$	763.4331 (10%), 795.4525 (11%), 925.4817 (100%), 1249.5862 (95%)	-0.2	Araliaarmoside

Table S3. List of compounds confirmed by MS^2 analysis in the roots of *Aralia elata var. mandshurica* (*Rupr. & Maxim*.) J. Wen

Compound Name	t_R	*m/z* $[M-H]^-$ observed	*m/z* $[M-H]^-$ of aglycone
Guaiacin B	3.8	911.4993	455.3507
Kalopanax-Saponin F	3.9	1087.5317	455.3509
Oleanolic acid-3-*O*-(triglucopyranosyl-1-3-arabinopyranosyl)-28-1-glucopyranosyl	3.8	1235.6107	455.3522
Calendulaglycoside A	3.9	1117.5490	455.3499
Araliaarmoside	4.0	1249.5869	455.3487
Calendulaglycoside C	4.3	955.4859	455.3511
Araloside B	4.4	1057.549	455.3517
Oleanolic acid-3-*O*-(diglucopyranosyl-1-3-arabinopyranosyl)-28-1-glucopyranosyl ester	4.3	1073.5569	455.3518
Oleanolic acid-3-*O*-(methyldioxy-trihexopyranosyl-1-3-pentopyranosyl)-28-1-hexopyranosyl ester	4.3	1119.5606	455.3521
Oleanolic acid-3-*O*-(hexosyl)-28-1-hexouronide ester	4.6	793.4312	455.3471
Araliasaponin III	4.5	1089.5493	455.3501
Araloside A	5.3	925.4805	455.3508
Oleanolic acid 3-*O*-hexuronide-(1-3-pentafuranoside)	5.7	763.4260	455.3502

Table S4. Triterpene saponins annotated in the extracts of *Aralia elata var. mandshurica* (*Rupr. & Maxim.*) J. Wen roots by reversed phase ultra-high performance liquid chromatography-mass spectrometry and tandem mass spectrometry (RP-UHPLC-QqTOF-MS and MS/MS) in SWATH and data-dependent acquisition (DDA) experiments

№	t_R (min)	*m/z* $[M-H]^-$ observed	*m/z* $[M-H]^-$ calculated	Elemental composition $[M-H]^-$	MS2 fragmentation patterns - product ions, *m/z* (rel. intensity)	Δm (ppm)	Assignment	Ref	Suppl. spectra
**1**	7.8	911.4993	911.501	$C_{47}H_{75}O_{17}^-$	455.3507 (20), 617.4041 (15), 749.4472 (15), 911.4993 (100)	1.9	Guaiacin B isomer 1	[30]	S1-1
**2**	7.9	1087.5317	1087.5331	$C_{53}H_{83}O_{23}^-$	455.3509 (10), 701.4265 (5), 925.4814 (15), 1087.5317 (100)	1.3	Kalopanax-Saponin F isomer 1	[31]	S1-2
**3**	8.0	1235.6107	1235.6066	$C_{59}H_{95}O_{27}^-$	455.3522 (50), 617.4059 (15), 749.4492 (100), 911.5035 (10), 1235.6107 (10)	-3.3	Oleanolic acid-3-O-(triglucopyranosyl-1-3-arabinopyranosyl)-28-1-glucopyranosyl		S1-3
**4**	8.2	1117.539	1117.5436	$C_{54}H_{85}O_{24}^-$	455.3499 (3), 731.4347 (5), 955.4898 (10), 1117.5390 (100)	4.1	Calendulaglycoside A	[15]	S1-4
**5**	8.4	1249.5869	1249.5859	$C_{59}H_{93}O_{28}^-$	455.3487 (5), 701.4254 (15), 925.4743 (7), 1057.5219 (80), 1087.5337 (50), 1153.5538 (100), 1249.5869 (70)	-0.8	Araliaarmoside	[8]	S1-5
**6**	8.4	1087.5336	1087.5331	$C_{53}H_{83}O_{23}^-$	455.3509 (10), 701.4265 (5), 925.4814 (15), 1087.55336 (100)	-0.5	Kalopanax-Saponin F isomer 2	[31]	S1-2
**7**	8.6	955.4859	955.4908	$C_{48}H_{75}O_{19}^-$	455.3511 (5), 569.379 (5), 793.4317 (15), 955.4859 (100)	5.1	Calendulaglycoside C isomer 1	[15]	S1-6
**8**	8.7	1057.5249	1057.5225	$C_{52}H_{81}O_{22}^-$	455.3517 (5), 701.4286 (7), 763.4309 (5), 895.4698 (7), 1057.5249 (100)	-2.3	Araloside B isomer 2	[11]	S1-7
**9**	8.9	1073.5569	1073.5538	$C_{53}H_{85}O_{22}^-$	455.3518 (50), 617.4052 (20), 749.4485 (30), 911.5022 (100), 1073.5569 (15)	-2.9	Oleanolic acid-3-O-(diglucopyranosyl-1-3-arabinopyranosyl)-28-1-glucopyranosyl ester		S1-8
**10**	8.9	1119.5606	1119.5616	$C_{54}H_{87}O_{24}^-$	455.3521 (5), 617.4057 (5), 749.4490 (7), 911.5019 (100), 1073.5551 (15), 1119.5606 (13)	0.9	Oleanolic acid-3-O-(ethyl-trihexopyranosyl-1-3-pentopyranosyl)-		S1-9

							28-1-hexopyranosyl ester		
**11**	9.0	793.4312	793.438	$C_{42}H_{65}O_{14}^{-}$	455.3471 (10), 631.3785 (15), 793.4312 (100)	8.6	Oleanolic acid-3-O-(hexosyl)-28-1-hexouronide ester isomer 1		S1-10
**12**	9.3	1089.5493	1089.5487	$C_{53}H_{85}O_{23}^{-}$	455.3501 (5), 719.4360 (10), 881.4901 (100), 1043.5437 (12), 1089.5493 (12)	-0.6	Araliasaponin III	[6]	S1-11
**13**	10.1	955.4899	955.4908	$C_{48}H_{75}O_{19}^{-}$	455.3511 (5), 569.379 (5), 793.4317 (15), 955.4899 (100)	0.9	Calendulaglycoside C isomer 2	[15]	S1-6
**14**	10.3	925.4805	925.4802	$C_{47}H_{73}O_{18}^{-}$	455.3508 (2), 569.3831 (5), 731.4366 (20), 925.4803 (100)	0.3	Araloside A isomer 1	[11]	S1-12
**15**	10.4	925.4807	925.4802	$C_{47}H_{73}O_{18}^{-}$	455.3508 (2), 569.3831 (5), 731.4366 (20), 925.4803 (100)	0.5	Araloside A isomer 2	[11]	S1-12
**16**	10.8	895.4696	-	-	455.3508 (5), 551.3731 (5), 895.4696 (100)	-	Oleanolic acid unknown derivatives		S1-13
**17**	10.8	793.436	793.438	$C_{42}H_{65}O_{14}^{-}$	455.3471 (10), 631.3785 (15), 793.4312 (100)	2.5	Oleanolic acid-3-O-(hexosyl)-28-1-hexouronide ester isomer 2		S1-10
**18**	10.9	763.426	763.4274	$C_{41}H_{63}O_{13}^{-}$	455.3502 (3), 631.3822 (5), 763.4260 (100)	1.8	Oleanolic acid 3-O-hexuronide-(1-3-pentafuranoside)		S1-14
**19**	11.0	911.5016	911.501	$C_{47}H_{75}O_{17}^{-}$	455.3507 (20), 617.4041 (15), 749.4472 (15), 911.5016 (100)	-0.7	Guaiacin B isomer 2	[30]	S1-1
**20**	11.0	911.4949	911.501	$C_{47}H_{75}O_{17}^{-}$	455.3507 (20), 617.4041 (15), 749.4472 (15), 911.4949 (100)	6.7	Guaiacin B isomer 3	[30]	S1-1

The extracts were prepared with conventional (water or ethanol) or natural deep eutectic solvents (NADES) and analyzed by a Waters ACQUITY UPLC I-Class UPLC System (Waters GmbH, Eschborn, Germany) coupled online to a hybrid quadrupole-time of flight mass spectrometer (QqTOF-MS) AB Sciex TripleTOF 6600 (AB Sciex, Darmstadt, Germany)

Table S5. The conditions of reverse phase-ultra high performance liquid chromatographic (RP-UHPLC) separation and the settings for electrospray ionization-quadrupole-time of flight mass spectrometry (ESI-QqTOF-MS) applied for the profiling (untargeted analysis) of *Aralia elata var. mandshurica* (Rupr. & Maxim.) J. Wen root semi-polar secondary metabolites with QqTOF hybride mass spectrometer SHIMADZU LCMS-9030 System (SHIMADZU Corporation, Kyoto, Japan).

Chromatography

**SIL-30AC Autosampler**	
Injection mode	Partial Loop
Injection volume	5 µL
Wash solvent	50% MeOH
Wash solvent volume	300 µL
Cooler temperature	4.0 C
Rinse type	Internal & external
Rinse mode	Before and after aspiration
Needle overfill flush	Rinse port + rinse pump
Rinse time	2 sec
**Column conditions**	
Separation column	Phenomenex Kinetex C18 Column (100 x 2.1 mm, particle size 1.7 µm)
Column oven temperature	40.0 C
**LC separation parameters**	
Eluent A	aqueous 0.1% (*v/v*) FA
Eluent B	0.1% (*v/v*) FA in acetonitrile
Flow rate	0.5 mL/min

Elution program	gradient to 100% eluent B – 10 min<br>100% eluent B isocratic – 2 min<br>gradient to 5% eluent B – 0.1 min<br>5% eluent B isocratic – 0.9 min (re-equilibration)

Mass spectrometry

**General**	
Mass analyzer type	quadrupole-time of flight (QqTOF-MS)
Ionsource	ESI
Experiment type	Sequential Windowed Acquisition of All Theoretical Fragment Ion Mass Spectra (DIA)
Operatinon mode	positive, negative
Cycle time (s)	0.996
Duration	10.0 min
**Ion source settings**	
Nebulizer gas (L/min)	3
Drying gas (L/min)	10
Ion spray voltage (kV)	4.0/-3.0 (negative mode)
Ion source temperature (°C)	180
	**MS settings**
Experiment type	TOF-MS
m/z range	240 - 1350
Accumulation time (ms)	100
ID function	ON
**MS/MS Setting**	

Experiment type	SWATH
Collision gas	Ar
MS/MS experiment type	DIA
SWATH window number	28 u (650-1350 *m/z*)
SWATH window width (*m/z*)	25.0
CE	18-52 (35 ± 17 V)
Accumulation time (ms)	21
ID function	OFF
Collision potential (V)	35/-35 (negative mode)
Collision energy spread (V)	17 (negative mode)

SWATH, sequential window acquisition of all theoretical fragment ion spectra

Table S6. The conditions of reversed phase-ultrahigh performance liquid chromatographic (RP-UHPLC) separation and the settings for electrospray ionization-quadrupole-time of flight mass spectrometry (ESI-QqTOF-MS) applied for the SWATH and DDA MS/MS analysis of *Aralia elata var. mandshurica* (Rupr. & Maxim.) J. Wen root semi-polar secondary metabolites with Waters ACQUITY UPLC I-Class UPLC System (Waters GmbH, Eschborn, Germany) coupled online to a hybrid quadrupole-time of flight mass spectrometer (QqTOF-MS) AB Sciex TripleTOF 6600 (AB Sciex, Darmstadt, Germany).

Chromatography

**ACQUITY Sample Manager (SM)**	
Injection mode	Partial Loop
Injection volume	5 µL
Weak wash solvent	0.3 mmol/L aq. ammonium formate, pH 3.5 (adjusted using formic acid)
Weak wash volume	800 µL
Strong wash solvent	acetonitrile
Strong wash volume	400 µL
Target sample temperature	4.0 C
Needle overfill flush	automatic
**Column conditions**	
Separation column	EC 150/2 NUCLEOSHELL RP 18 (150 x 2 mm, particle size 2.7 µm)
Target column temperature	40.0 C
**ACQUITY Binary Solvent Manager (BSM)**	
Eluent A	0.3 mmol/L aq. ammonium formate, pH 3.5 (adjusted using formic acid)
Eluent B	acetonitrile

Seal wash duration	5 min
Flow rate	0.4 mL/min
Elution program	5% eluent B isocratic - 2 min<br>gradient to 95% eluent B – 17 min<br>95% eluent B isocratic – 2 min<br>gradient to 5% eluent B – 0.1 min<br>5% eluent B isocratic – 3 min (re-equilibration)

Mass spectrometry

**General**	
Mass analyzer type	quadrupole-time of flight (QqTOF-MS)
Ionsource	DuoSpray™ ion source
Experiment type	Sequential Windowed Acquisition of All Theoretical Fragment Ion Mass Spectra (SWATH)
Operatinon mode	negative
Cycle time (s)	1.1
Pause between ranges (ms)	1.049
Auto adjust with mass	on
Settling time (s)	0
Time bins to sum	4
Duration	23 min
**Ion source settings**	
Nebulizer gas (psig)	60
Drying gas (psig)	70
Curtain gas (psig)	55

Ion spray voltage (kV)	-4.5
Ion source temperature (°C)	450
**MS settings**	
Experiment type	TOF-MS
m/z range	65 - 1250
Accumulation time (ms)	50/75 (SWATH/DDA)
Declustering potential (V)	-35
Collision potential (V)	-10
**MS/MS Setting**	
Fragmentation mode	collision-activated dissociation (CAD)
MS/MS experiment type	DDA
For ions greater than	100 Da
Charge state	1-2
With intensity greater than	3000
Maximum number of candidate ions to monitor per cycle	5
Exclude former target ions for	4 sec
Fragment intensity multiplier	2
Maximum accumulation	2 sec
Analyte type	small molecules
Accumulation time (ms)	175
Declustering potential (V)	-35
Collision potential (V)	-45
Collision energy spread (V)	35
Ion release delay (V)	-30

Ion release width (V)	-15
**MS/MS Setting**	
Fragmentation mode	collision-activated dissociation (CAD)
MS/MS experiment type	SWATH
SWATH window number	15
SWATH window width (*m/z*)	80
SWATH window overlap (*m/z*)	1
Rolling collision energy	off
Analyte type	small molecules
Accumulation time (ms)	60
Declustering potential (V)	-35
Collision potential (V)	-45
Collision energy spread (V)	35
Ion release delay (V)	-30
Ion release width (V)	-15

Figures

Chromatographic and mass spectral data of triterpene saponins annotated in the extracts of *Aralia elata var. mandshurica* (*Rupr. & Maxim.*) J. Wen roots by reversed phase ultra-high performance liquid chromatography-mass spectrometry and tandem mass spectrometry (RP-UHPLC-QqTOF-MS and MS/MS) in SWATH and data-dependent acquisition (DDA) experiments

A

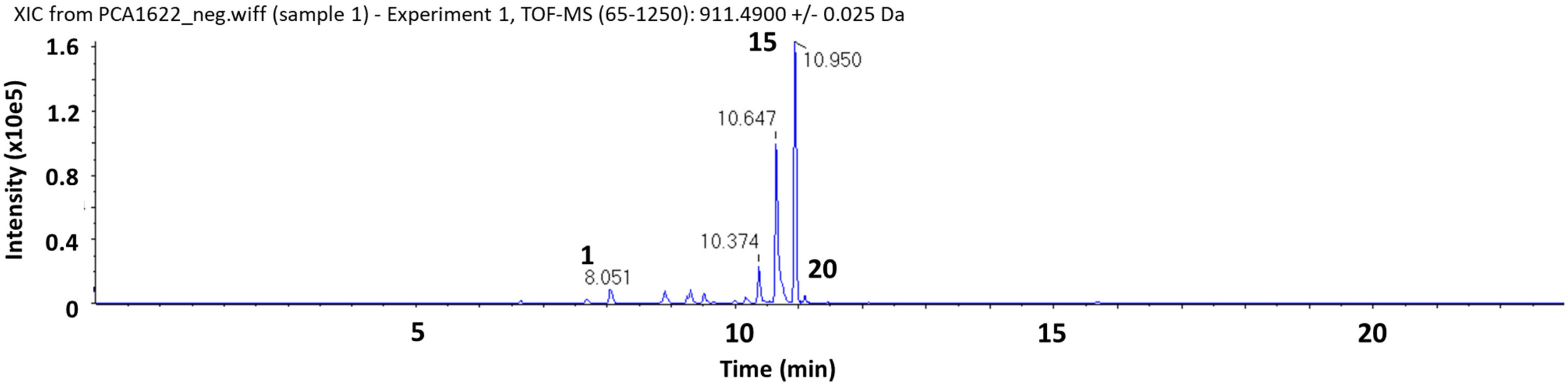

B

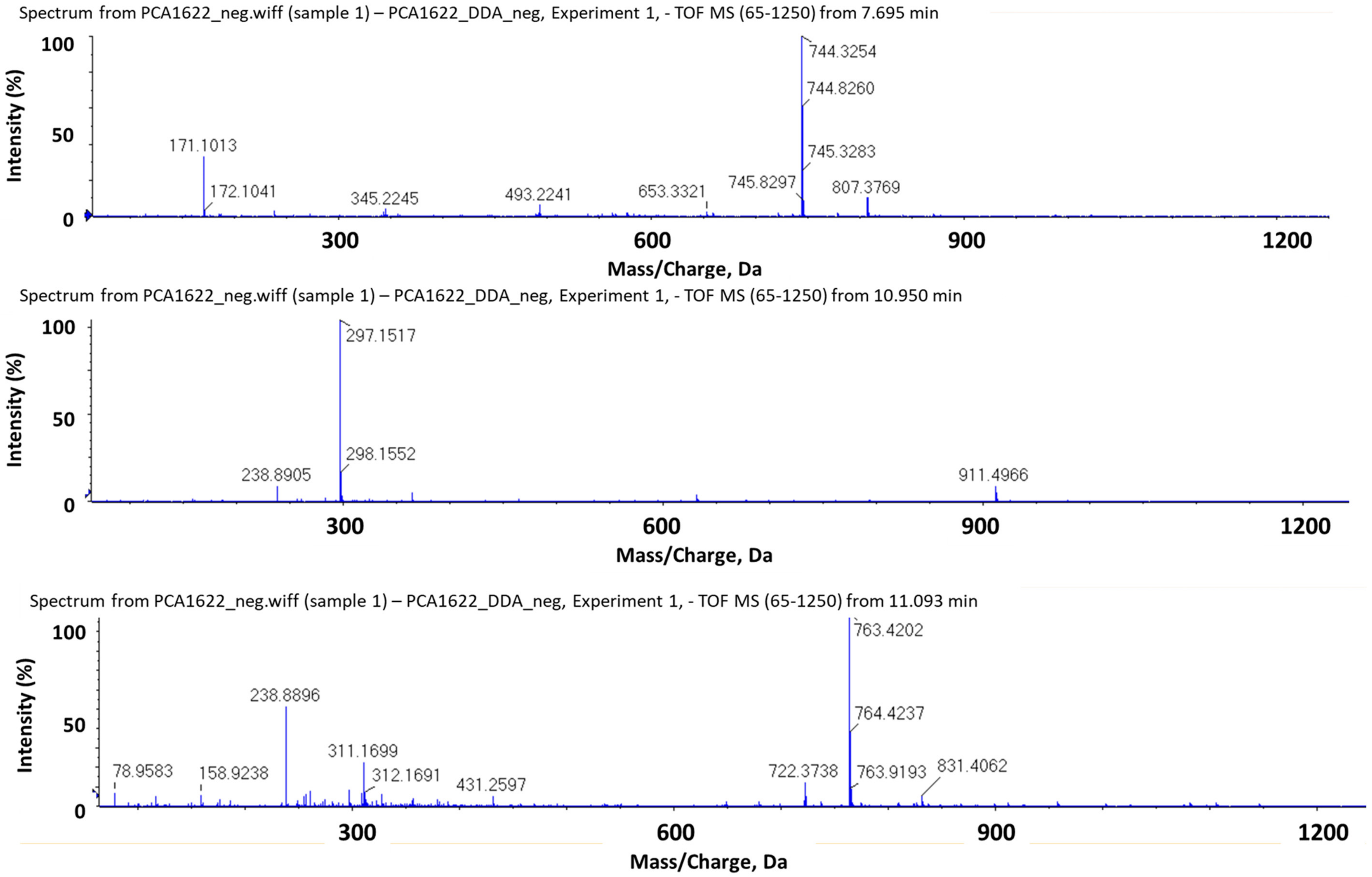
Spectrum from PCA1622_neg.wiff (sample 1) – PCA1622_DDA_neg, Experiment 1, - TOF MS (65-1250) from 7.695 min
Intensity (%)
100
50
0
744.3254
744.8260
745.3283
745.8297
807.3769
171.1013
172.1041
345.2245
493.2241
653.3321
300
600
900
1200
Mass/Charge, Da
Spectrum from PCA1622_neg.wiff (sample 1) – PCA1622_DDA_neg, Experiment 1, - TOF MS (65-1250) from 10.950 min
Intensity (%)
100
50
0
297.1517
298.1552
238.8905
911.4966
300
600
900
1200
Mass/Charge, Da
Spectrum from PCA1622_neg.wiff (sample 1) – PCA1622_DDA_neg, Experiment 1, - TOF MS (65-1250) from 11.093 min
Intensity (%)
100
50
0
763.4202
764.4237
763.9193
831.4062
722.3738
238.8896
311.1699
312.1691
431.2597
78.9583
158.9238
300
600
900
1200
Mass/Charge, Da

C

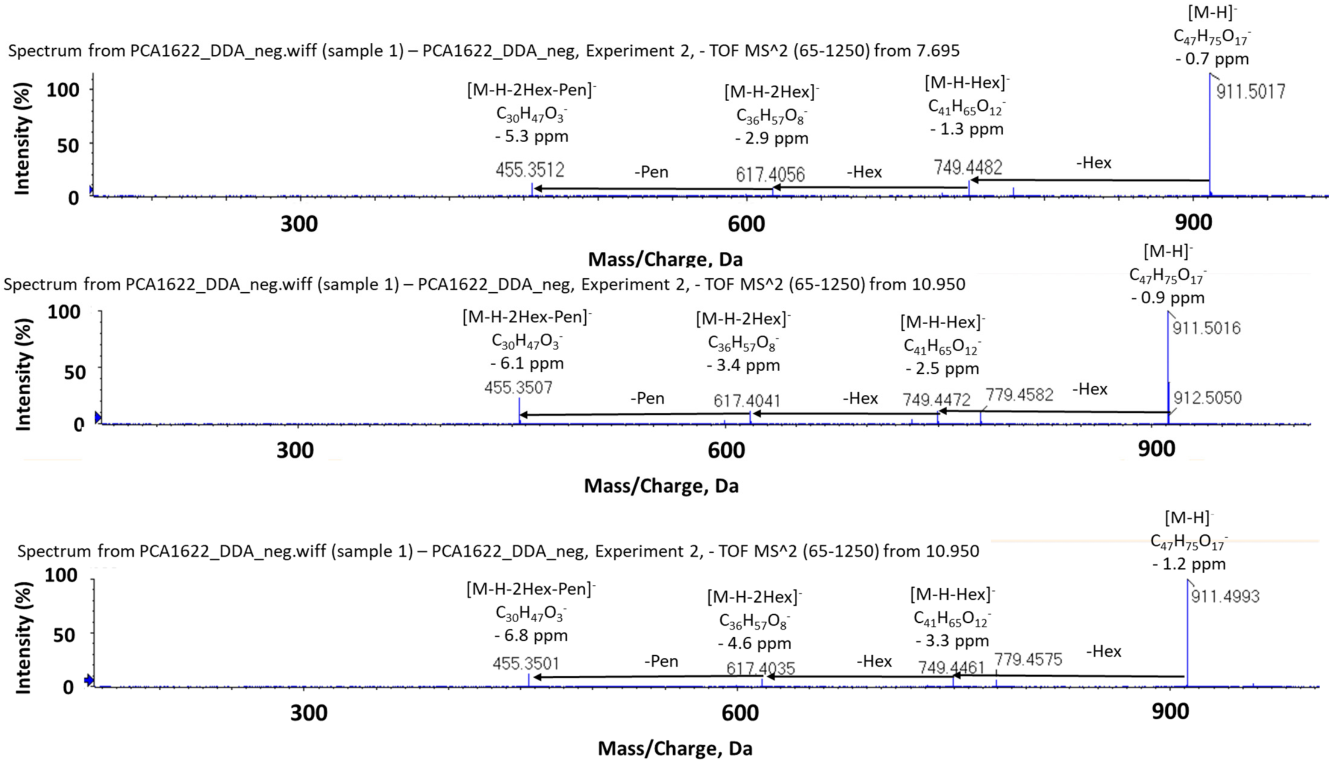

Figure S1. Extracted ion chromatogram (XIC) of *m/z* 911.5000 ± 0.02 (A), the MS spectra (B) and MS/MS fragmentation patterns (C) of the compounds 1, 15, 20 annotated in the total ethanolic extract of *Aralia elata var. mandshurica* (*Rupr. & Maxim*.) J. Wen roots as guaiacin B isomers at t_R 7.77, 10.98 and 11.01, respectively. The analysis relied on RP-UHPLC-QqTOF-MS accomplished with a Waters ACQUITY I-Class UPLC System (Waters GmbH, Eschborn, Germany) coupled on-line to a Triple-TOF6600 hybrid mass spectrometer (Sciex, Darmstadt, Germany) in the negative ion mode. Metabolites were annotated by reversed phase ultra-high performance liquid chromatography-mass spectrometry and tandem mass spectrometry (RP-UHPLC-QqTOF-MS and MS/MS) in targeted data-dependent acquisition (DDA) experiments (with the inclusion list containing the m/z of all predicted [M-H]- ions annotated at the MS1 level).

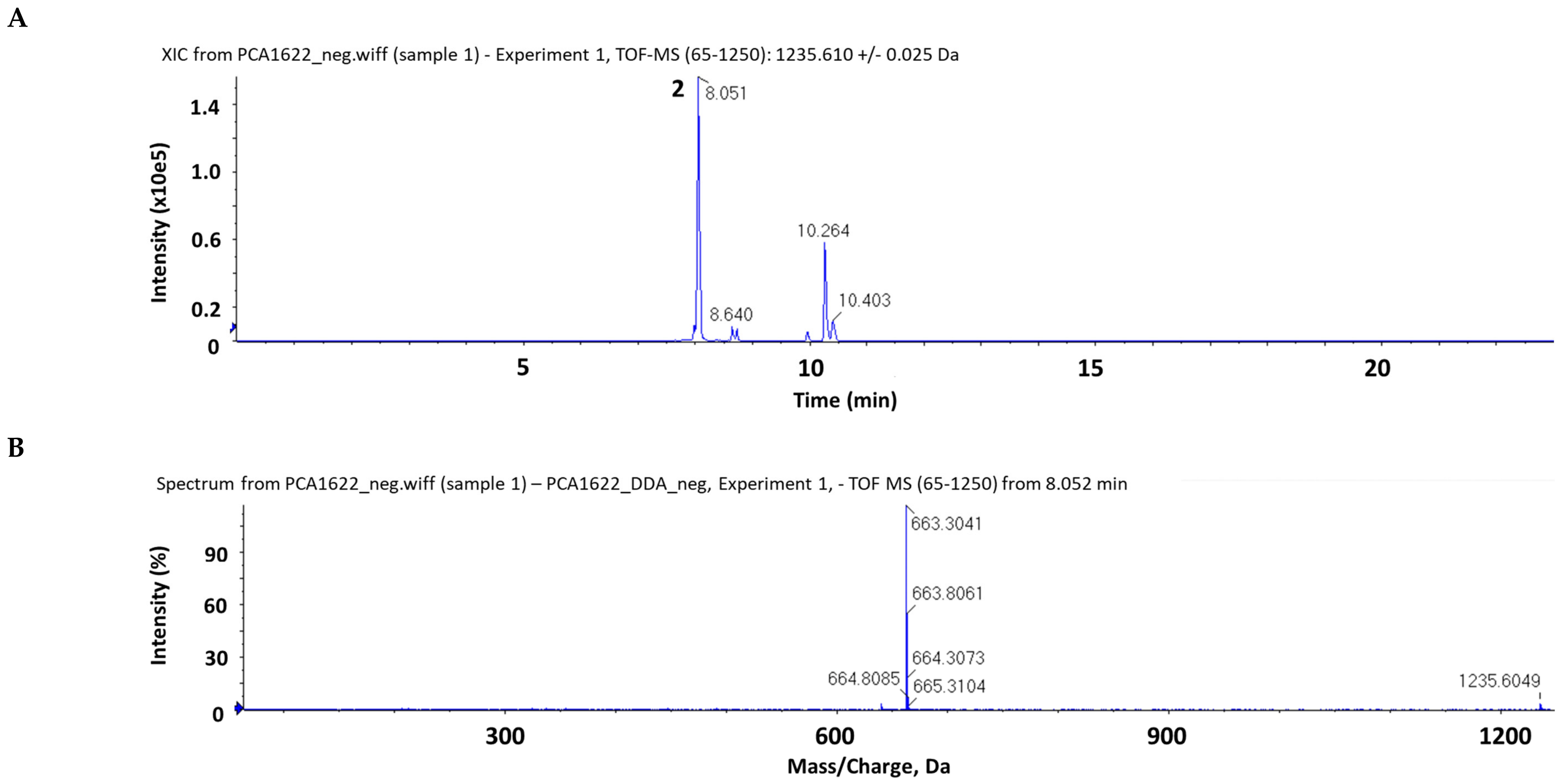
A
XIC from PCA1622_neg.wiff (sample 1) - Experiment 1, TOF-MS (65-1250): 1235.610 +/- 0.025 Da
2
8.051
8.640
10.264
10.403
Intensity (x10e5)
1.4
1.0
0.6
0.2
0
5
10
15
20
Time (min)
B
Spectrum from PCA1622_neg.wiff (sample 1) – PCA1622_DDA_neg, Experiment 1, - TOF MS (65-1250) from 8.052 min
663.3041
663.8061
664.3073
664.8085
665.3104
1235.6049
Intensity (%)
90
60
30
0
300
600
900
1200
Mass/Charge, Da

C

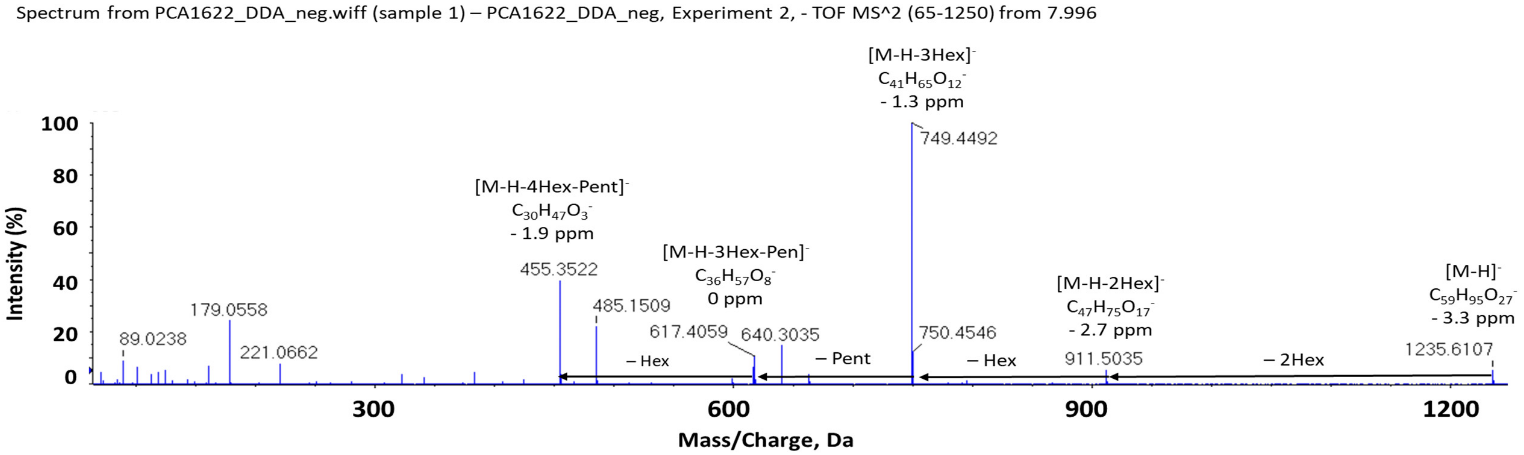

Figure S2. Extracted ion chromatogram *m/z* 1235.6100 ± 0.02 (A), the MS spectra (B) and MS/MS fragmentation patterns (C) of the compounds 2 annotated in the total ethanolic extract of *Aralia elata var. mandshurica* (*Rupr. & Maxim.*) J. Wen roots as oleanolic acid-3-*O*-(triglucopyranosyl-1-3-arabinopyranosyl)-28-1-glucopyranosyl. The analysis relied on RP-UHPLC-QqTOF-MS accomplished with a Waters ACQUITY I-Class UPLC System (Waters GmbH, Eschborn, Germany) coupled on-line to a Triple-TOF6600 hybrid mass spectrometer (Sciex, Darmstadt, Germany) in the negative ion mode. Metabolites were annotated by reversed phase ultra-high performance liquid chromatography-mass spectrometry and tandem mass spectrometry (RP-UHPLC-QqTOF-MS and MS/MS) in targeted data-dependent acquisition (DDA) experiments (with the inclusion list containing the *m/z* of all predicted $[M-H]^-$ ions annotated at the MS1 level).

A

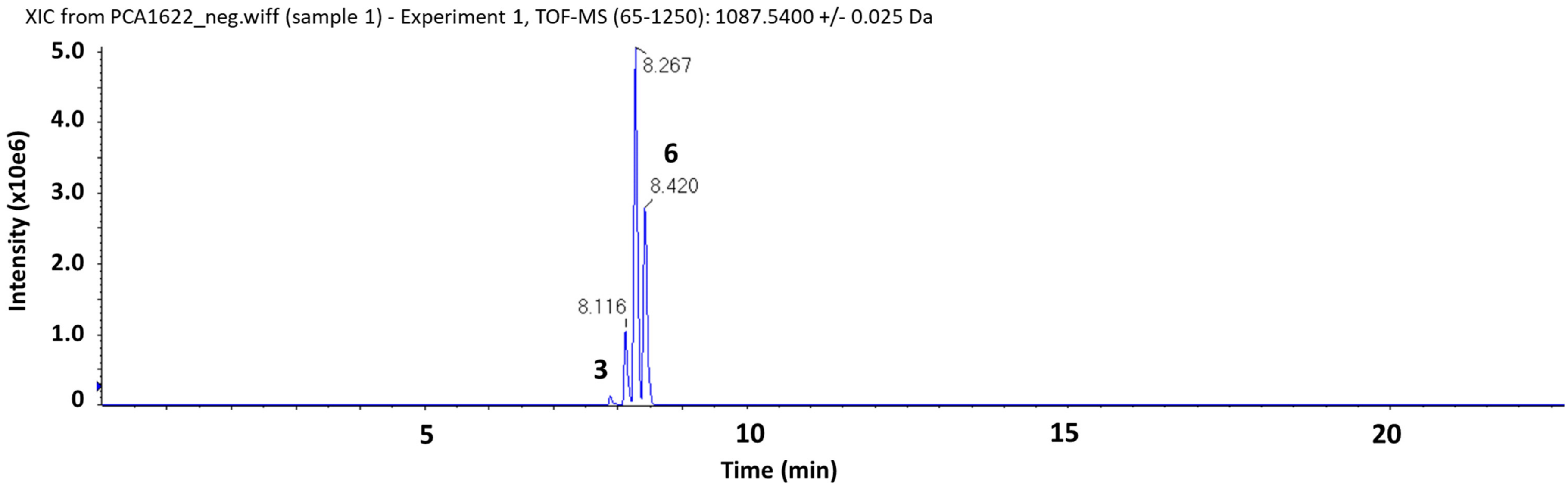
XIC from PCA1622_neg.wiff (sample 1) - Experiment 1, TOF-MS (65-1250): 1087.5400 +/- 0.025 Da
Intensity (x10e6)
5.0
4.0
3.0
2.0
1.0
0
8.267
6
8.420
8.116
3
5
10
15
20
Time (min)

B

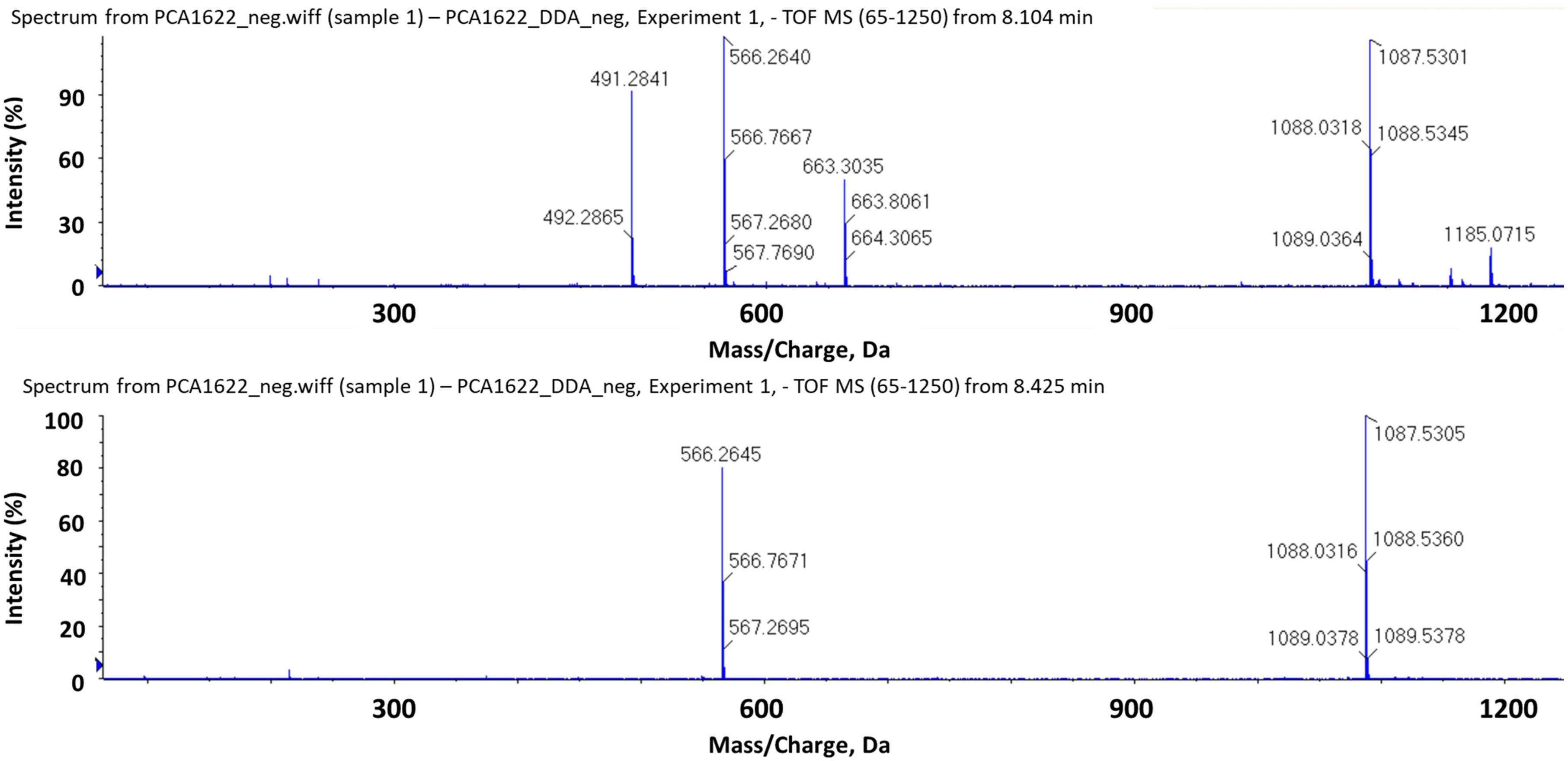

Spectrum from PCA1622_neg.wiff (sample 1) – PCA1622_DDA_neg, Experiment 1, - TOF MS (65-1250) from 8.104 min
Intensity (%)
90
60
30
0
491.2841
492.2865
566.2640
566.7667
567.2680
567.7690
663.3035
663.8061
664.3065
1087.5301
1088.0318
1088.5345
1089.0364
1185.0715
300
600
900
1200
Mass/Charge, Da
Spectrum from PCA1622_neg.wiff (sample 1) – PCA1622_DDA_neg, Experiment 1, - TOF MS (65-1250) from 8.425 min
Intensity (%)
100
80
60
40
20
0
566.2645
566.7671
567.2695
1087.5305
1088.0316
1088.5360
1089.0378
1089.5378
300
600
900
1200
Mass/Charge, Da

C

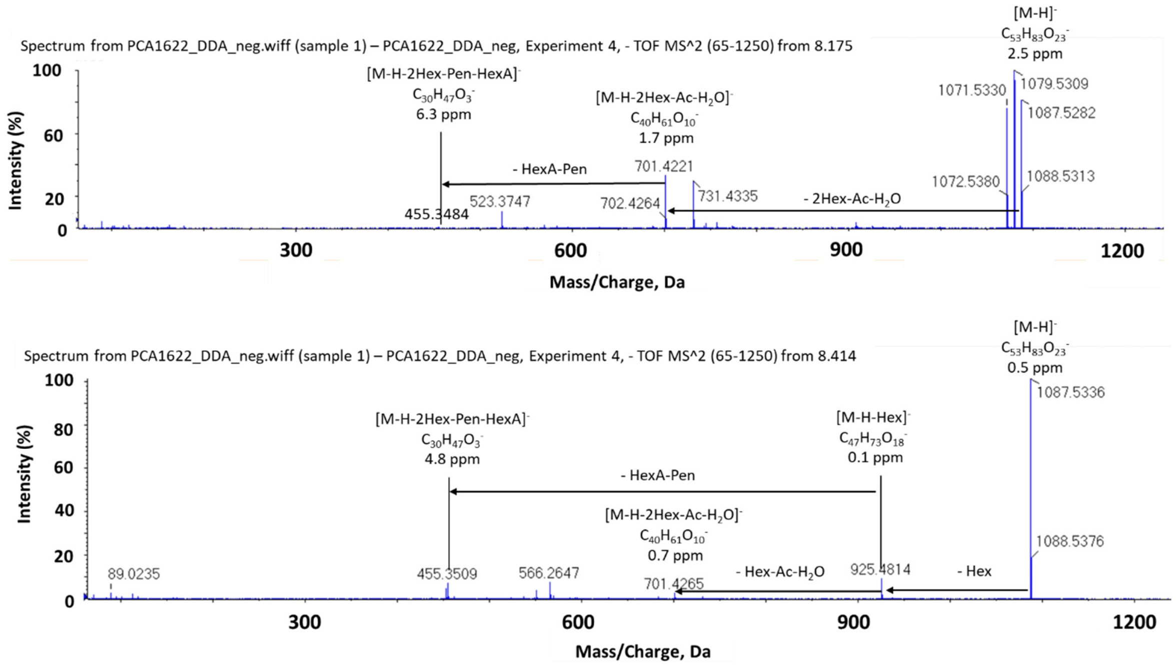

Figure S3. Extracted ion chromatogram *m/z* 1087.5400 ± 0.02 (A), the MS spectra (B) and MS/MS fragmentation patterns (C) of the compounds 3, 6 annotated in the total ethanolic extract of *Aralia elata var. mandshurica* (*Rupr. & Maxim.*) J. Wen roots as kalopanax-saponin F isomers at t_R 7.89 and 8.41, respectively. The analysis relied on RP-UHPLC-QqTOF-MS accomplished with a Waters ACQUITY I-Class UPLC System (Waters GmbH, Eschborn, Germany) coupled on-line to a Triple-TOF6600 hybrid mass spectrometer (Sciex, Darmstadt, Germany) in the negative ion mode. Metabolites were annotated by reversed phase ultra-high performance liquid chromatography-mass spectrometry and tandem mass spectrometry (RP-UHPLC-QqTOF-MS and MS/MS) in targeted data-dependent acquisition (DDA) experiments (with the inclusion list containing the *m/z* of all predicted [M-H]$^-$ ions annotated at the MS1 level).

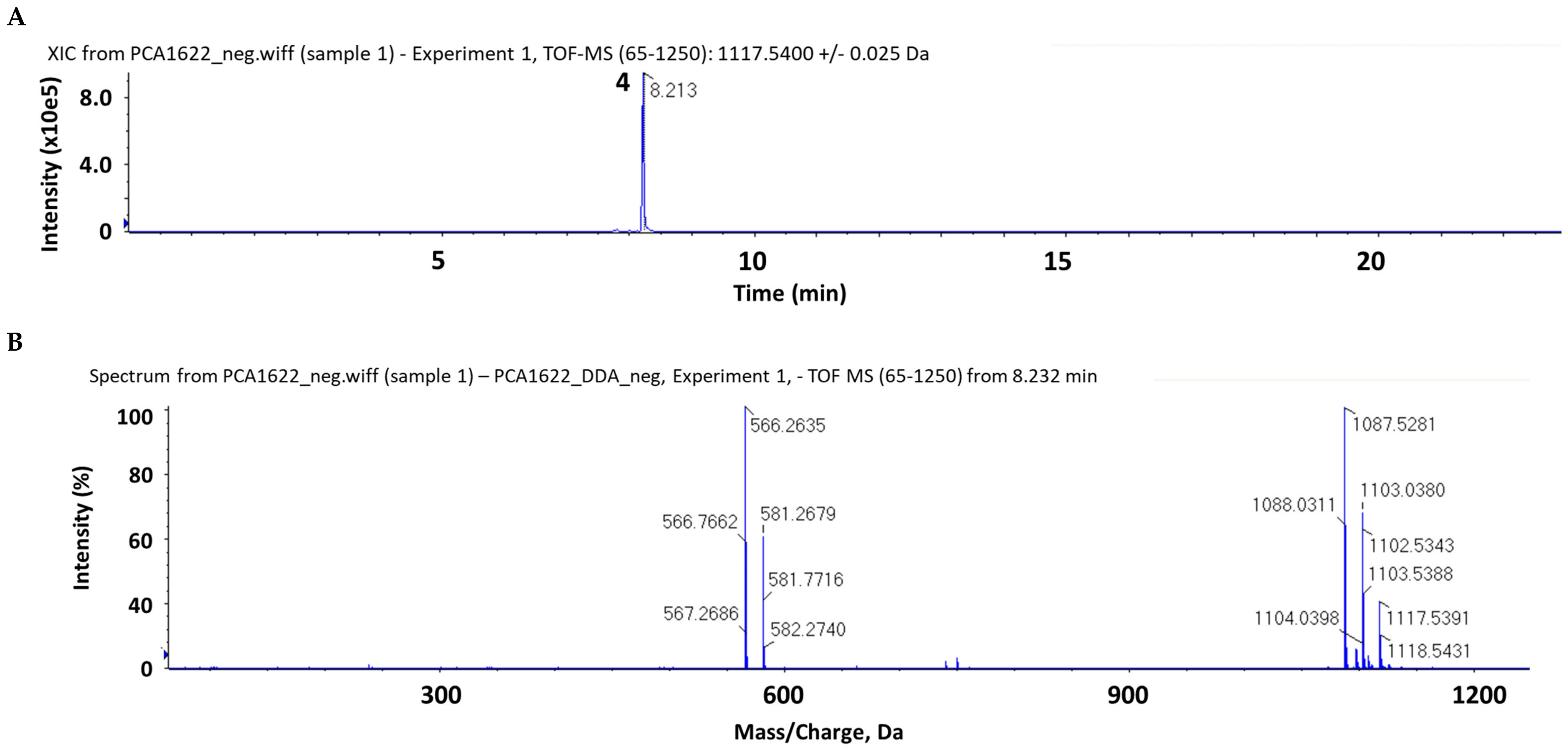
A
XIC from PCA1622_neg.wiff (sample 1) - Experiment 1, TOF-MS (65-1250): 1117.5400 +/- 0.025 Da
Intensity (x10e5)
8.0
4.0
0
4
8.213
5
10
15
20
Time (min)
B
Spectrum from PCA1622_neg.wiff (sample 1) – PCA1622_DDA_neg, Experiment 1, - TOF MS (65-1250) from 8.232 min
Intensity (%)
100
80
60
40
0
566.2635
566.7662
567.2686
581.2679
581.7716
582.2740
1087.5281
1088.0311
1103.0380
1102.5343
1103.5388
1104.0398
1117.5391
1118.5431
300
600
900
1200
Mass/Charge, Da

C

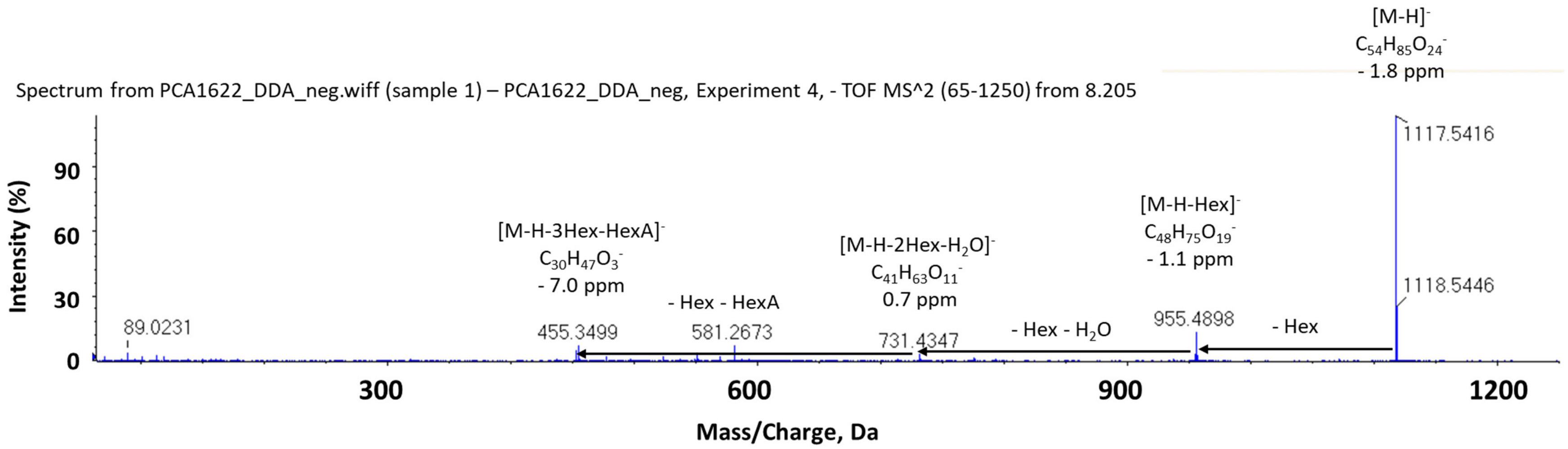

Figure S4. Extracted ion chromatogram m/z 1117.5400 ± 0.02 (A), the MS spectra (B) and MS/MS fragmentation patterns (C) of the compounds 4 annotated in the total ethanolic extract of Aralia elata var. mandshurica (Rupr. & Maxim.) J. Wen roots as calendulaglycoside A. The analysis relied on RP-UHPLC-QqTOF-MS accomplished with a Waters ACQUITY I-Class UPLC System (Waters GmbH, Eschborn, Germany) coupled on-line to a Triple-TOF6600 hybrid mass spectrometer (Sciex, Darmstadt, Germany) in the negative ion mode. Metabolites were annotated by reversed phase ultra-high performance liquid chromatography-mass spectrometry and tandem mass spectrometry (RP-UHPLC-QqTOF-MS and MS/MS) in targeted data-dependent acquisition (DDA) experiments (with the inclusion list containing the m/z of all predicted [M-H]- ions annotated at the MS1 level).

A

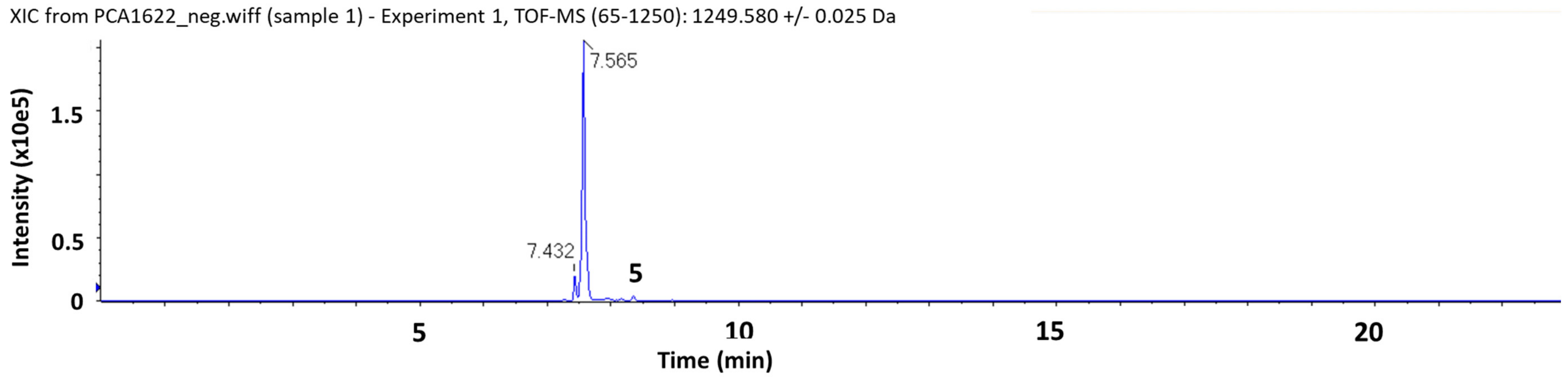
XIC from PCA1622_neg.wiff (sample 1) - Experiment 1, TOF-MS (65-1250): 1249.580 +/- 0.025 Da
Intensity (x10e5)
1.5
0.5
0
7.565
7.432
5
5
10
15
20
Time (min)

B

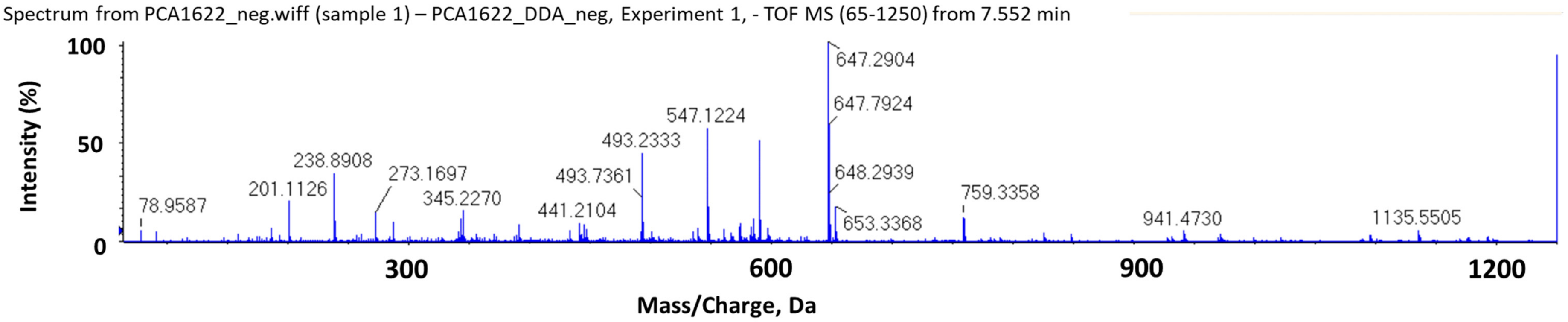
Spectrum from PCA1622_neg.wiff (sample 1) – PCA1622_DDA_neg, Experiment 1, - TOF MS (65-1250) from 7.552 min
Intensity (%)
100
50
0
647.2904
647.7924
547.1224
493.2333
238.8908
273.1697
493.7361
648.2939
201.1126
345.2270
759.3358
78.9587
441.2104
653.3368
941.4730
1135.5505
300
600
900
1200
Mass/Charge, Da

C

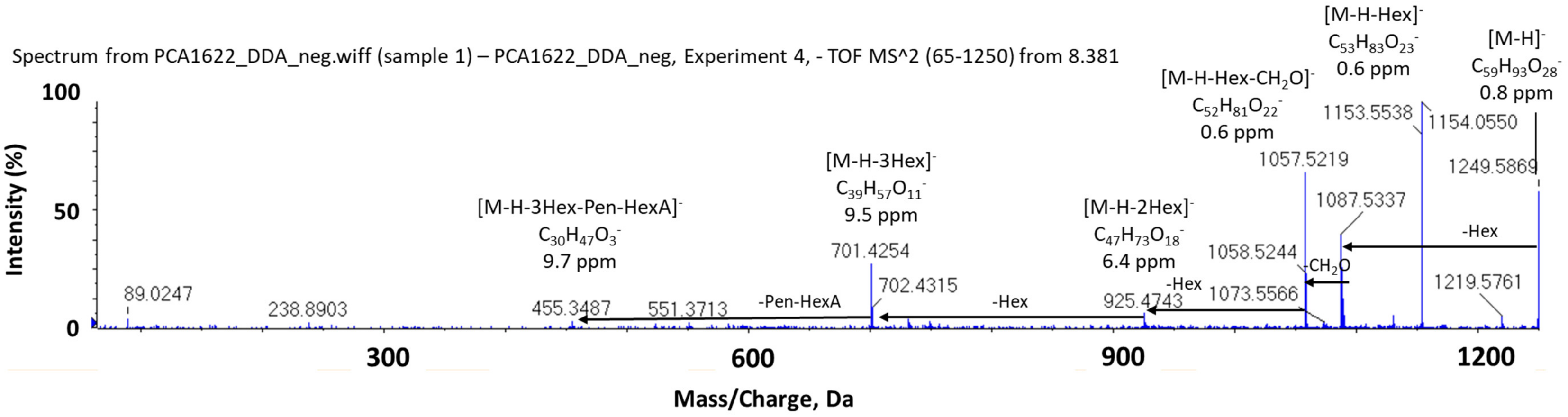

Figure S5. Extracted ion chromatogram *m/z* 1249.5900 ± 0.02 (A), the MS spectra (B) and MS/MS fragmentation patterns (C) of the compounds 5 annotated in the total ethanolic extract of *Aralia elata var. mandshurica* (*Rupr. & Maxim.*) J. Wen roots as araliaarmoside. The analysis relied on RP-UHPLC-QqTOF-MS accomplished with a Waters ACQUITY I-Class UPLC System (Waters GmbH, Eschborn, Germany) coupled on-line to a Triple-TOF6600 hybrid mass spectrometer (Sciex, Darmstadt, Germany) in the negative ion mode. Metabolites were annotated by reversed phase ultra-high performance liquid chromatography-mass spectrometry and tandem mass spectrometry (RP-UHPLC-QqTOF-MS and MS/MS) in targeted data-dependent acquisition (DDA) experiments (with the inclusion list containing the *m/z* of all predicted $[M-H]^-$ ions annotated at the MS1 level).

A

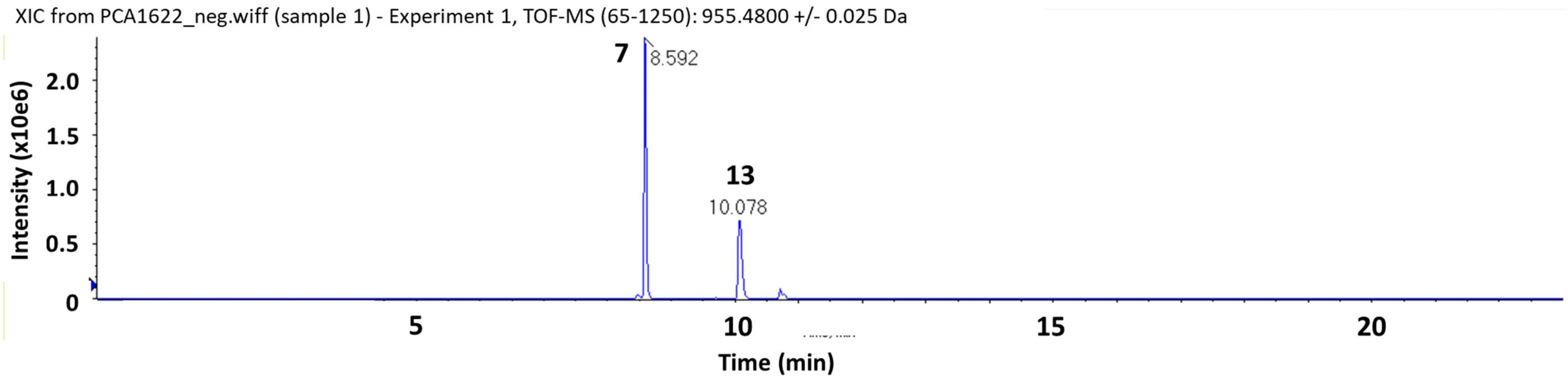

XIC from PCA1622_neg.wiff (sample 1) - Experiment 1, TOF-MS (65-1250): 955.4800 +/- 0.025 Da
7
8.592
13
10.078
Intensity (x10e6)
2.0
1.5
1.0
0.5
0
5
10
15
20
Time (min)

B

Spectrum from PCA1622_neg.wiff (sample 1) – PCA1622_DDA_neg, Experiment 1, - TOF MS (65-1250) from 8.592 min

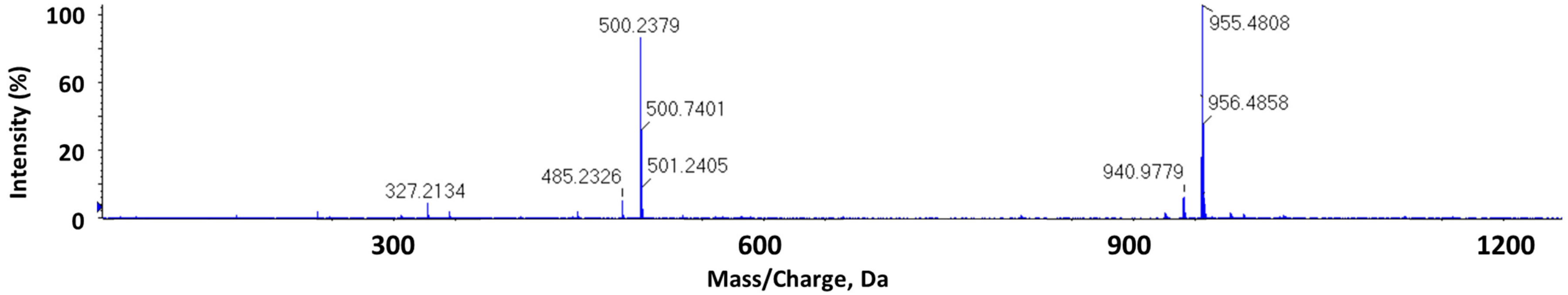

Spectrum from PCA1622_neg.wiff (sample 1) – PCA1622_DDA_neg, Experiment 1, - TOF MS (65-1250) from 10.091 min

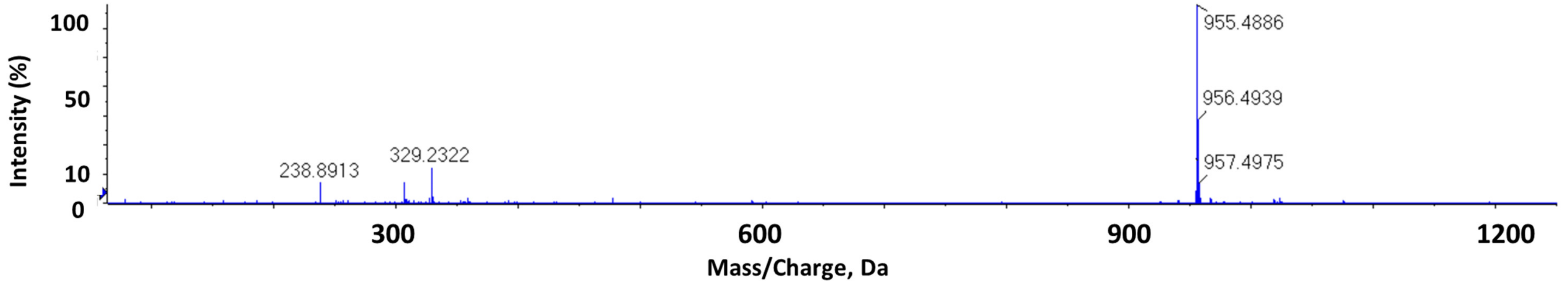

C

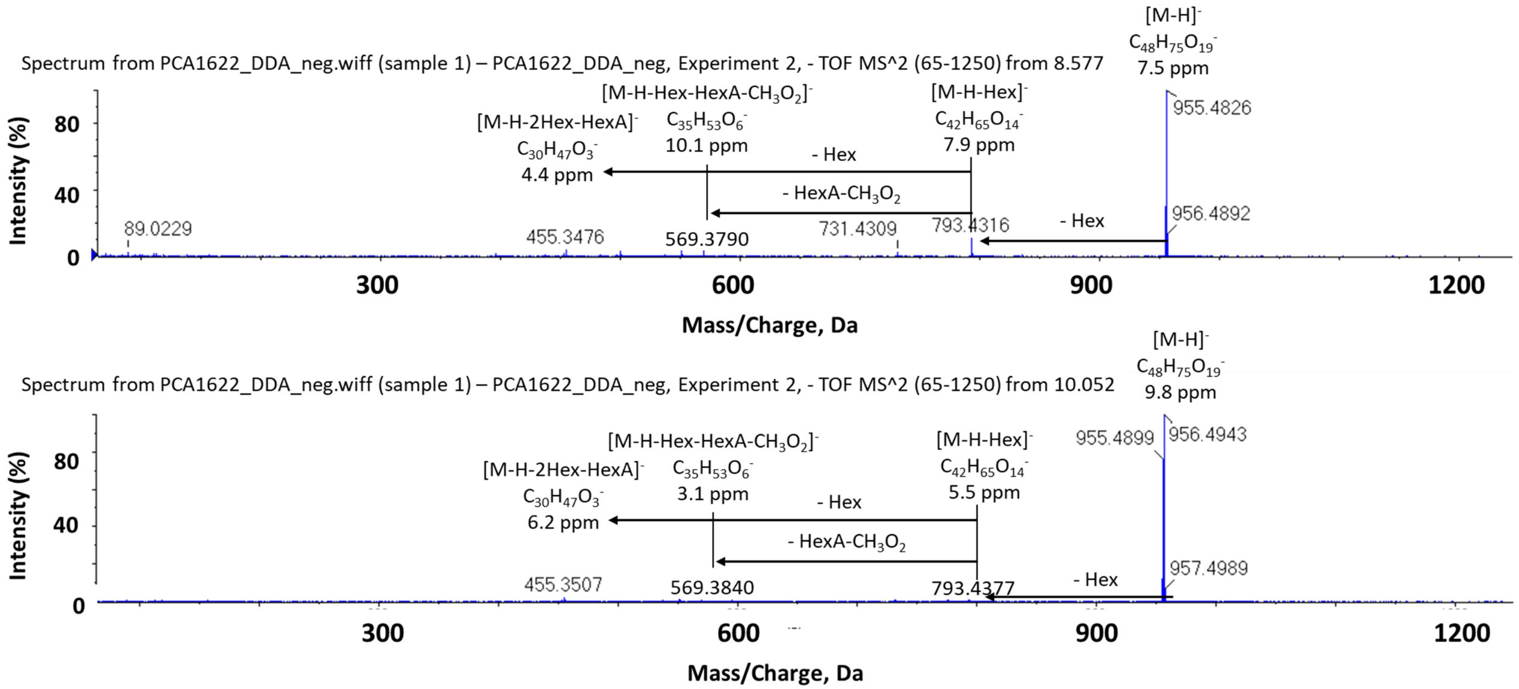

Figure S6. Extracted ion chromatogram *m/z* 955.4900 ± 0.02 (A), the MS spectra (B) and MS/MS fragmentation patterns (C) of the compounds 7, 13 annotated in the total ethanolic extract of *Aralia elata var. mandshurica* (*Rupr. & Maxim.*) J. Wen roots as calendulaglycoside C isomers at t_R 8.59 and 10.07, respectively. The analysis relied on RP-UHPLC-QqTOF-MS accomplished with a Waters ACQUITY I-Class UPLC System (Waters GmbH, Eschborn, Germany) coupled on-line to a Triple-TOF6600 hybrid mass spectrometer (Sciex, Darmstadt, Germany) in the negative ion mode. Metabolites were annotated by reversed phase ultra-high performance liquid chromatography-mass spectrometry and tandem mass spectrometry (RP-UHPLC-QqTOF-MS and MS/MS) in targeted data-dependent acquisition (DDA) experiments (with the inclusion list containing the *m/z* of all predicted $[M-H]^-$ ions annotated at the MS1 level).

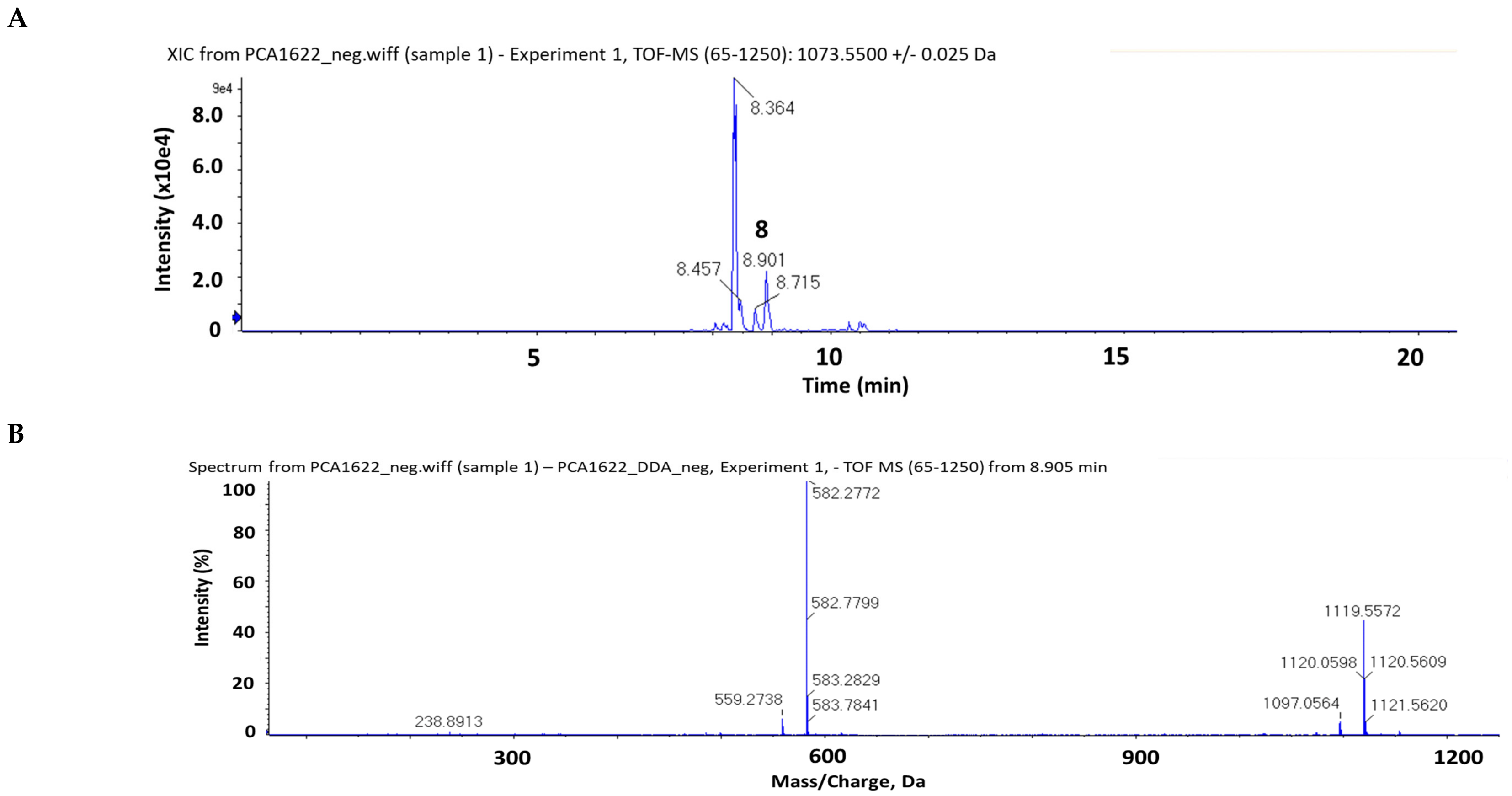
A
XIC from PCA1622_neg.wiff (sample 1) - Experiment 1, TOF-MS (65-1250): 1073.5500 +/- 0.025 Da
9e4
Intensity (x10e4)
8.0
6.0
4.0
2.0
0
8.364
8
8.457
8.901
8.715
5
10
15
20
Time (min)
B
Spectrum from PCA1622_neg.wiff (sample 1) – PCA1622_DDA_neg, Experiment 1, - TOF MS (65-1250) from 8.905 min
100
80
60
40
20
0
Intensity (%)
582.2772
582.7799
583.2829
583.7841
559.2738
238.8913
1119.5572
1120.0598
1120.5609
1097.0564
1121.5620
300
600
900
1200
Mass/Charge, Da

C

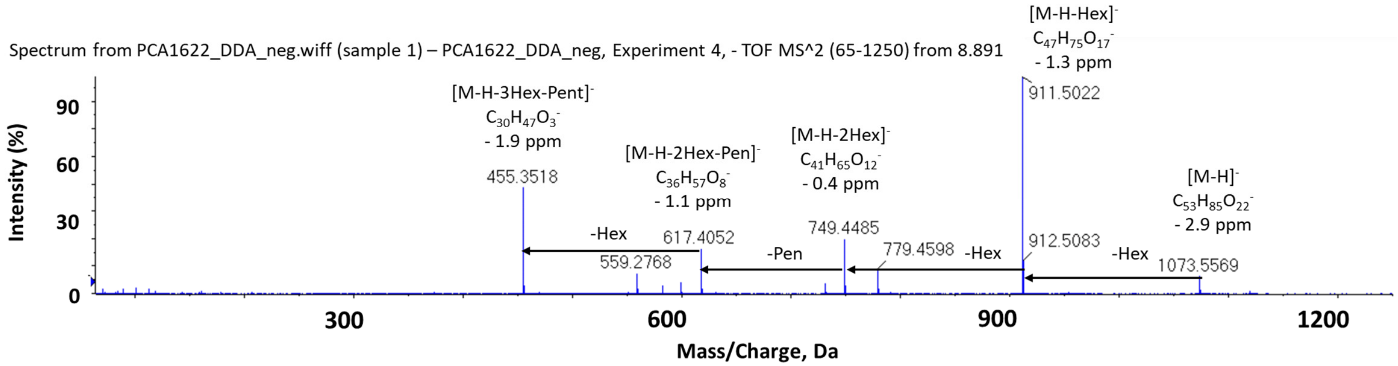

Figure S7. Extracted ion chromatogram m/z 1073.5600 ± 0.02 (A), the MS spectra (B) and MS/MS fragmentation patterns (C) of the compounds 8 annotated in the total ethanolic extract of *Aralia elata var. mandshurica* (*Rupr. & Maxim.*) J. Wen roots as oleanolic acid-3-*O*-(diglucopyranosyl-1-3-arabinopyranosyl)-28-1-glucopyranosyl ester. The analysis relied on RP-UHPLC-QqTOF-MS accomplished with a Waters ACQUITY I-Class UPLC System (Waters GmbH, Eschborn, Germany) coupled on-line to a Triple-TOF6600 hybrid mass spectrometer (Sciex, Darmstadt, Germany) in the negative ion mode. Metabolites were annotated by reversed phase ultra-high performance liquid chromatography-mass spectrometry and tandem mass spectrometry (RP-UHPLC-QqTOF-MS and MS/MS) in targeted data-dependent acquisition (DDA) experiments (with the inclusion list containing the m/z of all predicted [M-H]- ions annotated at the MS1 level).

A

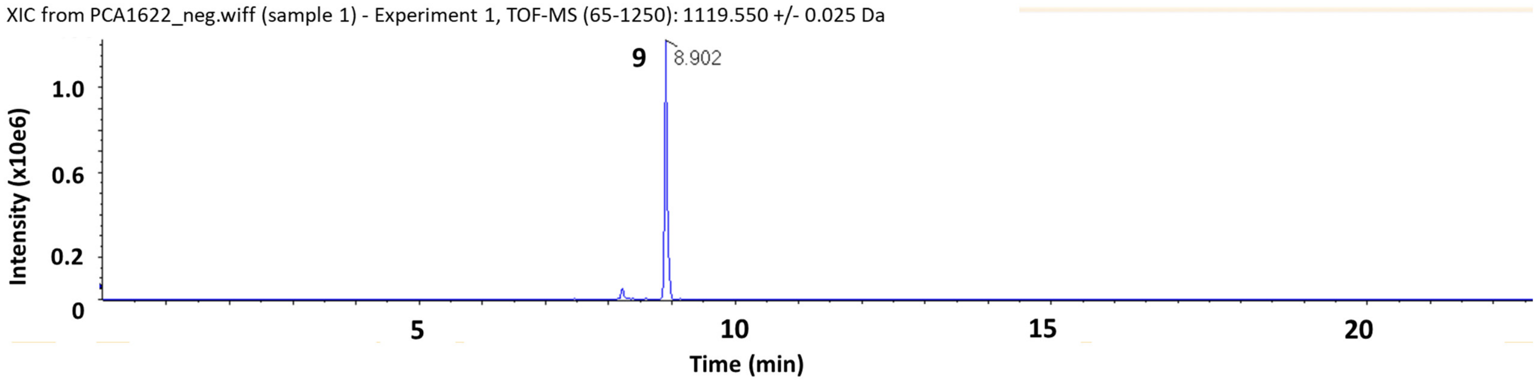
XIC from PCA1622_neg.wiff (sample 1) - Experiment 1, TOF-MS (65-1250): 1119.550 +/- 0.025 Da
9
8.902
Intensity (x10e6)
1.0
0.6
0.2
0
5
10
15
20
Time (min)

B

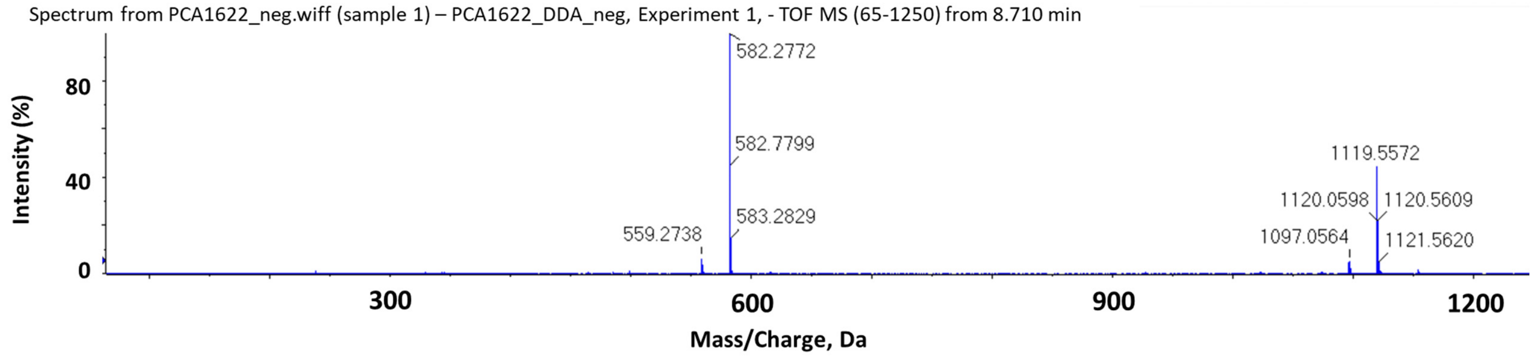
Spectrum from PCA1622_neg.wiff (sample 1) – PCA1622_DDA_neg, Experiment 1, - TOF MS (65-1250) from 8.710 min
582.2772
582.7799
583.2829
559.2738
1119.5572
1120.0598
1120.5609
1097.0564
1121.5620
Intensity (%)
80
40
0
300
600
900
1200
Mass/Charge, Da

C

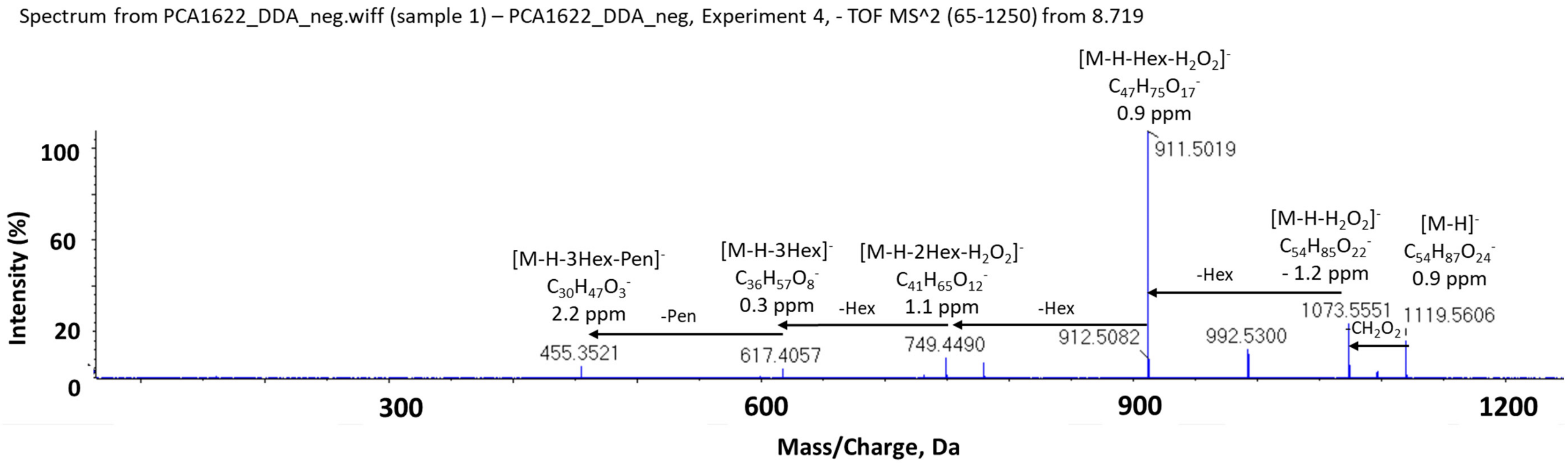

Figure S8. Extracted ion chromatogram m/z 1119.5600 ± 0.02 (A), the MS spectra (B) and MS/MS fragmentation patterns (C) of the compounds 9 annotated in the total ethanolic extract of *Aralia elata var. mandshurica* (*Rupr. & Maxim.*) J. Wen roots as oleanolic acid-3-*O*-(methyldioxy-trihexopyranosyl-1-3-pentopyranosyl)-28-1-hexopyranosyl ester. The analysis relied on RP-UHPLC-QqTOF-MS accomplished with a Waters ACQUITY I-Class UPLC System (Waters GmbH, Eschborn, Germany) coupled on-line to a Triple-TOF6600 hybrid mass spectrometer (Sciex, Darmstadt, Germany) in the negative ion mode. Metabolites were annotated by reversed phase ultra-high performance liquid chromatography-mass spectrometry and tandem mass spectrometry (RP-UHPLC-QqTOF-MS and MS/MS) in targeted data-dependent acquisition (DDA) experiments (with the inclusion list containing the *m/z* of all predicted $[M-H]^-$ ions annotated at the MS1 level).

A

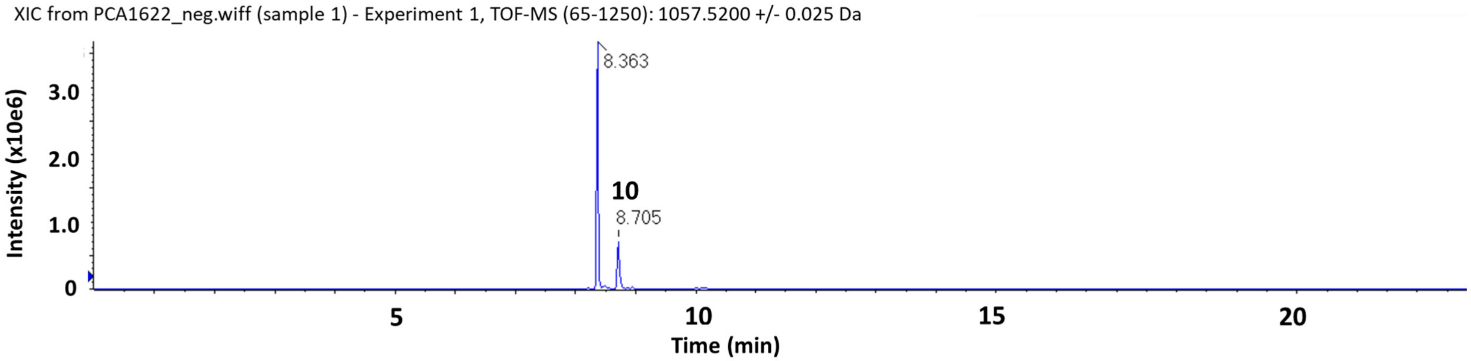
XIC from PCA1622_neg.wiff (sample 1) - Experiment 1, TOF-MS (65-1250): 1057.5200 +/- 0.025 Da
Intensity (x10e6)
3.0
2.0
1.0
0
8.363
10
8.705
5
10
15
20
Time (min)

B

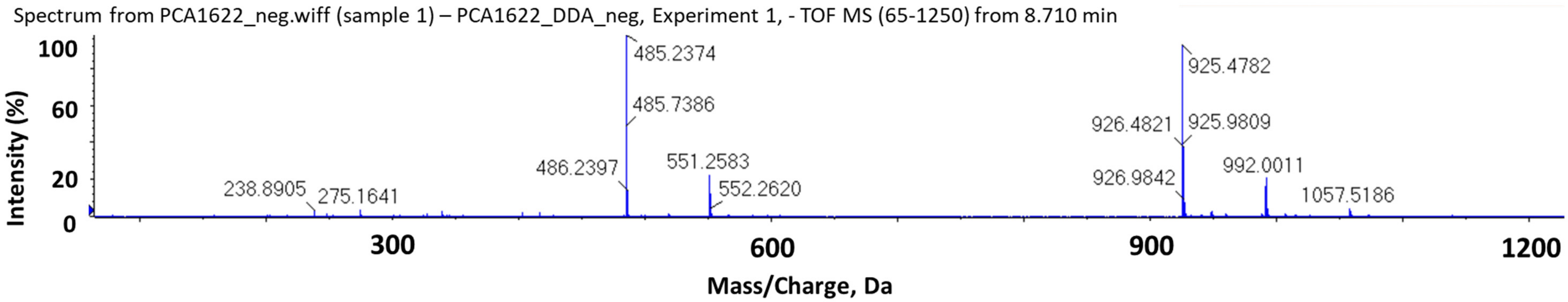
Spectrum from PCA1622_neg.wiff (sample 1) – PCA1622_DDA_neg, Experiment 1, - TOF MS (65-1250) from 8.710 min
Intensity (%)
100
60
20
0
485.2374
485.7386
486.2397
551.2583
552.2620
238.8905
275.1641
925.4782
926.4821
925.9809
926.9842
992.0011
1057.5186
300
600
900
1200
Mass/Charge, Da

C

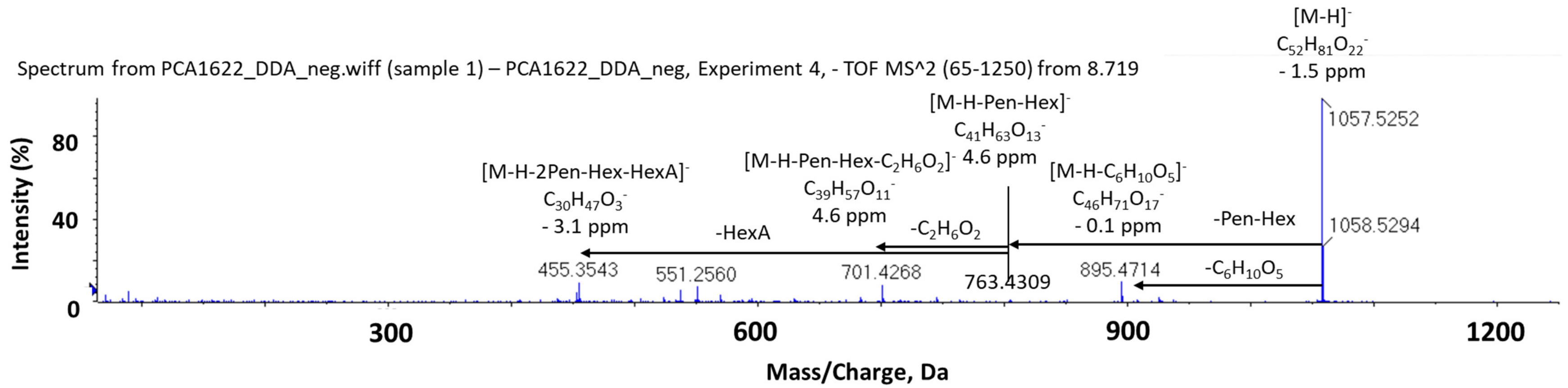

Figure S9. Extracted ion chromatogram m/z 1057.5300 ± 0.02 (A), the MS spectra (B) and MS/MS fragmentation patterns (C) of the compounds 10 annotated in the total ethanolic extract of *Aralia elata var. mandshurica* (*Rupr. & Maxim.*) J. Wen roots as araloside B. The analysis relied on RP-UHPLC-QqTOF-MS accomplished with a Waters ACQUITY I-Class UPLC System (Waters GmbH, Eschborn, Germany) coupled on-line to a Triple-TOF6600 hybrid mass spectrometer (Sciex, Darmstadt, Germany) in the negative ion mode. Metabolites were annotated by reversed phase ultra-high performance liquid chromatography-mass spectrometry and tandem mass spectrometry (RP-UHPLC-QqTOF-MS and MS/MS) in targeted data-dependent acquisition (DDA) experiments (with the inclusion list containing the *m/z* of all predicted $[M-H]^-$ ions annotated at the MS1 level).

A

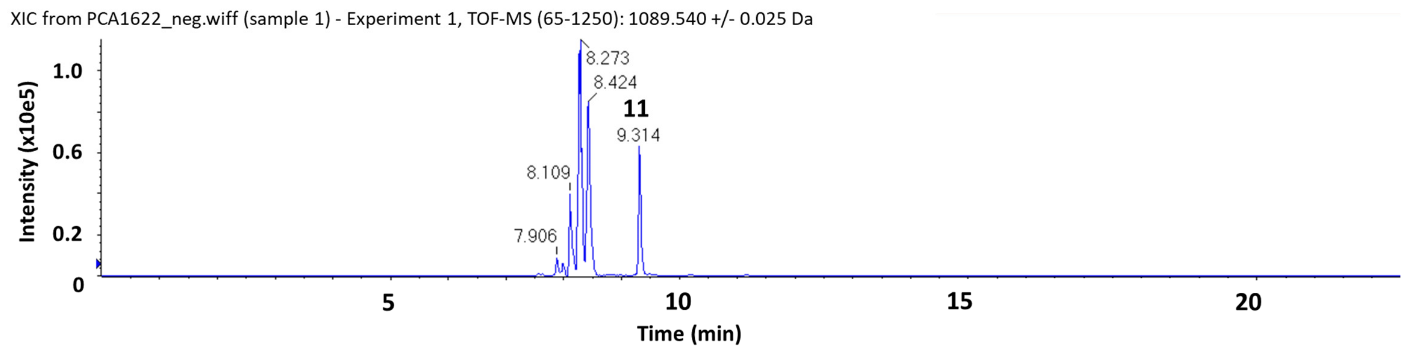
XIC from PCA1622_neg.wiff (sample 1) - Experiment 1, TOF-MS (65-1250): 1089.540 +/- 0.025 Da
Intensity (x10e5)
1.0
0.6
0.2
0
8.273
8.424
11
9.314
8.109
7.906
5
10
15
20
Time (min)

B

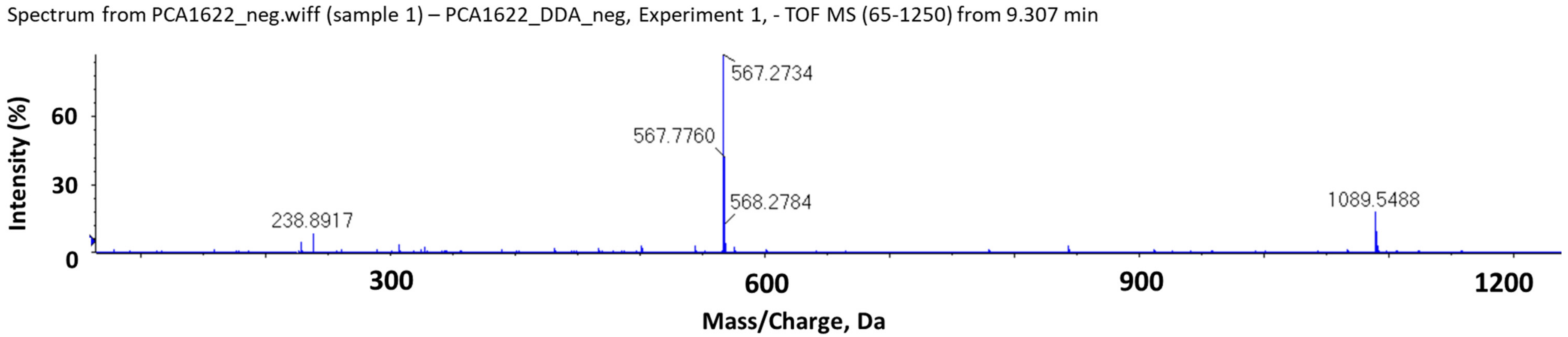
Spectrum from PCA1622_neg.wiff (sample 1) – PCA1622_DDA_neg, Experiment 1, - TOF MS (65-1250) from 9.307 min
Intensity (%)
60
30
0
567.2734
567.7760
568.2784
238.8917
1089.5488
300
600
900
1200
Mass/Charge, Da

C

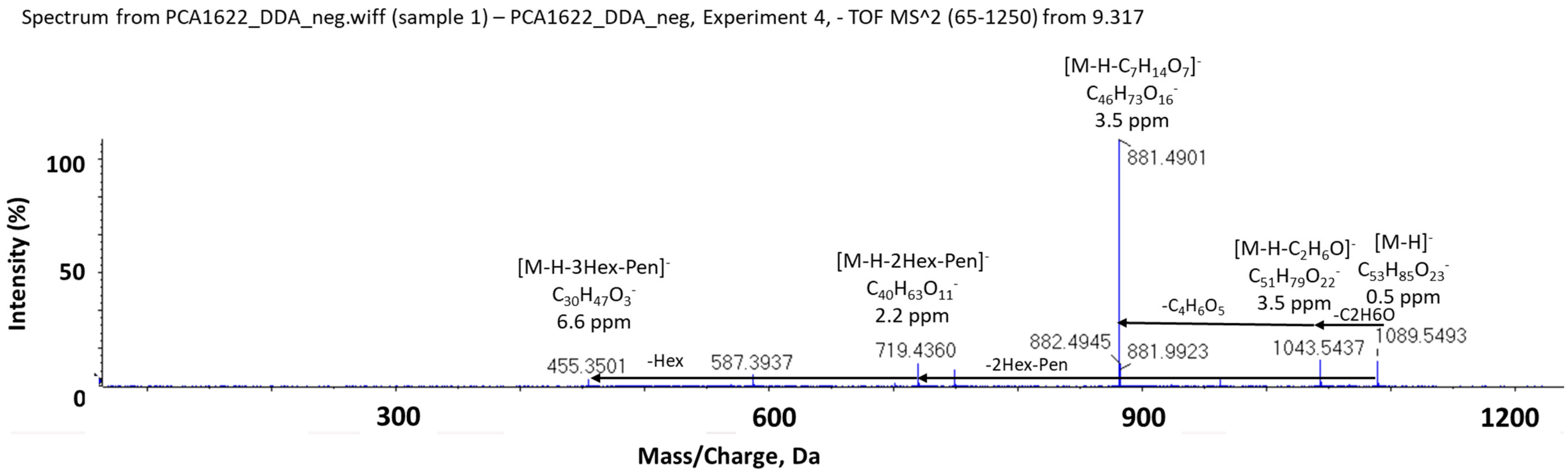

Figures S10. Extracted ion chromatogram m/z 1089.5500 ± 0.02 (A), the MS spectra (B) and MS/MS fragmentation patterns (C) of the compounds 11 annotated in the total ethanolic extract of *Aralia elata var. mandshurica* (*Rupr. & Maxim.*) J. Wen roots as araliasaponin III. The analysis relied on RP-UHPLC-QqTOF-MS accomplished with a Waters ACQUITY I-Class UPLC System (Waters GmbH, Eschborn, Germany) coupled on-line to a Triple-TOF6600 hybrid mass spectrometer (Sciex, Darmstadt, Germany) in the negative ion mode. Metabolites were annotated by reversed phase ultra-high performance liquid chromatography-mass spectrometry and tandem mass spectrometry (RP-UHPLC-QqTOF-MS and MS/MS) in targeted data-dependent acquisition (DDA) experiments (with the inclusion list containing the *m/z* of all predicted $[M\text{-}H]^-$ ions annotated at the MS1 level).

A

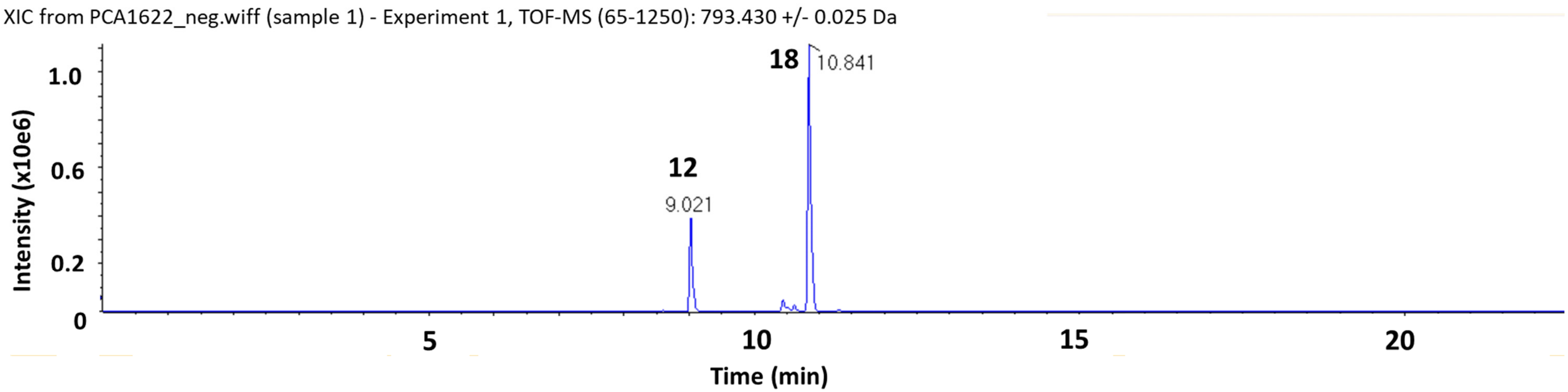
XIC from PCA1622_neg.wiff (sample 1) - Experiment 1, TOF-MS (65-1250): 793.430 +/- 0.025 Da
18
10.841
12
9.021
1.0
0.6
0.2
0
Intensity (x10e6)
5
10
15
20
Time (min)

B

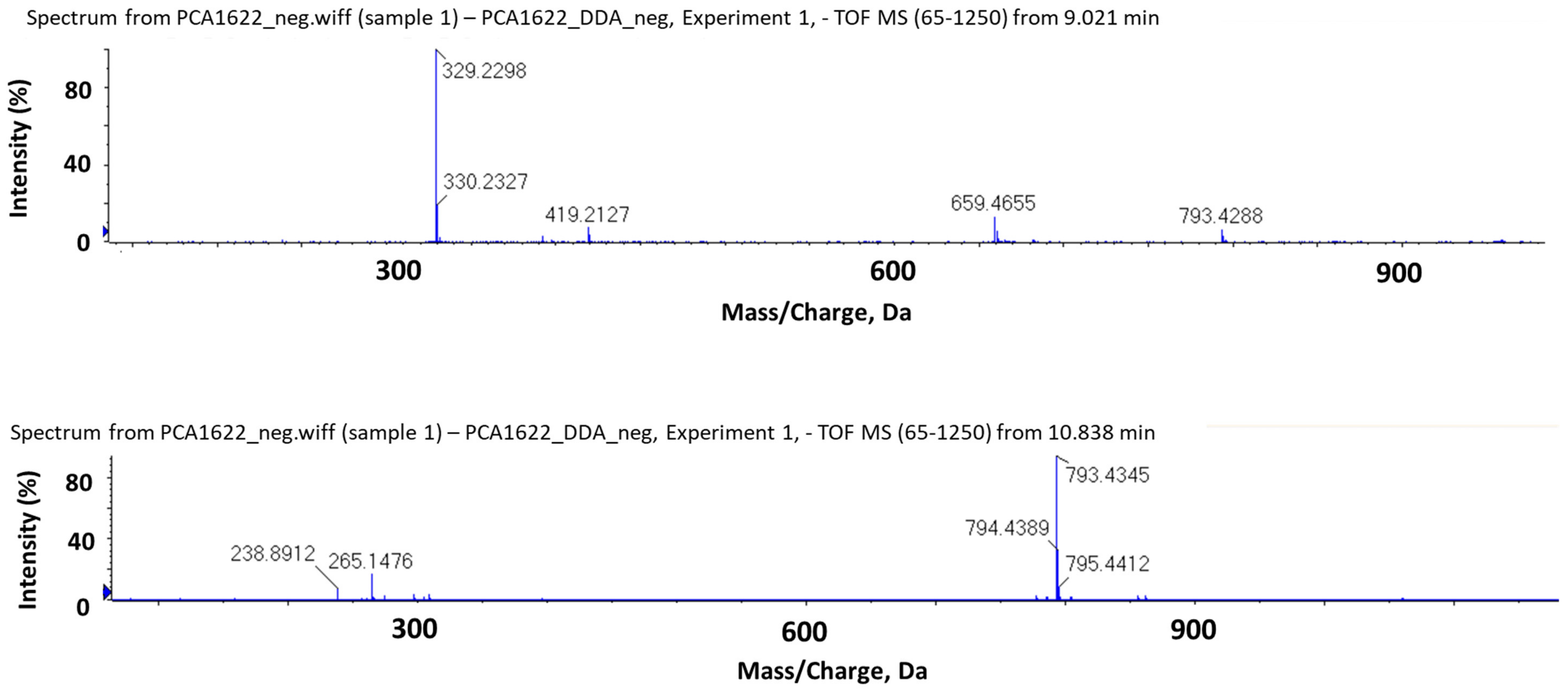
Spectrum from PCA1622_neg.wiff (sample 1) – PCA1622_DDA_neg, Experiment 1, - TOF MS (65-1250) from 9.021 min
Intensity (%)
80
40
0
329.2298
330.2327
419.2127
659.4655
793.4288
300
600
900
Mass/Charge, Da
Spectrum from PCA1622_neg.wiff (sample 1) – PCA1622_DDA_neg, Experiment 1, - TOF MS (65-1250) from 10.838 min
Intensity (%)
80
40
0
238.8912
265.1476
793.4345
794.4389
795.4412
300
600
900
Mass/Charge, Da

C

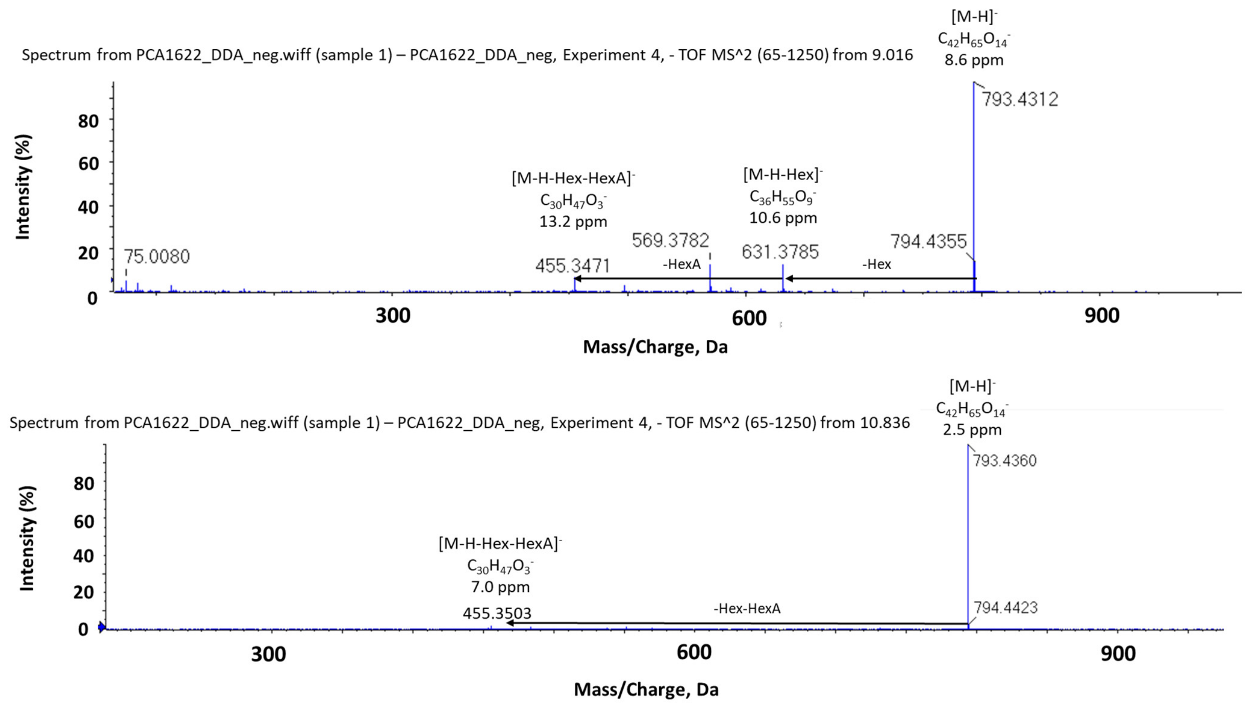

Figure S11. Extracted ion chromatogram m/z 793.4400 ± 0.02 (A), the MS spectra (B) and MS/MS fragmentation patterns (C) of the compounds 12, 18 annotated in the total ethanolic extract of *Aralia elata var. mandshurica* (*Rupr. & Maxim.*) J. Wen roots as oleanolic acid-3-*O*-(hexosyl)-28-1-hexouronide ester isomers t_R 9.02 and 10.84, respectively. The analysis relied on RP-UHPLC-QqTOF-MS accomplished with a Waters ACQUITY I-Class UPLC System (Waters GmbH, Eschborn, Germany) coupled on-line to a Triple-TOF6600 hybrid mass spectrometer (Sciex, Darmstadt, Germany) in the negative ion mode. Metabolites were annotated by reversed phase ultra-high performance liquid chromatography-mass spectrometry and tandem mass spectrometry (RP-UHPLC-QqTOF-MS and MS/MS) in targeted data-dependent acquisition (DDA) experiments (with the inclusion list containing the *m/z* of all predicted $[M-H]^-$ ions annotated at the MS1 level).

A

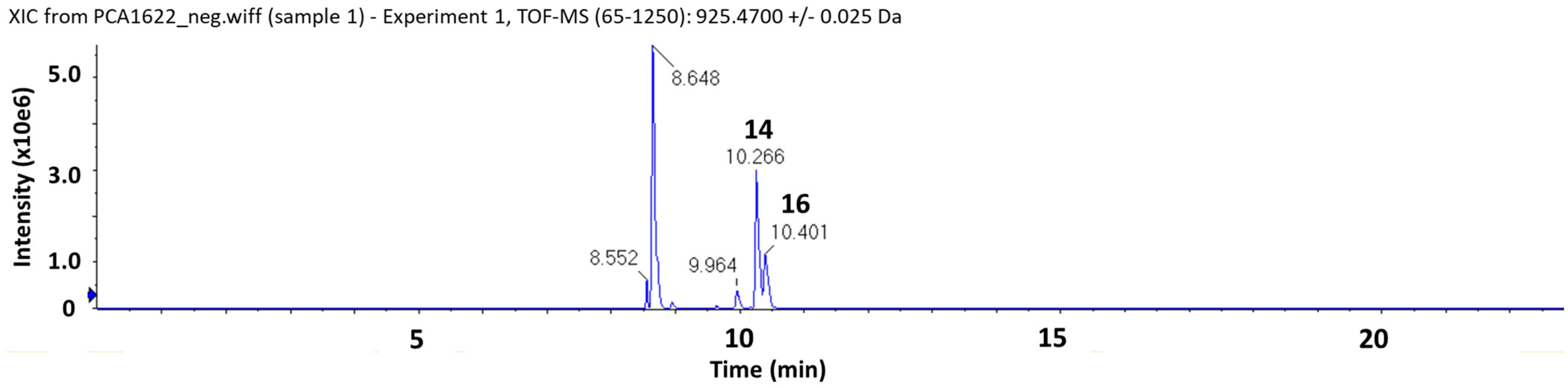
XIC from PCA1622_neg.wiff (sample 1) - Experiment 1, TOF-MS (65-1250): 925.4700 +/- 0.025 Da
Intensity (x10e6)
5.0
3.0
1.0
0
8.648
14
10.266
16
10.401
8.552
9.964
5
10
15
20
Time (min)

B

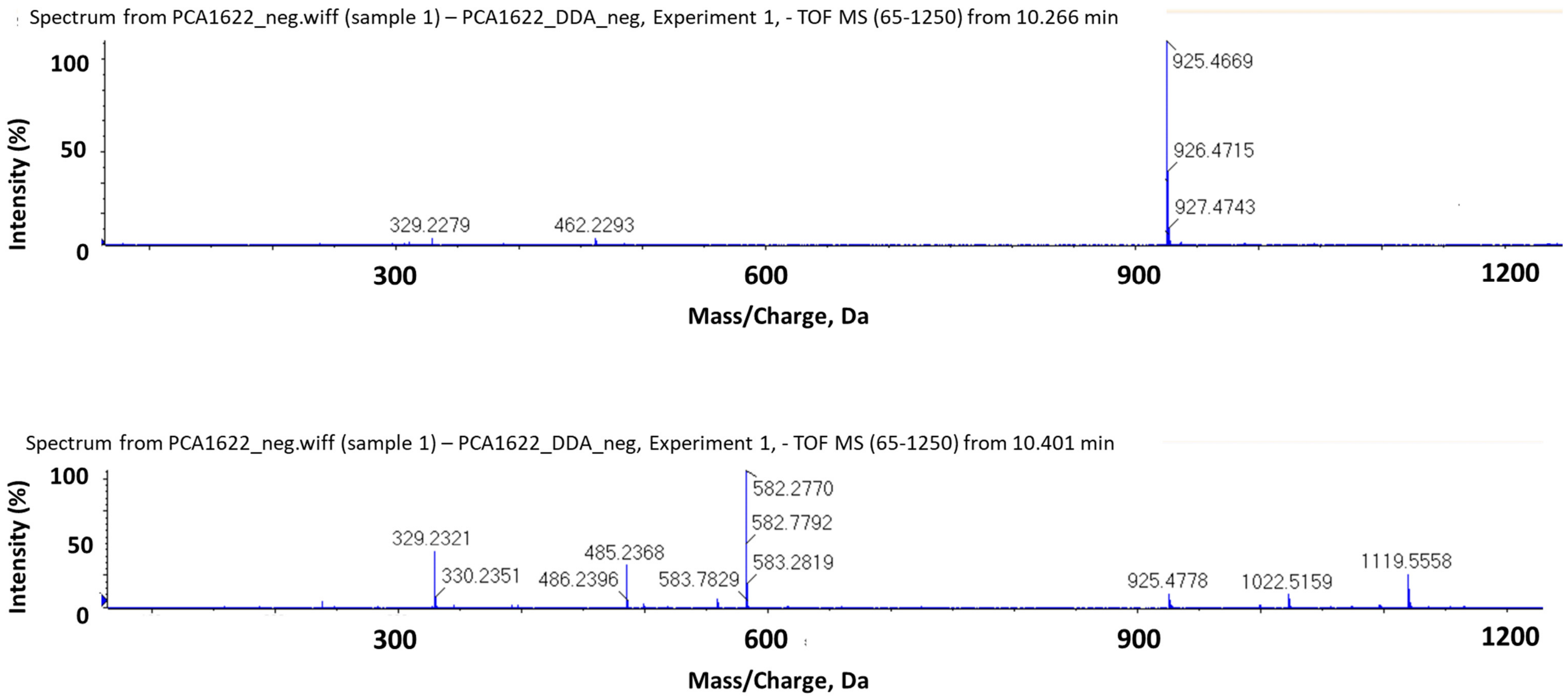

Spectrum from PCA1622_neg.wiff (sample 1) – PCA1622_DDA_neg, Experiment 1, - TOF MS (65-1250) from 10.266 min
Intensity (%)
100
50
0
925.4669
926.4715
927.4743
329.2279
462.2293
300
600
900
1200
Mass/Charge, Da
Spectrum from PCA1622_neg.wiff (sample 1) – PCA1622_DDA_neg, Experiment 1, - TOF MS (65-1250) from 10.401 min
Intensity (%)
100
50
0
582.2770
582.7792
583.2819
583.7829
329.2321
330.2351
485.2368
486.2396
925.4778
1022.5159
1119.5558
300
600
900
1200
Mass/Charge, Da

C

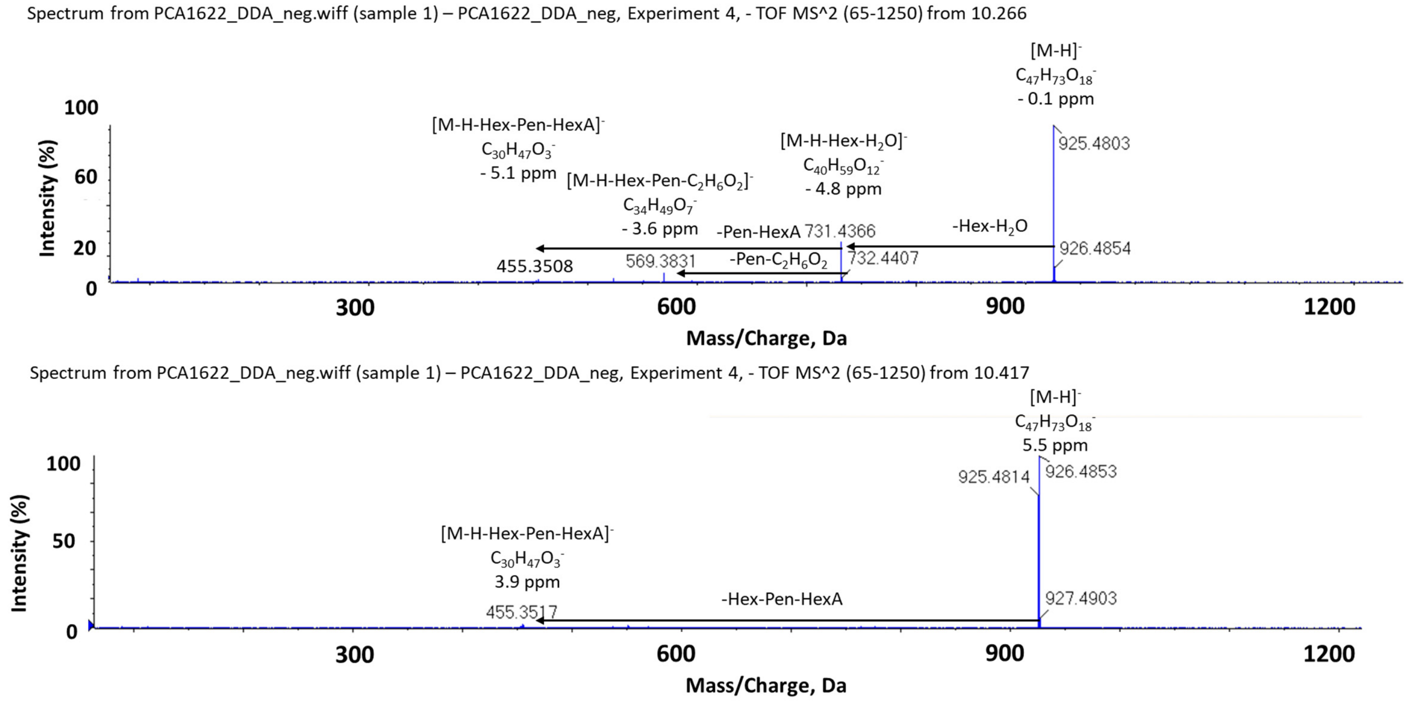

Figure S12. Extracted ion chromatogram m/z 925.4800 ± 0.02 (A), the MS spectra (B) and MS/MS fragmentation patterns (C) of the compounds 14, 16 annotated in the total ethanolic extract of *Aralia elata var. mandshurica* (*Rupr. & Maxim.*) J. Wen roots as araloside A isomers t_R 10.266 and 10.401, respectively. The analysis relied on RP-UHPLC-QqTOF-MS accomplished with a Waters ACQUITY I-Class UPLC System (Waters GmbH, Eschborn, Germany) coupled on-line to a Triple-TOF6600 hybrid mass spectrometer (Sciex, Darmstadt, Germany) in the negative ion mode. Metabolites were annotated by reversed phase ultra-high performance liquid chromatography-mass spectrometry and tandem mass spectrometry (RP-UHPLC-QqTOF-MS and MS/MS) in targeted data-dependent acquisition (DDA) experiments (with the inclusion list containing the *m/z* of all predicted $[M-H]^-$ ions annotated at the MS1 level).

A

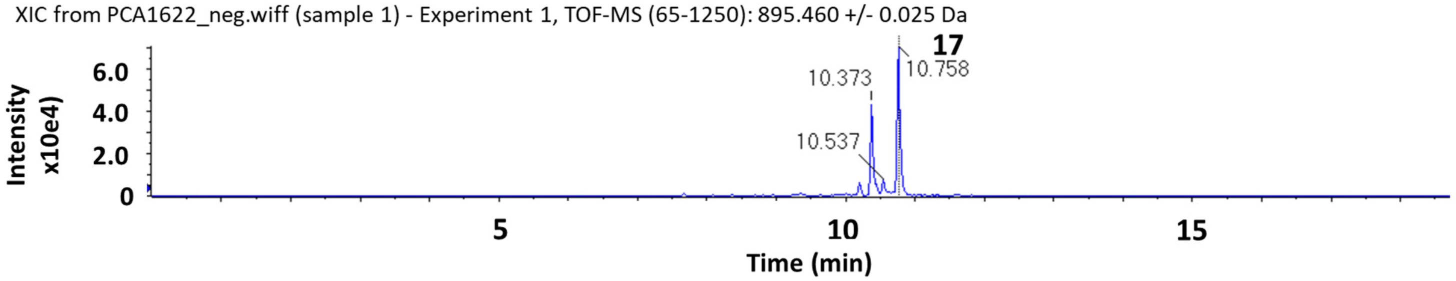

XIC from PCA1622_neg.wiff (sample 1) - Experiment 1, TOF-MS (65-1250): 895.460 +/- 0.025 Da
17
10.758
10.373
10.537
6.0
4.0
2.0
0
Intensity x10e4)
5
10
15
Time (min)

B

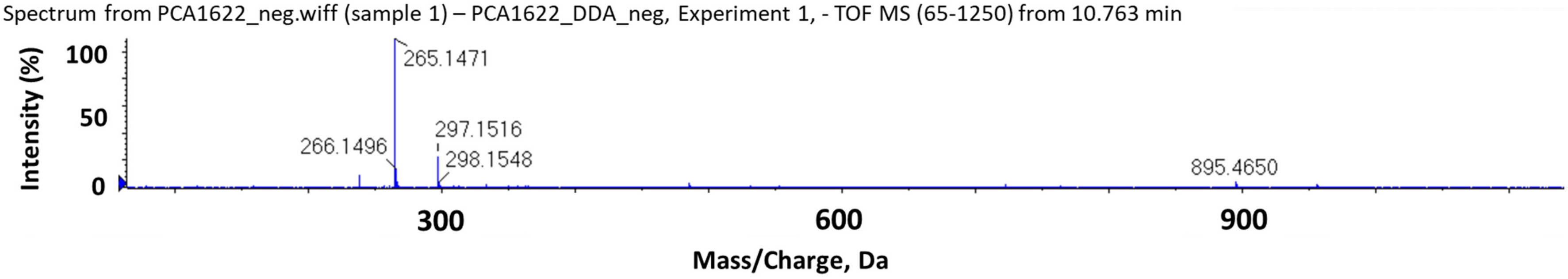

Spectrum from PCA1622_neg.wiff (sample 1) – PCA1622_DDA_neg, Experiment 1, - TOF MS (65-1250) from 10.763 min
100
50
0
Intensity (%)
265.1471
297.1516
266.1496
298.1548
895.4650
300
600
900
Mass/Charge, Da

C

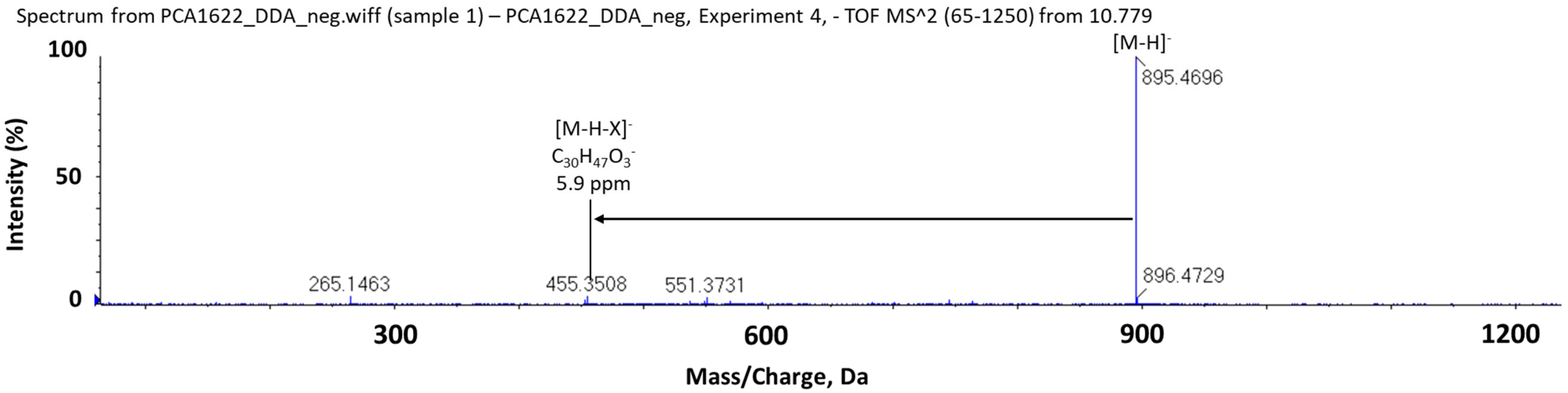

Figure S13. Extracted ion chromatogram m/z 895.4500 ± 0.02 (A), the MS spectra (B) and MS/MS fragmentation patterns (C) of the compounds 17 annotated in the total ethanolic extract of *Aralia elata var. mandshurica* (*Rupr. & Maxim.*) J. Wen roots as oleanolic acid unknown derivatives. The analysis relied on RP-UHPLC-QqTOF-MS accomplished with a Waters ACQUITY I-Class UPLC System (Waters GmbH, Eschborn, Germany) coupled on-line to a Triple-TOF6600 hybrid mass spectrometer (Sciex, Darmstadt, Germany) in the negative ion mode. Metabolites were annotated by reversed phase ultra-high performance liquid chromatography-mass spectrometry and tandem mass spectrometry (RP-UHPLC-QqTOF-MS and MS/MS) in targeted data-dependent acquisition (DDA) experiments (with the inclusion list containing the *m/z* of all predicted $[M-H]^-$ ions annotated at the MS1 level).

A

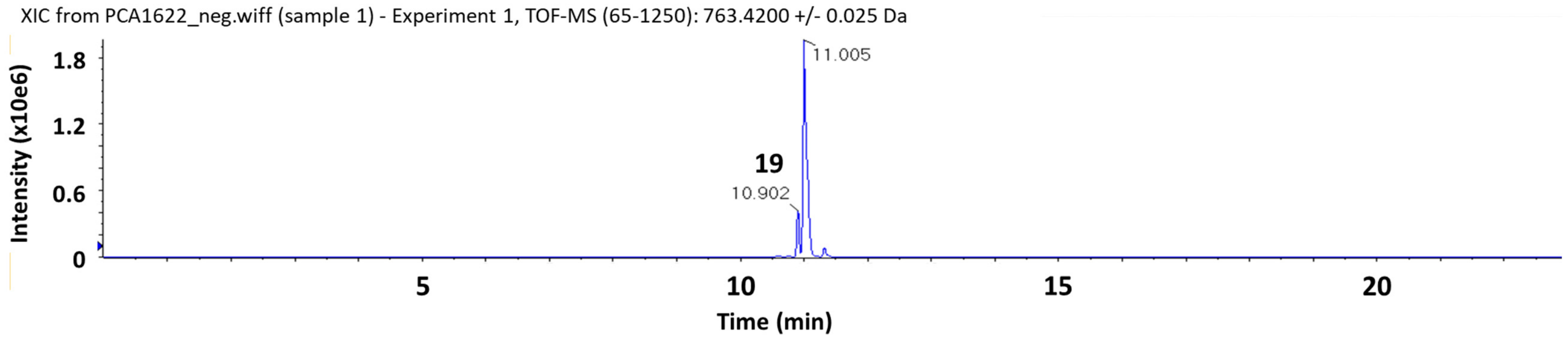

XIC from PCA1622_neg.wiff (sample 1) - Experiment 1, TOF-MS (65-1250): 763.4200 +/- 0.025 Da
Intensity (x10e6)
1.8
1.2
0.6
0
11.005
19
10.902
5
10
15
20
Time (min)

B

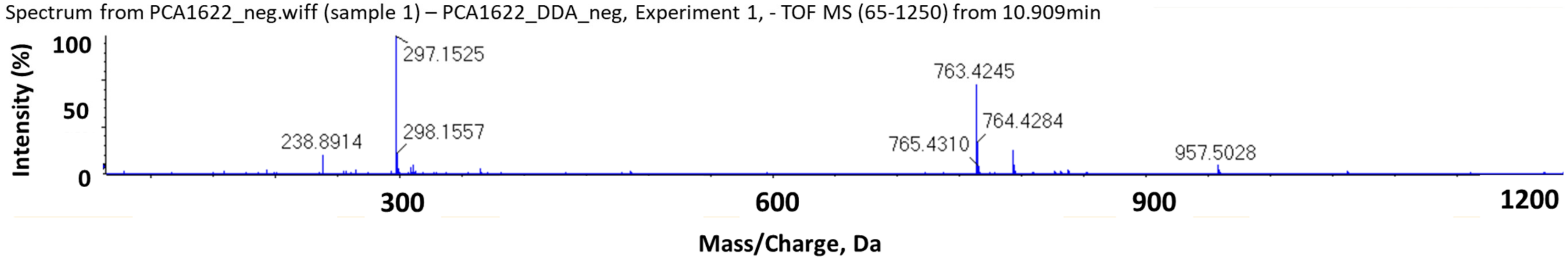

Spectrum from PCA1622_neg.wiff (sample 1) – PCA1622_DDA_neg, Experiment 1, - TOF MS (65-1250) from 10.909min
Intensity (%)
100
50
0
297.1525
298.1557
238.8914
763.4245
764.4284
765.4310
957.5028
300
600
900
1200
Mass/Charge, Da

C

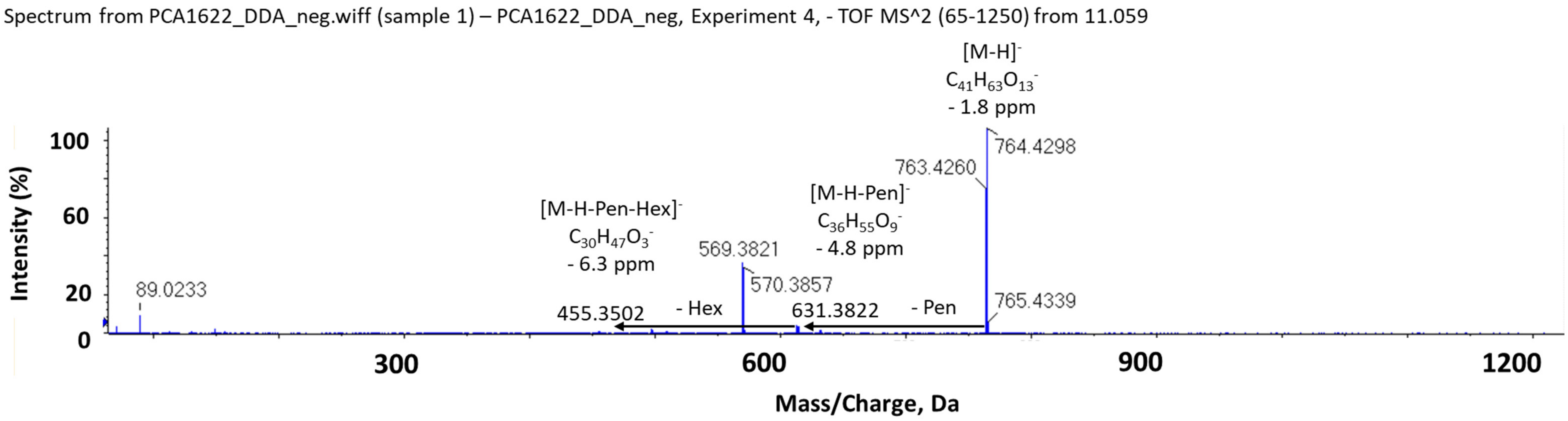

Figure S14. Extracted ion chromatogram m/z 763.4300 ± 0.02 (A), the MS spectra (B) and MS/MS fragmentation patterns (C) of the compounds 19 annotated in the total ethanolic extract of *Aralia elata var. mandshurica* (*Rupr. & Maxim.*) J. Wen roots as oleanolic acid 3-*O*-hexuronide-(1-3-pentafuranoside). The analysis relied on RP-UHPLC-QqTOF-MS accomplished with a Waters ACQUITY I-Class UPLC System (Waters GmbH, Eschborn, Germany) coupled on-line to a Triple-TOF6600 hybrid mass spectrometer (Sciex, Darmstadt, Germany) in the negative ion mode. Metabolites were annotated by reversed phase ultra-high performance liquid chromatography-mass spectrometry and tandem mass spectrometry (RP-UHPLC-QqTOF-MS and MS/MS) in targeted data-dependent acquisition (DDA) experiments (with the inclusion list containing the *m/z* of all predicted [M-H]$^-$ ions annotated at the MS1 level).

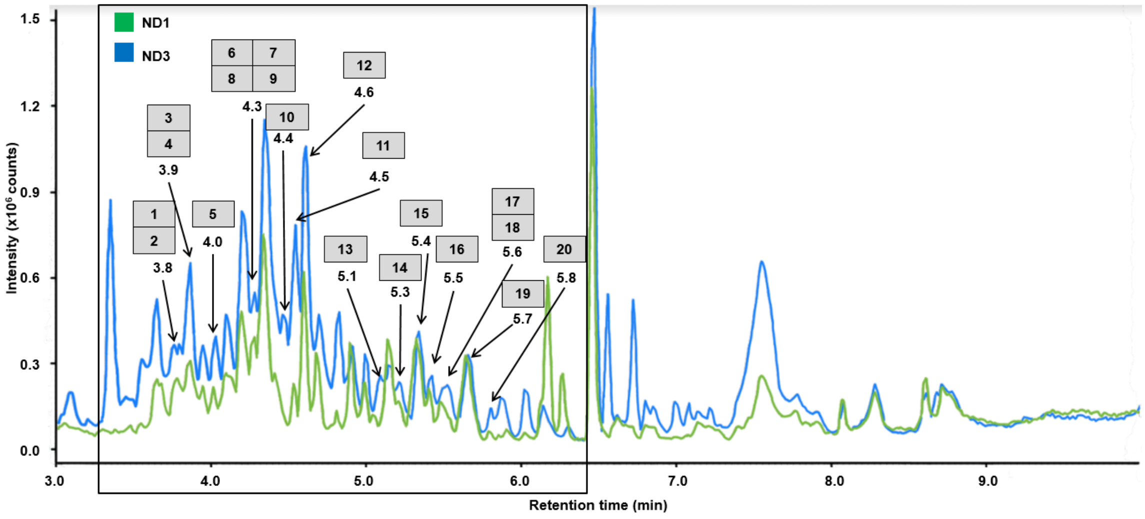

Figure S15. The full t_R range obtained in the chromatograms of ND1 and ND3 extracts of whole roots of *A. elata* (peak numbers correspond to compounds listed in Table 1).

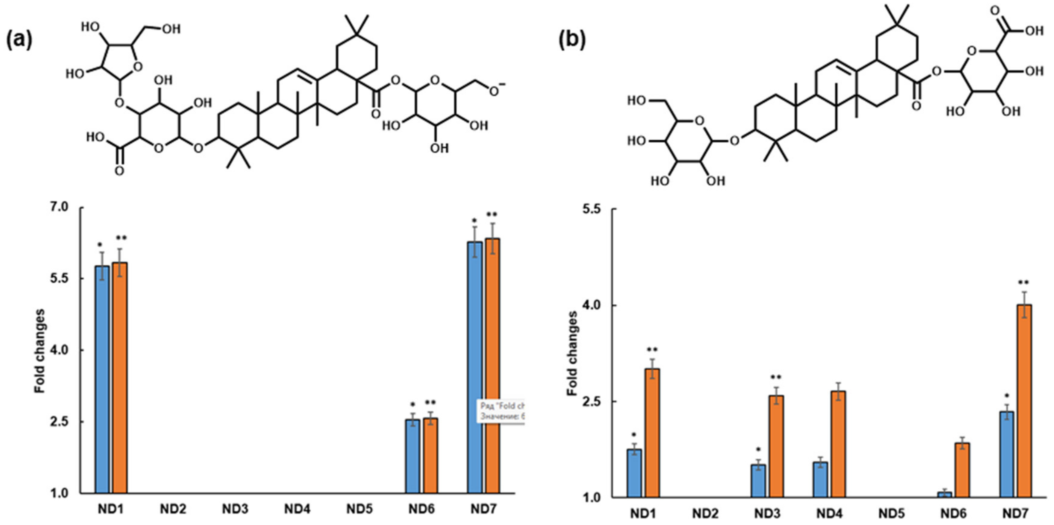

Figure S16. Structures and relative recoveries of **14** (araloside isomer 1 (a)), **18** (oleanolic acid-3-*O*-(hexosyl)-28-1-hexouronide ester isomer 1 (b)), *13*, expressed as the difference (fold) in comparison to those observed in aqueous and ethanolic extracts. Fold changes comparison with water (blue), fold changes comparison with ethanol (orange), * (for recoveries in relation to water) or ** (for recoveries in relation to ethanol) - $p \leq 0.05$ vs. control. The compounds are numbered as in Table 1. ND1 – NADES with choline chloride and malic acid (molar ratio 1:1), ND2 – NADES with the molar ratio of choline chloride and malic acid of 1:2, ND3 – NADES with the molar ratio of choline chloride and lactic acid of 1:3, ND4 - NADES with the molar ratio of choline chloride and lactic acid of 1:3+ 30% (*v/v*) water, ND6 – NADES with the molar ratio of sorbitol and malic acid of 1:1 + 10% (*v/v*) water, ND7 - NADES with the molar ratio of sorbitol and malic acid of 1:2+20% (*v/v*) water.

References

1. Wang, Q.-H.; Zhang, J.; Ma, X.; Ye, X.-Y.; Yang, B.-Y.; Xia, Y.-G.; Kuang, H.-X. A New Triterpenoid Saponin from the Leaves of Aralia Elata. *Chinese Journal of Natural Medicines* 2011, *9* (1), 17–21. https://doi.org/10.1016/S1875-5364(11)60012-5.
2. Han, F.; Liang, J.; Yang, B.-Y.; Kuang, H.-X.; Xia, Y.-G. Identification and Comparison of Triterpene Saponins in Aralia Elata Leaves and Buds by the Energy-Resolved MSAll Technique on a Liquid Chromatography/Quadrupole Time-of-Flight Mass Spectrometry. *Journal of Pharmaceutical and Biomedical Analysis* 2021, *203*, 114176. https://doi.org/10.1016/j.jpba.2021.114176.
3. Jiang, Y. T.; Xu, S. X.; Gu, X. H.; Ren, L.; Chen, Y. J.; Yao, X. S.; Miao, Z. C. Studies on the chemical constituents from *Aralia elata*. *Yao Xue Xue Bao* 1992, *27* (7), 528–532.
4. Xu, Y.; Liu, J.; Zeng, Y.; Jin, S.; Liu, W.; Li, Z.; Qin, X.; Bai, Y. Traditional Uses, Phytochemistry, Pharmacology, Toxicity and Quality Control of Medicinal Genus *Aralia*: A Review. *Journal of Ethnopharmacology* 2022, *284*, 114671. https://doi.org/10.1016/j.jep.2021.114671.
5. Lin, G.; Yang, J. -s. Studies on the Chemical Constituents of *Aralia Decaisneane* II. *CHINESE PHARMACEUTICAL JOURNAL-BEIJING-* 2004, *39* (8), 575–578.
6. Miyase, T.; Shiokawa, K.-I.; Zhang, D. M.; Ueno, A. Araliasaponins I–XI, Triterpene Saponins from the Roots of *Aralia Decaisneana*. *Phytochemistry* 1996, *41* (5), 1411–1418.
7. Miyase, T.; Sutoh, N.; Zhang, D. M.; Ueno, A. Araliasaponins XII-XVIII, Triterpene Saponins from the Roots of *Aralia Chinensis*. *Phytochemistry* 1996, *42* (4), 1123–1130.
8. Yen, P. H.; Cuc, N. T.; Huong, P. T. T.; Nhiem, N. X.; Hong Chuong, N. T.; Lien, G. T. K.; Huu Tai, B.; Tuyen, N. V.; Van Minh, C.; Van Kiem, P. Araliaarmoside: A New Triterpene Glycoside Isolated from the Leaves of *Aralia Armata*. *Natural Product Communications* 2020, *15* (9), 1934578X20953300.

9. Gao, R.; Liao, M.; Huang, X.; Chen, Y.; Yang, G.; Li, J. Six New Triterpene Derivatives from *Aralia Chinensis* Var. Dasyphylloides. *Molecules* 2016, *21* (12), 1700.

10. Song, S.; Nakamura, N.; HATTORI, M.; XU, S. Four New Saponins from the Root Bark of *Aralia Elata*. *Chemical and pharmaceutical bulletin* 2000, *48* (6), 838–842.

11. Kochetkov, N. K.; Khorlin, A. J.; Vaskovsky, V. E. The Structures of Aralosides A and B. *Tetrahedron Letters* 1962, *3* (16), 713–716. https://doi.org/10.1016/S0040-4039(00)70938-5.

12. Sun, W. J.; Zhang, D. K.; Sha, Z. F.; Zhang, H. L.; Zhang, X. L. Studies on the Saponins from the Root Bark of *Aralia Chinensis* L. *Yao xue xue bao= Acta Pharmaceutica Sinica* 1991, *26* (3), 197–202.

13. Yu, S.-S.; Yu, D.-Q.; Liang, X.-T. Triterpenoid Saponins from the Roots of *Aralia Spinifolia*. *Journal of natural products* 1994, *57* (7), 978–982.

14. Yen, P. H.; Chuong, N. T. H.; Lien, G. T. K.; Cuc, N. T.; Nhiem, N. X.; Thanh, N. T. V.; Tai, B. H.; Seo, Y.; Namkung, W.; Park, S. Oleanane-Type Triterpene Saponins from *Aralia Armata* Leaves and Their Cytotoxic Activity. *Natural Product Research* 2021, *36* (1), 142–149.

15. Kasprzyk, Z.; Wojciechowski, Z. The Structure of Triterpenic Glycosides from the Flowers of *Calendula Officinalis* L. *Phytochemistry* 1967, *6* (1), 69–75. https://doi.org/10.1016/0031-9422(67)85009-X.

16. Zaki, A. A.; Qiu, L. Machaerinic Acid 3-O-β-D-Glucuronopyranoside from *Calendula Officinalis*. *Natural product research* 2020, *34* (20), 2938–2944.

17. Liang, X. F.; Zhao, Y. Y.; Liu, X. Z.; Yang, X. J.; Fan, Y.; Guo, D. Y.; Song, X. M.; Song, B. Isolation and Identification of Chemical Constituents from *Aralia Taibaiensis* Cortex. *Chin J Exper Trad Med Form* 2018, *20* (24), 56–61.

18. Yoshikawa, M.; Harada, E.; Matsuda, H.; Murakami, T.; Yamahara, J.; Murakami, N. Elatosides A and B, Potent Inhibitors of Ethanol Absorption in Rats from the Bark of *Aralia*

Elata SEEM.: The Structure-Activity Relationships of Oleanolic Acid Oligoglycosides. *Chem. Pharm. Bull.* 1993, *41* (11), 2069–2071. https://doi.org/10.1248/cpb.41.2069.

19. Song, S.-J.; Nakamura, N.; Ma, C.-M.; Hattori, M.; Xu, S.-X. Five Saponins from the Root Bark of *Aralia Elata*. *Phytochemistry* 2001, *56* (5), 491–497. https://doi.org/10.1016/S0031-9422(00)00379-4.
20. Guo, M.; Zhang, L.; Liu, Z. Analysis of Saponins from Leaves of *Aralia Elata* by Liquid Chromatography and Multi-Stage Tandem Mass Spectrometry. *ANAL. SCI.* 2009, *25* (6), 753–755. https://doi.org/10.2116/analsci.25.753.
21. Shuyu, C.; Xianggao, L.; Chongxi, Z. Chemical components of *Aralia elata*. *Journal of Jilin Agricultural University (China)* 1992.
22. Ma, Z.; Song, S.; Xu, S. Two new saponins from *Aralia elata* (Miq.) see. *Chinese Journal of Medicinal Chemistry* 2004, *14* (1), 47–48.
23. Zhang, Y.; Peng, Y.; Li, L.; Zhao, L.; Hu, Y.; Hu, C.; Song, S. Studies on Cytotoxic Triterpene Saponins from the Leaves of *Aralia Elata*. *Food Chemistry* 2013, *138* (1), 208–213. https://doi.org/10.1016/j.foodchem.2012.10.041.
24. Kuang, H.-X.; Sun, H.; Zhang, N.; Okada, Y.; Okuyama, T. Two New Saponins, Congmuyenosides A and B, from the Leaves of *Aralia Elata* Collected in Heilongjiang, China. *Chem. Pharm. Bull.* 1996, *44* (11), 2183–2185. https://doi.org/10.1248/cpb.44.2183.
25. Kim, J. S.; Shim, S. H.; Chae, S.; Han, S. J.; Kang, S. S.; Son, K. H.; Chang, H. W.; Kim, H. P.; Bae, K. Saponins and Other Constituents from the Leaves of *Aralia Elata*. *Chem. Pharm. Bull.* 2005, *53* (6), 696–700. https://doi.org/10.1248/cpb.53.696.
26. Xi, F.-M.; Li, C.-T.; Han, J.; Yu, S.-S.; Wu, Z.-J.; Chen, W.-S. Thiophenes, Polyacetylenes and Terpenes from the Aerial Parts of *Eclipata Prostrata*. *Bioorganic & Medicinal Chemistry* 2014, *22* (22), 6515–6522. https://doi.org/10.1016/j.bmc.2014.06.051.

27. Matsuo, Y.; Watanabe, K.; Mimaki, Y. Triterpene Glycosides from the Underground Parts of *Caulophyllum Thalictroides*. *J. Nat. Prod.* 2009, *72* (6), 1155–1160. https://doi.org/10.1021/np900164b.
28. Nhiem, N. X.; Lim, H. Y.; Kiem, P. V.; Minh, C. V.; Thu, V. K.; Tai, B. H.; Quang, T. H.; Song, S. B.; Kim, Y. H. Oleanane-Type Triterpene Saponins from the Bark of *Aralia Elata* and Their NF-KB Inhibition and PPAR Activation Signal Pathway. *Bioorganic & Medicinal Chemistry Letters* 2011, *21* (20), 6143–6147. https://doi.org/10.1016/j.bmcl.2011.08.024.
29. Yoshikawa, M.; Yoshizumi, S.; Ueno, T.; Matsuda, H.; Murakami, T.; Yamahara, J.; Murakami, N. Medicinal Foodstuffs. I. Hypoglycemic Constituents from a Garnish Foodstuff "Taranome," the Young Shoot of *Aralia Elata* SEEM.: Elatosides G, H, I, J, and K. *Chem. Pharm. Bull.* 1995, *43* (11), 1878–1882. https://doi.org/10.1248/cpb.43.1878.
30. Ahmad, V.; Perveen, S.; Bano, S. Guaiacin A and B from the Leaves of *Guaiacum Officinale*. *Planta Med* 1989, *55* (03), 307–308. https://doi.org/10.1055/s-2006-962014.
31. Shao, C.-J.; Kasai, R.; Xu, J.-D.; Tanaka, O. Saponins from Roots of Kalopanax Septemlobus (THUNB.)KOIDZ., Ciqiu: Structures of Kalopanax-Saponins C, D, E and F. *Chem. Pharm. Bull.* 1989, *37* (2), 311–314. https://doi.org/10.1248/cpb.37.311.
32. Tian, Y.; Zhang, X.; Liu, H.; Gong, D.; Li, X. Comparison of the Nutritional and Phytochemical Composition and Antioxidant Activities of *Aralia Elata* (Miq.) Seem Fruits in Northeast China. *Arabian Journal of Chemistry* 2021, *14* (12), 103448. https://doi.org/10.1016/j.arabjc.2021.103448.
33. Sakai, S.; Katsumata(Nee Ohtsuka), M.; Satoh, Y.; Nagasao, M.; Miyakoshi, M.; Ida, Y.; Shoji, J. Oleanolic Acid Saponins from Root Bark of *Aralia Elata*. *Phytochemistry* 1994, *35* (5), 1319–1324. https://doi.org/10.1016/S0031-9422(00)94846-5.
34. Kuljanabhagavad, T.; Thongphasuk, P.; Chamulitrat, W.; Wink, M. Triterpene Saponins from *Chenopodium Quinoa* Willd. *Phytochemistry* 2008, *69* (9), 1919–1926. https://doi.org/10.1016/j.phytochem.2008.03.001.

35. Waffo-Téguo, P.; Voutquenne, L.; Thoison, O.; Dumontet, V.; Nguyen, V. H.; Lavaud, C. Acetylated Glucuronide Triterpene Bidesmosidic Saponins from *Symplocos Glomerata*. *Phytochemistry* 2004, *65* (6), 741–750. https://doi.org/10.1016/j.phytochem.2004.01.012.

36. Yoshikawa, M.; Murakami, T.; Harada, E.; Murakami, N.; Yamahara, J.; Matsuda, H. Bioactive Saponins and Glycosides. VI. Elatosides A and B, Potent Inhibitors of Ethanol Absorption, from the Bark of *Aralia Elata* SEEM. (Araliaceae): The Structure-Requirement in Oleanolic Acid Glucuronide-Saponins for the Inhibitory Activity. *Chem. Pharm. Bull.* 1996, *44* (10), 1915–1922. https://doi.org/10.1248/cpb.44.1915.

37. Tang, H. F.; Yi, Y. H.; Wang, Z. Z.; Jiang, Y. P.; Li, Y. Q. Oleanolic acid saponins from the root bark of *Aralia taibaiensis*. *Yao Xue Xue Bao* 1997, *32* (9), 685–690.

38. Miyase, T.; Kohsaka, H.; Ueno, A. Tragopogonosides A-I, Oleanane Saponins from *Tragopogon Pratensis*. *Phytochemistry* 1992, *31* (6), 2087–2091. https://doi.org/10.1016/0031-9422(92)80368-O.
